# Tankyrase Requires SAM Domain-Dependent Polymerization to Support Wnt-β-Catenin Signaling

**DOI:** 10.1016/j.molcel.2016.06.019

**Published:** 2016-08-04

**Authors:** Laura Mariotti, Catherine M. Templeton, Michael Ranes, Patricia Paracuellos, Nora Cronin, Fabienne Beuron, Edward Morris, Sebastian Guettler

**Affiliations:** 1 Division of Structural Biology, The Institute of Cancer Research (ICR), London SW7 3RP, UK; 2 Division of Cancer Biology, The Institute of Cancer Research (ICR), London SW7 3RP, UK

## Abstract

The poly(ADP-ribose) polymerase (PARP) Tankyrase (TNKS and TNKS2) is paramount to Wnt-β-catenin signaling and a promising therapeutic target in Wnt-dependent cancers. The pool of active β-catenin is normally limited by destruction complexes, whose assembly depends on the polymeric master scaffolding protein AXIN. Tankyrase, which poly(ADP-ribosyl)ates and thereby destabilizes AXIN, also can polymerize, but the relevance of these polymers has remained unclear. We report crystal structures of the polymerizing TNKS and TNKS2 sterile alpha motif (SAM) domains, revealing versatile head-to-tail interactions. Biochemical studies informed by these structures demonstrate that polymerization is required for Tankyrase to drive β-catenin-dependent transcription. We show that the polymeric state supports PARP activity and allows Tankyrase to effectively access destruction complexes through enabling avidity-dependent AXIN binding. This study provides an example for regulated signal transduction in non-membrane-enclosed compartments (signalosomes), and it points to novel potential strategies to inhibit Tankyrase function in oncogenic Wnt signaling.

## Highlights

• SAM domain crystal structures reveal mechanism of Tankyrase polymerization
• Catalysis-independent Tankyrase scaffolding drives Wnt-β-catenin signaling
• Tankyrase polymerization supports PARP activity and AXIN binding

## Graphical Abstract

**Figure:**
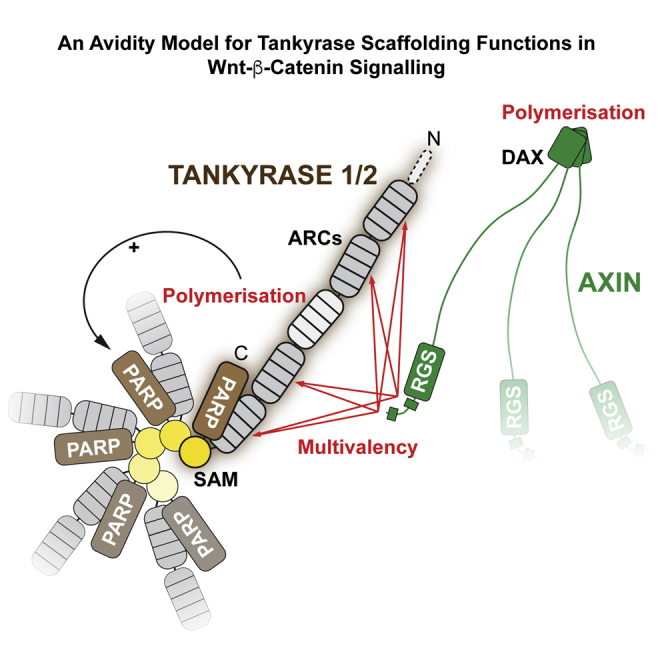

## Teaser

Catalysis-independent scaffolding by Tankyrase supports Wnt-β-catenin signaling. Scaffolding is mediated by AXIN-binding ankyrin repeat clusters and a polymerizing SAM domain. In a structure-function approach, Mariotti et al. show that polymerization supports Tankyrase PARP activity and enables avidity-dependent AXIN binding.

## Introduction

Signal transduction often occurs through large and transient multi-protein complexes. Polymerizing proteins can nucleate the assembly of higher-order structures termed signalosomes, which enable locally increased protein concentrations for efficient, transient, and spatially confined processes (Bienz, 2014, Wu, 2013). Wnt-β-catenin signaling, which is dysregulated in most colorectal cancers, provides prominent examples for signalosomes (Bienz, 2014, Polakis, 2012). At basal signaling, a destruction complex (DC) composed of the scaffolding proteins AXIN and adenomatous polyposis coli (APC), glycogen synthase kinase 3 (GSK3), and casein kinase 1 (CK1) captures and phosphorylates β-catenin to initiate its degradation (Stamos and Weis, 2013). AXIN is the central and concentration-limiting component of the DC (Lee et al., 2003, Stamos and Weis, 2013). Microscopically, DCs manifest as dynamic puncta with a filamentous sub-organization, so-called β-catenin degradasomes, whose formation is dependent on AXIN polymerization (Fiedler et al., 2011, de la Roche et al., 2014, Martino-Echarri et al., 2016, Thorvaldsen et al., 2015).

The poly(ADP-ribose) polymerases (PARPs) Tankyrase (TNKS and ARTD5) and Tankyrase 2 (TNKS2 and ARTD6) regulate Wnt-β-catenin signaling (Huang et al., 2009). We shall refer to TNKS and TNKS2 collectively as Tankyrase where principles apply to both. Tankyrase binds and poly(ADP-ribosyl)ates (PARylates) AXIN, targeting it for PAR-dependent ubiquitination (PARdU) and degradation to stabilize transcriptionally active β-catenin (Callow et al., 2011, DaRosa et al., 2015, Huang et al., 2009, Morrone et al., 2012, Zhang et al., 2011). Tankyrase buffers negative regulation of Wnt signaling by AXIN for robust pathway activation (Wang et al., 2016). Upon Wnt stimulation, AXIN PARylation by Tankyrase promotes its function in active signaling complexes (Yang et al., 2016).

**Figure 1:**
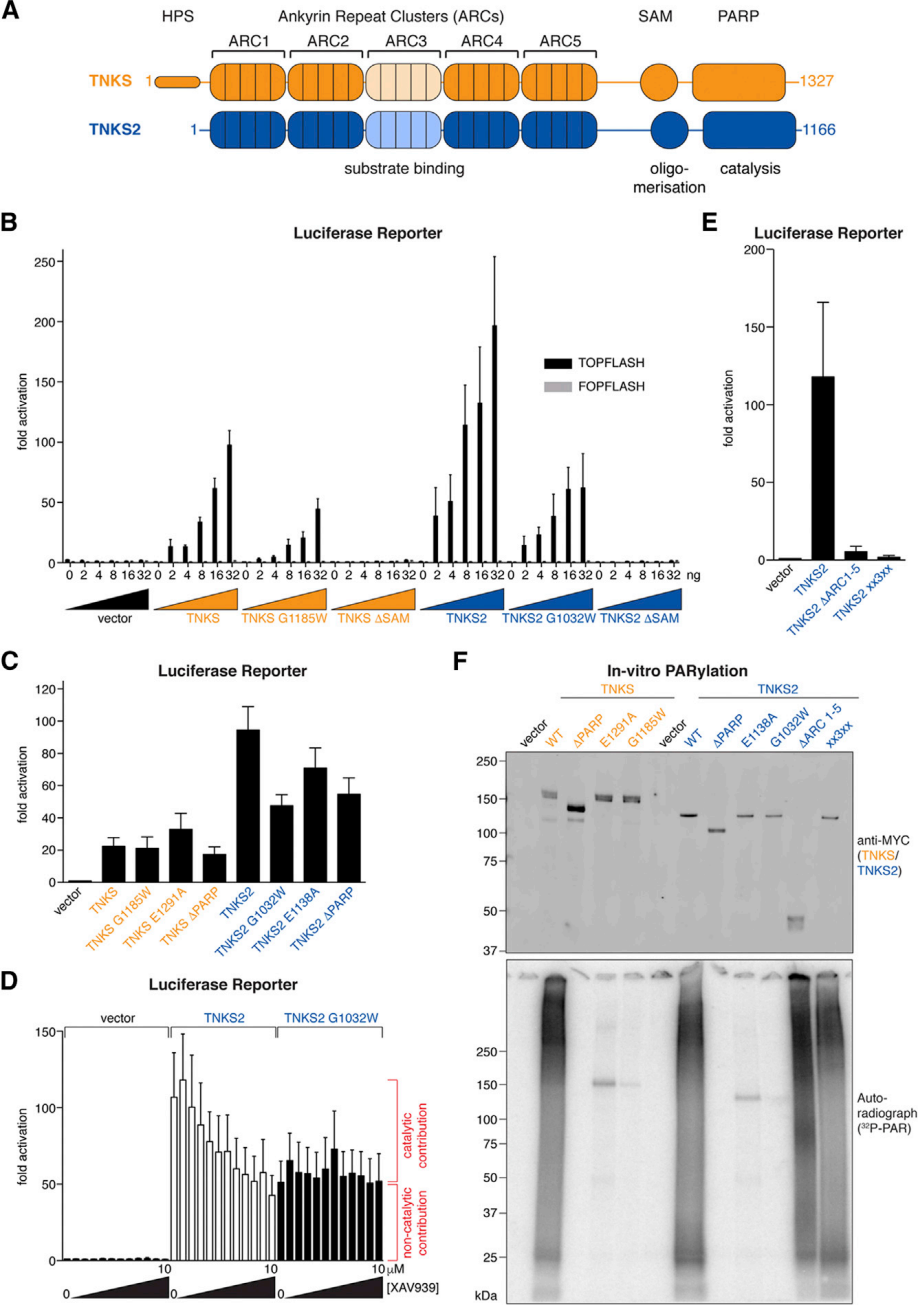
Requirement of ARCs and SAM Domains for Tankyrase-Driven Wnt Signaling (A) Domains of human TNKS and TNKS2 are shown. (B) Activation of β-catenin/TCF/LEF-dependent transcription by MYC_2_-Tankyrases in unstimulated HEK293T cells, assayed by TOPFlash and control FOPFlash reporters. Data are expressed relative to mean reporter activities obtained without MYC_2_ construct (seven samples in set; n = 3 duplicate experiments; error bars, SEM). (C) Transcription reporter assay as in (B), using 16 ng of MYC_2_-Tankyrase constructs. Fold activation is relative to vector only (n = 6 duplicate experiments; error bars, SEM). (D) Transcription reporter assay as in (C). Cells were treated with 9.8 nM to 10 μM XAV939 in a 2-fold dilution series. Data are expressed relative to reporter activity in the vector control in the absence of XAV939 (n = 3 duplicate experiments; error bars, SEM). See Figure S2A for TNKS2 PARylation assessment. (E) Transcription reporter assay as in (C) (n = 3 duplicate experiments; error bars, SEM). See Figure S1 for Tankyrase expression levels in luciferase reporter assays. (F) In vitro PARylation assay for the indicated immunoprecipitated MYC_2_-tagged Tankyrases. Top: western blot analysis of immunoprecipitates is shown; and bottom: autoradiograph is shown.

The two Tankyrases are highly similar (Hsiao and Smith, 2008, Smith et al., 1998) (Figure 1A), sharing a set of five ankyrin repeat clusters (ARCs) for substrate binding (Guettler et al., 2011, Seimiya et al., 2004), a sterile alpha motif (SAM) domain (De Rycker and Price, 2004, De Rycker et al., 2003), and a catalytic PARP domain (Rippmann et al., 2002). Tankyrase’s biological functions are complex (Haikarainen et al., 2014), and simultaneous loss of both Tankyrases results in embryonic lethality in mice (Chiang et al., 2008). Tankyrase contributes to telomere maintenance (Canudas et al., 2007, Dynek and Smith, 2004, Smith et al., 1998), which together with Wnt signaling is relevant to stem cell renewal, development, and certain types of cancer (Bernardes de Jesus and Blasco, 2013, Clevers et al., 2014). Given these functions and a dependency of BRCA1/2-deficient cancer cells on Tankyrase (McCabe et al., 2009), Tankyrase is a promising anti-cancer target (Haikarainen et al., 2014, Lehtiö et al., 2013, Riffell et al., 2012).

It is intriguing that Tankyrase, like AXIN, polymerizes (De Rycker and Price, 2004, De Rycker et al., 2003). Tankyrase polymerization is mediated by the SAM domain, a small helical fold highly prevalent in eukaryotes (Knight et al., 2011, Qiao and Bowie, 2005). The structural basis of Tankyrase polymerization and its function have remained unknown. Moreover, we currently lack insight into the regulation of Tankyrase activity.

Here we show that Tankyrase can induce Wnt-β-catenin signaling independently of its catalytic activity, through ARC- and SAM domain-dependent scaffolding. This redefines our view on pharmacologic inhibition of Tankyrase. Informed by crystal structures of the TNKS and TNKS2 SAM domains, we demonstrate that Tankyrase polymerization is critical for its function in Wnt signaling, required for full PARP activity, and necessary for efficient interaction with AXIN. We propose a model in which recruitment of Tankyrase to β-catenin DCs is promoted by avidity effects that arise from multivalency and polymerization inherent to the Tankyrase-AXIN complex.

## Results

### Tankyrase Requires ARCs and SAM Domain to Promote Wnt Signaling

To explore the domain requirements of Tankyrase for Wnt-β-catenin signaling, we measured β-catenin/TCF/LEF-dependent transcription in reporter assays. While basal Wnt signaling in HEK293T cells is low (Li et al., 2012), expression of TNKS or TNKS2 activated the reporter in a specific, dose-dependent manner (Figure 1B; see Figure S1 for protein expression levels). Intriguingly, reporter activation by either TNKS or TNKS2 was not abolished but merely reduced by ≈50% when poly- and mono(ADP-ribosyl)ation were inactivated by point mutation (G1185W^T1^ and G1032W^T2^; Figures 1B and 1F) (Yu et al., 2005). Likewise, mutation of a glutamate that is part of the catalytic H-Y-E triad (E1291A^T1^ and E1138A^T2^) (Hottiger et al., 2010), or deletion of the PARP domain altogether, did not abolish reporter activation (Figures 1C and 1F). Concordantly, saturating concentrations of the Tankyrase inhibitor XAV939 reduced TNKS2-dependent reporter activation only to a level that also was conferred by PARP-inactive TNKS2 G1032W^T2^ (Figures 1D and S2A). This suggests that both catalysis-dependent and -independent functions of Tankyrase contribute to Wnt signaling.

We hypothesized that scaffolding through the SAM domain and ARCs contributes to signaling. Deletion of the SAM domain fully abrogated Tankyrase-dependent reporter activation (Figure 1B), as did deletion of all ARCs or mutation of ARCs 1, 2, 4, and 5 (mutant xx3xx) to prevent substrate binding (Guettler et al., 2011) without impairing catalysis (Figures 1E and 1F). Our observations expand upon and are in line with previous deletion studies (Huang et al., 2009). ARCs and the SAM domain may collaborate in recruiting Tankyrase to AXIN and/or facilitate productive PARylation. Overexpression of Tankyrase-binding-deficient, but not wild-type (WT), AXIN1 impeded TNKS2-dependent Wnt signaling (Figure S2B). This is in agreement with Tankyrase activating Wnt-β-catenin signaling at the level of AXIN, and it illustrates the strong buffering capacity of Tankyrase toward AXIN (Huang et al., 2009, Wang et al., 2016).

### Polymerization of TNKS and TNKS2 SAM Domains

**Figure 2:**
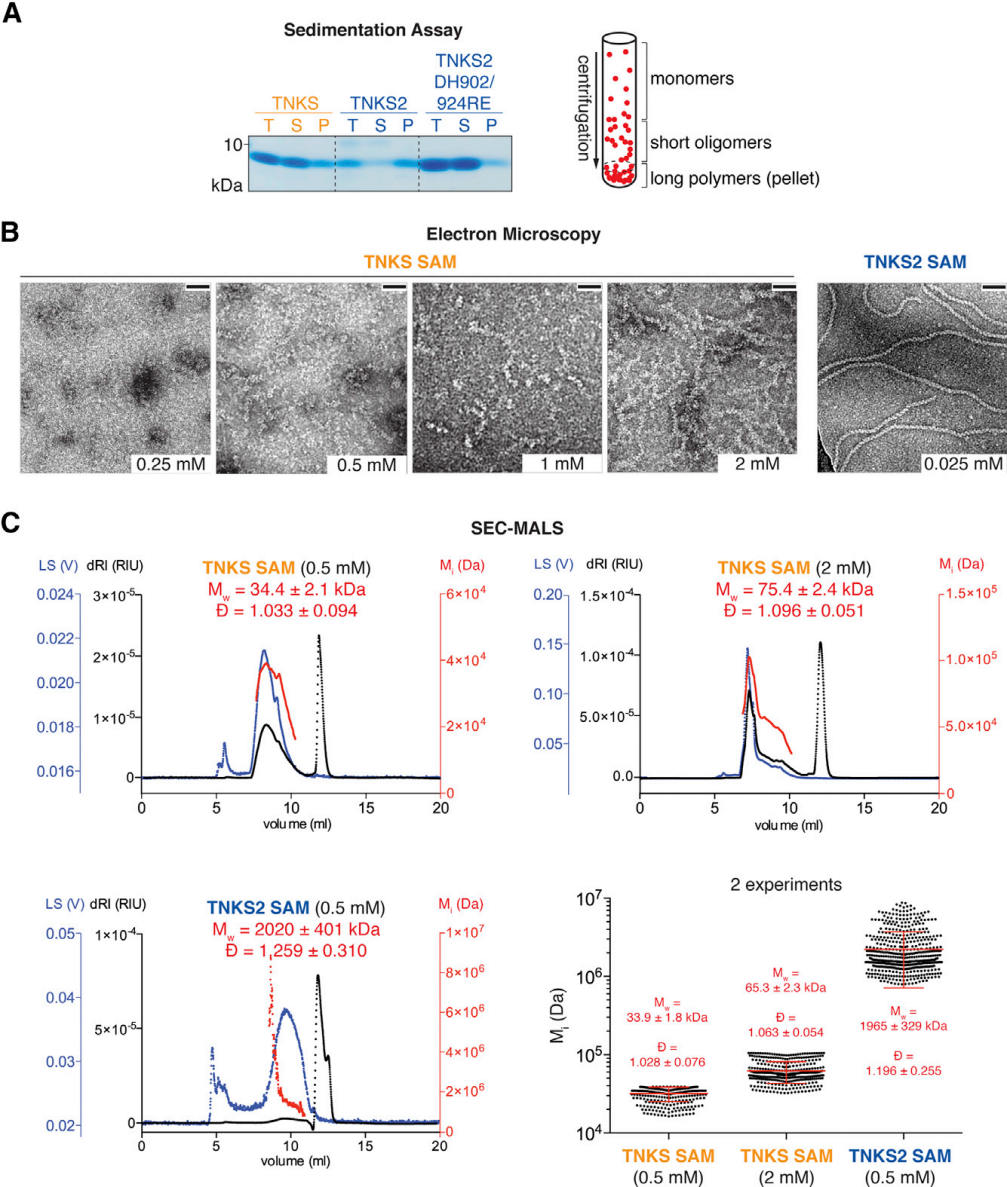
Polymerization of the TNKS and TNKS2 SAM Domains (A) Ultracentrifugation sedimentation assay. Purified SAM domains (25 μM) were centrifuged and total samples (T), supernatants (S), and pellets (P) were analyzed by SDS-PAGE and Coomassie staining. The diagram illustrates the assay principle. (B) Electron micrographs of SAM domains at the indicated concentrations are shown. Scale bars, 50 nm. (C) SEC-MALS. Chromatograms show one experiment with differential refractive index (dRI), light scattering (LS), and calculated molecular weight per slice i (M_i_). Weight-average molecular weights (M_w_) and dispersity (Ð) ± SD over peaks are indicated. See Figures S3B and S3C for eluate analyses by SDS-PAGE. The atypically delayed elution of the long TNKS2 SAM filaments likely reflects an interaction/entanglement with the column solid phase. Scatterplots combine data from two experiments with M_w_, Ð, and associated SD indicated. Plotted data points with mean and error bars (SD) refer to M_i_. See Figures S2 and S3 for further data.

AXIN binding by the Tankyrase ARCs is well understood (Guettler et al., 2011, Huang et al., 2009, Morrone et al., 2012). Conversely, it remains unclear how the SAM domain enables Tankyrase function in Wnt signaling and whether polymerization is involved. To study SAM domain polymerization, we performed ultracentrifugation sedimentation assays, in which polymers of purified SAM domains partition into the pellet (Figure 2A). While the TNKS2 SAM domain readily sedimented, that of TNKS did not, suggesting that TNKS SAM forms less stable polymers in vitro (Figure 2A). We observed filaments for both the TNKS2 and TNKS SAM domains by electron microscopy (EM), but TNKS SAM required higher concentrations to form visible filaments (Figure 2B). Based on a homology model (not shown), we generated a DH902/924RE^T2^ mutant of the TNKS2 SAM domain, which failed to sediment (Figure 2A).

We next analyzed highly purified SAM domains by size exclusion chromatography with in-line multi-angle light scattering (SEC-MALS), which is more sensitive than the sedimentation assay. When analyzed at 0.5 mM, the TNKS2 SAM domain (theoretical molecular weight [MW] ≈9 kDa) eluted as polydisperse assemblies of overall 1,965 ± 329 kDa, clearly indicating polymerization (Figure 2C). For 0.5 and 2 mM TNKS SAM, we detected polydisperse assemblies of 33.9 ± 1.8 kDa and 65.3 ± 2.3 kDa, respectively (Figure 2C), confirming that TNKS SAM also polymerizes, although to a lesser extent. We found that differential polymerization of the TNKS and TNKS2 SAM domains is largely due to a single amino acid difference (T1049^T1^ and R896^T2^, Figures S2C, S2D, S3A, and S3B). However, transcription reporter assays with TNKS/TNKS2 SAM domain chimeras and interconverting point mutants (T1049R^T1^ and R896T^T2^) showed that both SAM domains equally enable Tankyrase to drive Wnt signaling (Figure S2E). Thus, the SAM domain may either promote Tankyrase function independently of its polymerization, or the lower polymerization of TNKS may still be sufficient for Wnt signaling under the assay conditions. In the latter case, a substantial impairment of TNKS/TNKS2 polymerization would abrogate Tankyrase-dependent Wnt signaling.

### Crystal Structures of TNKS2 and TNKS SAM Domains

**Figure 3:**
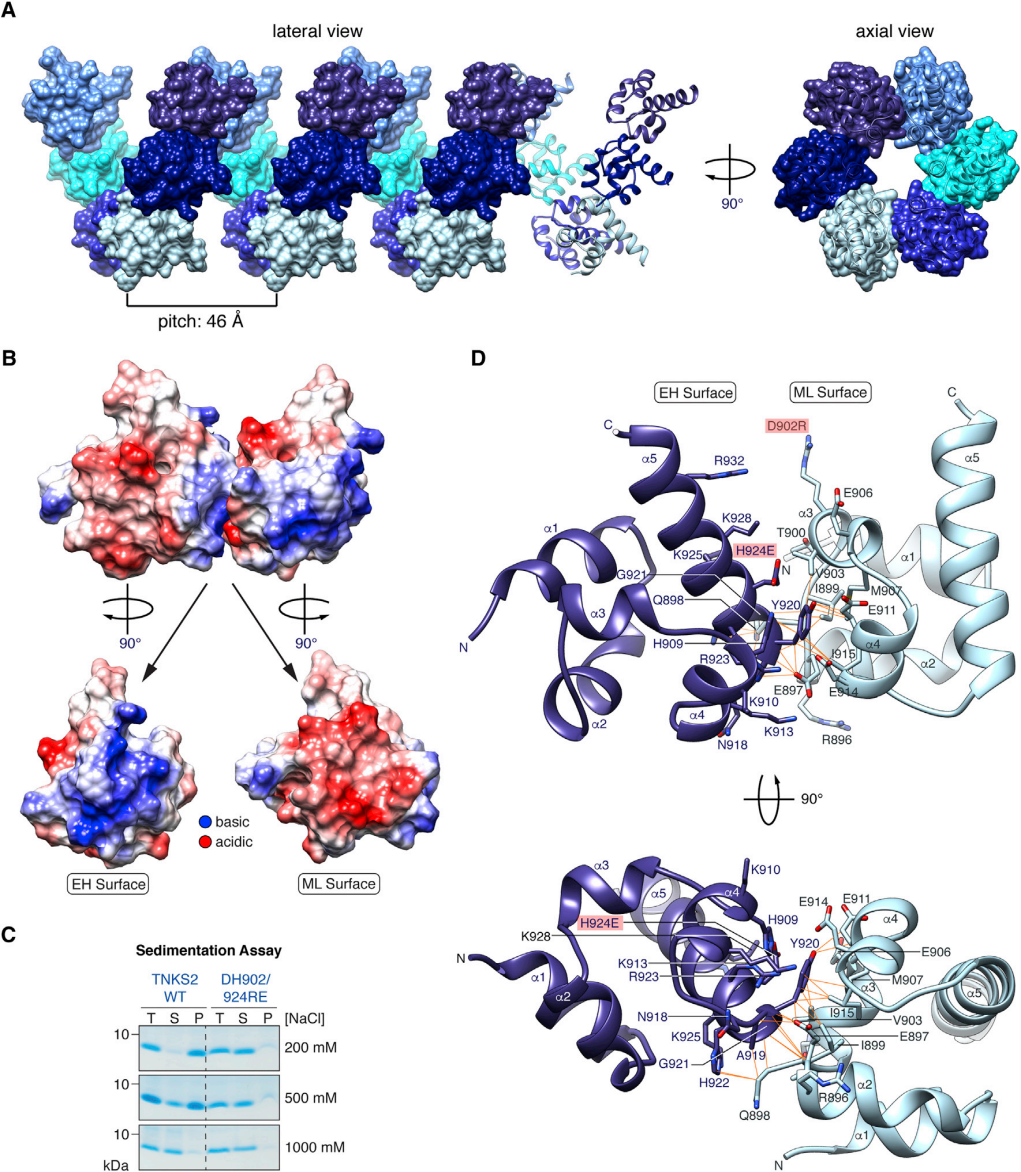
Crystal Structure of the TNKS2 SAM Domain (A) A structural representation of the TNKS2 DH902/924RE^T2^ SAM domain filament is shown. (B) A pair of WT-rendered TNKS2 SAM domains from the filament, colored by Coulombic surface electrostatic potential, is shown. (C) Ultracentrifugation sedimentation assay as for Figure 2A at increasing [NaCl] is shown. (D) Detailed representation of a TNKS2 DH902/924RE^T2^ SAM domain pair. Interface residues are in stick representation with orange lines indicating contacts. Mutations required for crystallization are indicated.

To enable the generation of well-defined Tankyrase mutants, we crystallized the TNKS2 SAM domain. Since polymerization was likely to hinder crystallization, we used the polymerization-impaired DH902/924RE^T2^ mutant. Reasoning that the mutant domain would still retain most polymerization interface residues, polymer contacts would be recoverable at the high protein concentration during crystallization, as illustrated for other polymerizing SAM domains (Kim et al., 2001, Kim et al., 2002). We obtained well-diffracting crystals (1.53 Å) and solved the structure by molecular replacement (Table 1; Supplemental Experimental Procedures). The TNKS2 SAM domain, a 5-α-helix bundle similar to other SAM domains, formed a left-handed helix with a pitch of 46 Å, whose axis coincided with the crystallographic P6_5_ screw axis (Figure 3A). The SAM domains interacted in the well-established end-helix (EH)-mid-loop (ML) binding mode (Qiao and Bowie, 2005) (Figures 3B and 3D). On the EH surface, basic electrostatic potential predominated while the ML surface was predominantly acidic, in line with the salt sensitivity of the polymer (Figures 3B and 3C). The closest approach between the two surfaces occurred around the N terminus of helix α5, where EH surface residues A919^T2^, Y920^T2^, G921^T2^, and H922^T2^ engaged in a network of hydrogen bonds and van der Waals contacts (Figure 3D). H922^T2^ and A919^T2^ contacted the Q898^T2^ side chain. Y920^T2^ was the most buried side chain at the interface (125 Å^2^), interacting with various hydrophobic ML side chains (V903^T2^, I899^T2^, I915^T2^, and M907^T2^), which collectively formed a shallow pocket, as well as E911^T2^ and E897^T2^. In turn, E897^T2^ bound the protein main chain at A919^T2^ and Y920^T2^. The main chains of adjacent SAM domains interacted between G921^T2^ and E897^T2^/Q898^T2^. The interface opened up toward the outside of the filament. In its non-mutated form, D902^T2^ likely forms a salt bridge with R932^T2^, which may promote another salt bridge between K928^T2^ and E906^T2^. Surprisingly, despite its importance for TNKS2 SAM domain polymerization, R896^T2^ was not involved in any contact (Figure 3D).

**Figure 4:**
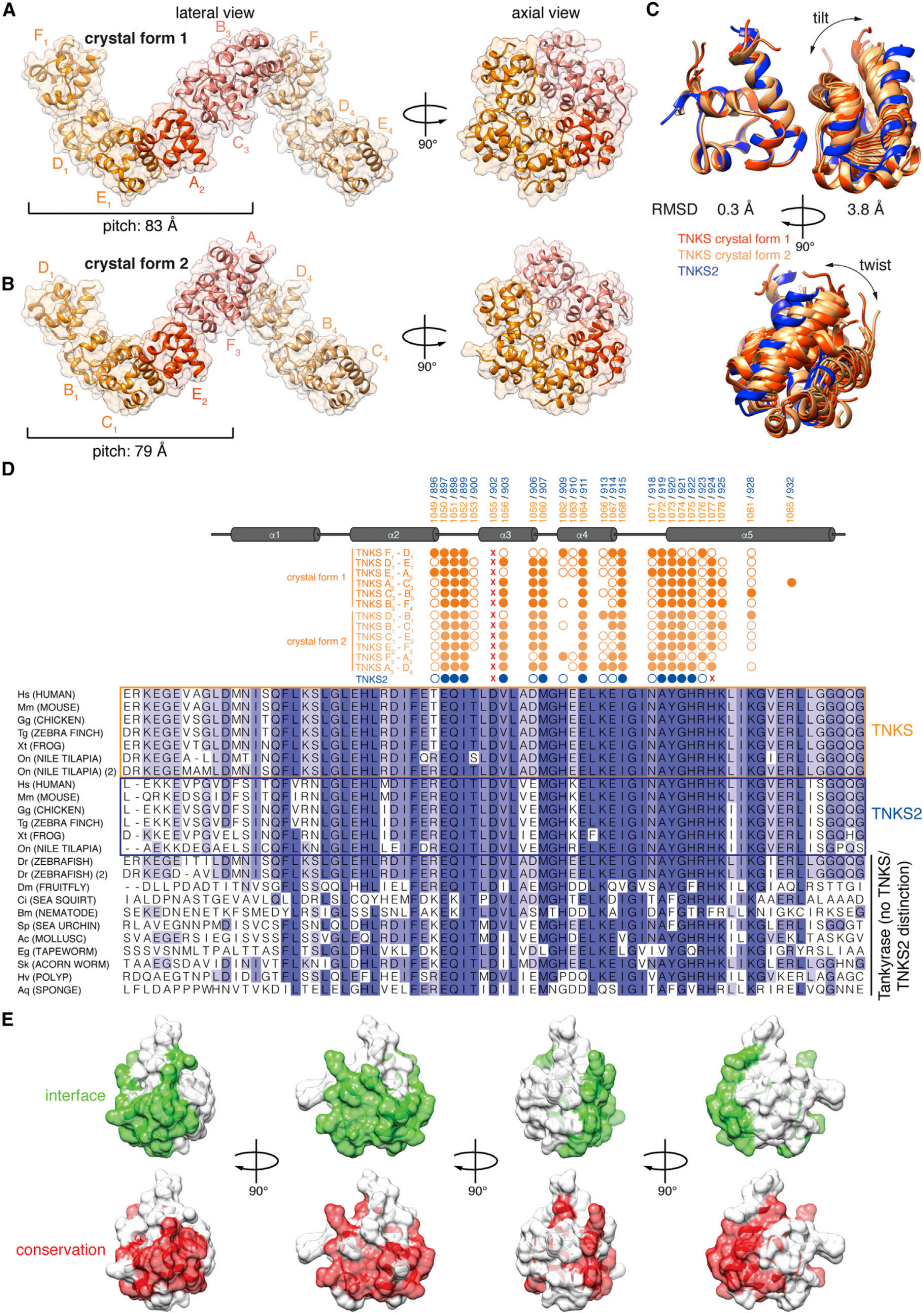
Crystal Structures of the TNKS SAM Domain and Comparison with TNKS2 (A and B) Structural representations of TNKS D1055R^T1^ SAM domain filaments are shown. Subscript numbers of chain identifiers denote the corresponding asymmetric units. See Figure S4 for a contact analysis. (C) EH-presenting SAM domains from unique SAM domain pairs were superimposed over residues 1,030–1,068^T1^/877–933^T2^, and average Cα root-mean-square deviation (RMSD) values for both protomers were calculated. (D) Multiple sequence alignment of SAM domains from representative Tankyrase orthologs. Circles denote interface residues (by solvent inaccessibility); filled circles indicate explicit contacts in crystal structures. X, mutated residues. See Supplemental Experimental Procedures for sequence accession numbers. (E) Conservation of the SAM-SAM interface. Top: interface residues observed in any of the crystal structures are in green, and bottom: residues identical in ≥80% of the orthologs shown in (D) are in red.

We also crystallized the TNKS SAM domain, which again required a polymer-breaking mutation. TNKS SAM D1055R^T1^, equivalent to D902R^T2^, produced two crystal forms in space group P2_1_, diffracting to 2.5 Å (crystal form 1) and 2.9 Å (crystal form 2), both with six molecules in distinct asymmetric units (Table 1; Figures 4A, 4B, and S4). The TNKS SAM domain was highly similar to that of TNKS2 (Figure 4C, left). For both TNKS crystal forms, non-crystallographic and crystallographic symmetry gave rise to left-handed helical filaments established by EH-ML contacts (Figures 4A, 4B, and S4). The repeating unit consisted of six SAM domains with pitches of 83 and 79 Å, almost twice as long as for TNKS2 (Figures 4A and 4B). Unlike for TNKS2, where protomer contacts relied on crystallographic symmetry only and were therefore uniform, the TNKS SAM EH-ML contacts varied substantially. This was apparent from the merely approximate 6-fold axial symmetry and the variable tilt and twist between adjacent SAM domains (Figures S4 and 4C). The three crystal structures provided snapshots of 13 unique SAM domain pairs. Many contacts were shared between all EH-ML interactions, but a subset was specific to certain binding geometries, sometimes involving the same residue in alternative interactions (Figures 4D and S4C). We conjecture that the variable relative orientations of SAM domains reflect filament flexibility (Figure 2B). SAM-SAM interface residues were conserved across a wide range of phyla, including poriferans, indicating that polymerization is a common and ancient feature of Tankyrase (Figures 4D and 4E).

### Characterization of Polymer Contacts by Mutagenesis

**Figure 5:**
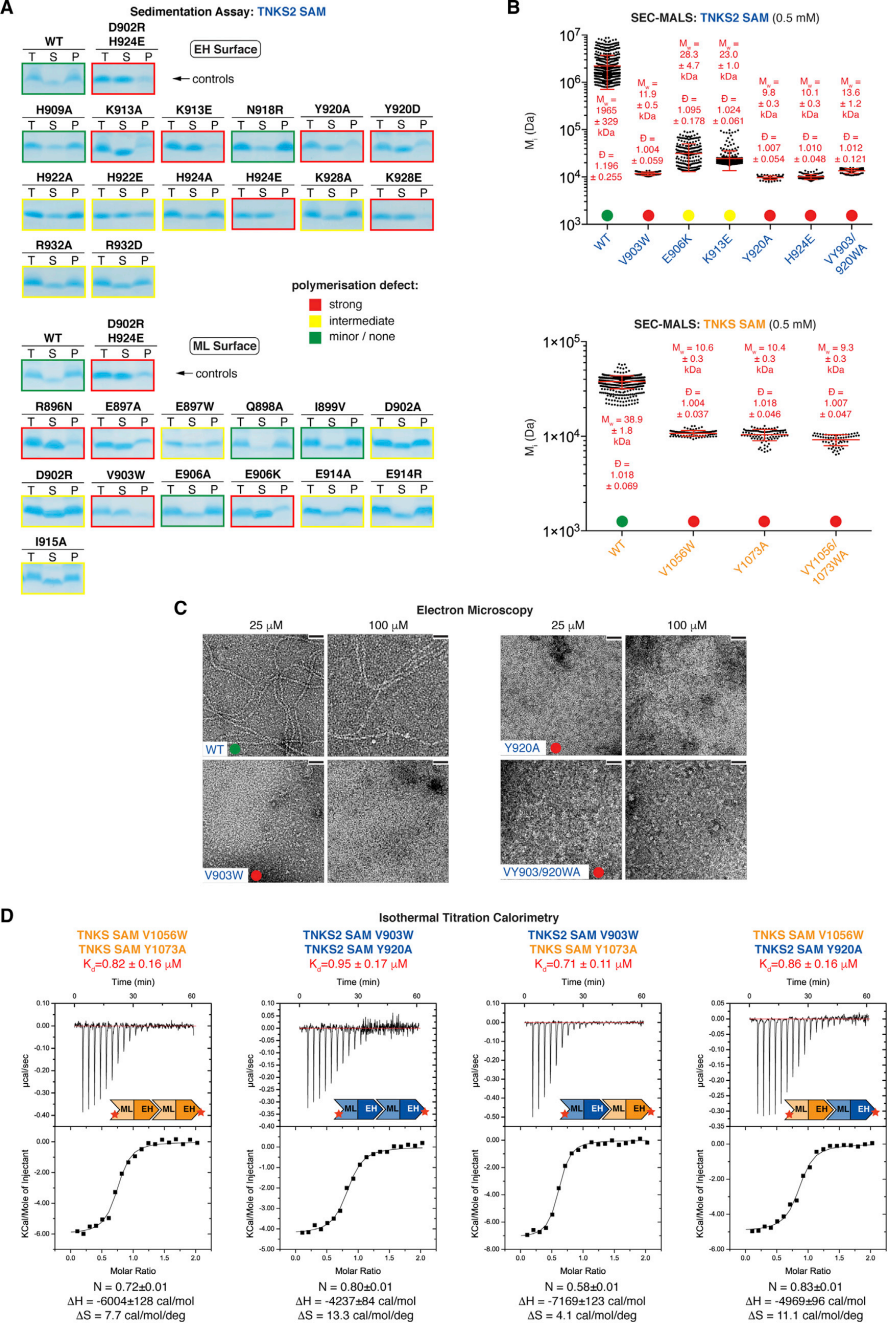
Characterization of Tankyrase SAM Domain Mutants (A) Ultracentrifugation sedimentation assays as for Figure 2A. Color coding indicates the degree of abrogated sedimentation. H924^T2^, K928^T2^, and E906^T2^ charge reversals were more severe than changes to alanine. (B) SEC-MALS of Tankyrase SAM domains, as in Figure 2C (M_w_ ± SD, Ð ± SD, n = 2). Color coding is as in (A). TNKS2 WT reference data, from the same experiment, are identical to Figure 2C. See Figure S3B for eluate analyses by SDS-PAGE and Figures S5A and S5B for CD spectroscopy. (C) EM of TNKS2 SAM domains. Color coding is as in (A). See Figure S3F for further mutants. Scale bars, 50 nm. (D) ITC analysis for the indicated SAM domain pairs. Mutated surfaces are indicated by the star in the schematics. See Figure S5C for a second experiment.

We performed site-directed mutagenesis of the TNKS2 SAM domain and assessed polymerization by ultracentrifugation sedimentation. Mutations strongly, intermediately, or weakly abrogated sedimentation (Figure 5A). In most cases, mutation of robust TNKS2 SAM contact residues (Y920^T2^, H924^T2^, E897^T2^, and V903^T2^) strongly impaired polymerization, as did mutation of E906^T2^, K913^T2^, and K928^T2^. Although situated close to the SAM-SAM interface, the latter three formed no explicit contacts in the TNKS2 SAM crystal structure (Figures 3D and 4D). However, the equivalent residues (D1059^T1^, K1066^T1^, and K1081^T1^, respectively) mediated binding between a subset of protomers in the TNKS SAM crystal structures (Figures 4D and S4C). Thus, contacts not seen in all SAM-SAM pairs are still generally relevant, probably occurring in some, but not all, configurations of the flexible filament.

We used SEC-MALS and EM to validate strong mutations (V903W^T2^, E906K^T2^, K913E^T2^, Y920A^T2^, H924E^T2^, and a VY903/920WA^T2^ combination). Except for E906K^T2^ and K913E^T2^, all mutations conferred monomeric behavior (Figures 5B and 5C). TNKS2 SAM K913E^T2^ and E906K^T2^ showed considerable residual polymerization (Figures 5B and S3F); we hence re-assigned their polymer-breaking scores to intermediate. As for TNKS2 SAM, the TNKS SAM mutations V1056W^T1^, Y1073A^T1^, and VY1056/1073WA^T1^ strongly abrogated polymerization (Figure 5B). Circular dichroism (CD) spectroscopy showed that the mutations did not impair SAM domain folding (Figures S5A and S5B).

Combining SAM domains with strong mutations in opposite polymerization surfaces (ML: V1056W^T1^ and V903W^T2^; EH: Y1073A^T1^ and Y920A^T2^) gave rise to homo- and heterotypic dimers (Figures S3D and S3E). This enabled us to assess the SAM-SAM binding affinities by isothermal titration calorimetry (ITC). TNKS and TNKS2 SAM domains bound homo- and heterotypically with comparable, low-micromolar affinities, typical for dynamic protein-protein interactions (Figures 5D and S5C; see Discussion).

### Full-Length Tankyrases Interact through EH and ML SAM Domain Surfaces

**Figure 6:**
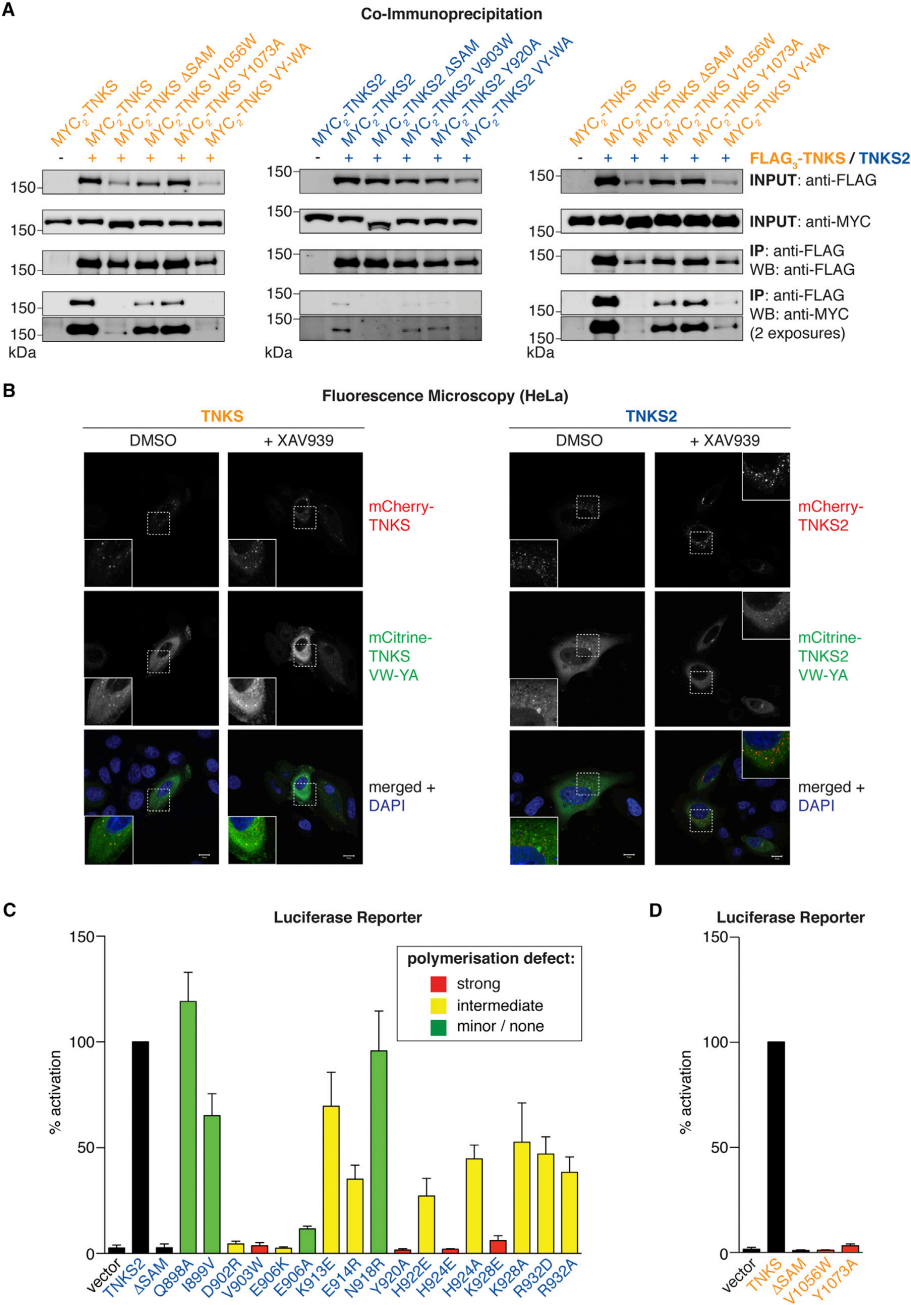
Tankyrase Requires Polymerization to Drive Wnt-β-Catenin Signaling (A) Homo- and heterotypic interactions of TNKS and TNKS2 in HEK293T cells. FLAG_3_-Tankyrases were immunoprecipitated, and co-precipitation of MYC_2_-Tankyrases was assessed by SDS-PAGE and western blotting. TNKS2 expression is lower than TNKS, accounting for the weaker apparent TNKS2 self-association (Figure S1E). See Figures S6A and S6B for cell lysate fractionations and additional co-immunoprecipitations. (B) Tankyrase polymerization controls localization. Serum-starved HeLa cells expressing the indicated mCherry- and mCitrine-tagged Tankyrases were vehicle or XAV939 treated. See Figures S6C and S6D for controls and additional experiments. Scale bar, 10 μm. (C and D) Tankyrase polymerization controls Wnt-β-catenin signaling. Transcription reporter assays for selected TNKS2 and TNKS SAM domain mutants, as for Figure 1C. Reporter activity was normalized to WT TNKS2 or TNKS (100%). Color coding reflects polymerization defects of the corresponding SAM domains as assessed by sedimentation, SEC-MALS, and EM (see Figure 5; n = 3 duplicate experiments; error bars, SEM). See Figure S1 for expression levels and Figure S7 for further data.

We assessed self-interaction of full-length Tankyrases in co-immunoprecipitations (co-IPs) with WT Tankyrases as bait. Robust homotypic binding of TNKS and TNKS2 was abolished by SAM domain deletion or mutation of both the ML and EH surfaces (VY1056/1073WA^T1^ and VY903/920WA^T2^), and it was reduced by mutation of either the ML surface (V1056W^T1^ and V903W^T2^) or EH surface (Y1073A^T1^ and Y920A^T2^) alone (Figure 6A, left and center). We also detected heterotypic binding of TNKS and TNKS2 and confirmed its sensitivity to SAM domain mutations (Figure 6A, right). The SAM domain previously was shown to confer high apparent molecular weight to TNKS in gel filtration experiments (De Rycker and Price, 2004). Using the VY1056/1073WA^T1^ and VY903/920WA^T2^ point mutants, we tested whether this reflects Tankyrase polymerization. WT TNKS and TNKS2 eluted close to the void volume with subsequent trails (Figure S6A). Both deletion and point mutation of the SAM domain resulted in an elution delay and increased trailing with an emerging late elution peak. We detected endogenous TNKS in both the early and late peaks, suggesting that TNKS exists in heterogeneous polymerization states (Figure S6A), but we were unable to detect endogenous TNKS2. Collectively, co-IP and gel filtration showed that full-length Tankyrases homo- and heteropolymerize. Using both assays, we found no evidence for modulated polymerization of full-length TNKS or TNKS2 by the T1049R^T1^ or R896T^T2^ mutations, suggesting that differential polymerization may not occur in a full-length context or only under particular conditions (Figures S6A and S6B).

### Polymerization Controls Tankyrase Subcellular Localization

To address if polymerization affects Tankyrase subcellular localization, we imaged HeLa cells expressing mCitrine- and mCherry-tagged TNKS or TNKS2. Since Tankyrase PARP activity was proposed to inhibit polymerization (De Rycker and Price, 2004), we compared vehicle- and XAV939-treated cells (Figure 6B). Both mCherry-TNKS and -TNKS2 displayed a punctate, predominantly cytoplasmic distribution, with more pronounced puncta upon XAV939 treatment (Figure 6B). In contrast, co-expressed mCitrine-tagged non-polymerizing EH/ML double mutants (VY1056/1073WA^T1^ and VY903/920WA^T2^) displayed mostly diffuse localization, even in the presence of XAV939 (Figure 6B; see Figure S6C for additional controls). This shows that polymerization enables the assembly of both TNKS and TNKS2 higher-order structures. In line with heteropolymerization, differentially tagged TNKS and TNKS2 colocalized (Figure S6D).

### Polymerization Is Required for Tankyrase-Dependent Wnt Signaling

We tested how SAM domain mutations affect the ability of TNKS2 to drive Wnt signaling. We observed a correlation between the severity of the polymerization defect and diminished transcription reporter activity (Figure 6C). Likewise, strong polymer-breaking mutations abolished Wnt signaling induced by TNKS (Figure 6D). Transcription reporter assays using paired TNKS2 mutants with inactivated opposite SAM domain faces suggested that Tankyrase dimerization is insufficient to drive Wnt signaling (Figure S7A). A heterologous polymerizing SAM domain, that of *D. melanogaster* Polyhomeotic (Kim et al., 2002), only partially compensated for SAM domain loss in TNKS2; however, the partial rescue was dependent on polymerization (Figures S7B–S7E). In conclusion, SAM domain polymerization enables Tankyrase function in Wnt-β-catenin signaling.

### Polymerization Promotes Tankyrase PARP Activity and Interaction with AXIN

**Figure 7:**
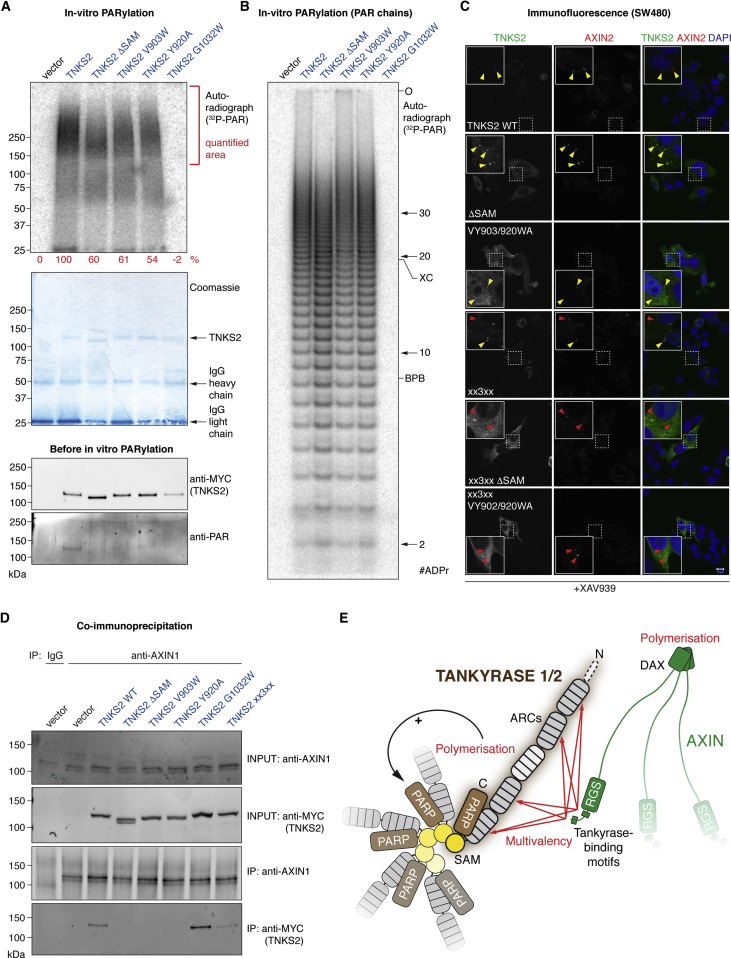
Tankyrase Polymerization Supports PARP Activity and Interaction with AXIN (A) In vitro PARylation by immunoprecipitated MYC_2_-TNKS2. Top: autoradiograph with quantitation is shown; middle: corresponding Coomassie-stained SDS-PAGE gel is shown, and bottom: western blot analysis of immunoprecipitates prior to in vitro PARylation is shown. (B) PAR was released from samples analyzed in (A) and equal amounts of PAR, or all available sample for vector and TNKS2 G1032W, analyzed by PAGE and autoradiography. Origin (O), the xylene cyanol (XC) and bromophenol blue (BPB) markers and PAR chain length are indicated. (C) SW480 cells expressing the indicated MYC_2_-tagged TNKS2 constructs were XAV939 treated, fixed, and stained for MYC_2_-TNKS2, endogenous AXIN2, and DNA. Yellow arrows denote degradasomes with AXIN2-TNKS2 colocalization; red arrows denote degradasomes containing AXIN2, but not TNKS2. Scale bar, 10 μm. (D) Endogenous AXIN1 was immunoprecipitated from HEK293T cells expressing the indicated MYC_2_-TNKS2 constructs. Samples were analyzed by SDS-PAGE and western blotting. (E) A model for the role of polymers and multivalency in the Tankyrase-AXIN system. See the Discussion for details. Red arrows, interactions; black arrow, regulation.

To explore the mechanism by which Tankyrase polymerization promotes Wnt signaling, we assessed the in vitro auto-PARylation activity of immunoprecipitated MYC_2_-TNKS2 WT, ΔSAM^T2^, V903W^T2^, Y920A^T2^, and the catalytically inactive variant G1032W^T2^. We readily observed TNKS2-dependent PARylation (Figure 7A). The ΔSAM^T2^, V903W^T2^, and Y920A^T2^ mutations reduced PARylation by ≈40%–50% and also accounted for strongly reduced endogenous PARylation, prior to the in vitro reaction (Figure 7A). Our observations agree with previous reports of reduced TNKS/TNKS2 activity upon SAM domain deletion (De Rycker and Price, 2004, Levaot et al., 2011), and they clarify that polymerization is required. To evaluate PARylation processivity, we detached PAR chains from the proteins and analyzed their size distribution. PAR from TNKS2 WT, V903W^T2^, and Y920A^T2^ showed similar lengths, indicating that polymerization does not affect auto-PARylation processivity (Figure 7B). Conversely, TNKS2 ΔSAM^T2^ produced overall shorter PAR chains (Figure 7B), suggesting that the SAM domain may impact PAR chain length independently of its polymerization.

We next asked whether Tankyrase polymerization promotes its interaction with AXIN. In colorectal cancer cells, but not HeLa cells with their intact Wnt-β-catenin pathway, Tankyrase and AXIN1/2 have been shown to colocalize in β-catenin degradasomes induced by Tankyrase inhibitors (de la Roche et al., 2014, Martino-Echarri et al., 2016, Thorvaldsen et al., 2015). We hence analyzed SW480 colorectal cancer cells and observed that transiently expressed MYC_2_-TNKS2 and endogenous AXIN2 accumulate in puncta upon XAV939 treatment (Figure 7C). Provided AXIN2 levels were sufficient for immunodetection, TNKS2 colocalized with AXIN2 in degradasomes (Figure 7C). Deletion or mutation of the SAM domain (ΔSAM^T2^ and VY903/920WA^T2^) resulted in a more diffuse TNKS2 localization; however, we still detected substantial colocalization of these mutants with AXIN2 puncta, likely due to the interaction of the ARCs with AXIN at overexpression levels of Tankyrase. Inactivation of the ARCs (xx3xx) did not abolish puncta formation by TNKS2 but substantially reduced its colocalization with AXIN2 foci (Figure 7C). The retained colocalization may reflect residual AXIN2 binding by the xx3xx mutant and/or additional determinants, including bridging through endogenous Tankyrase. When combined with the xx3xx mutations, the ΔSAM^T2^ or VY903/920WA^T2^ mutations resulted in diffuse TNKS2 staining without colocalization in AXIN2 puncta (Figure 7C). Thus, polymerization contributes to the recruitment of TNKS2 to β-catenin degradasomes.

To more directly evaluate if Tankyrase polymerization promotes AXIN binding, we immunoprecipitated endogenous AXIN1 from HEK293T cells (avoiding AXIN overexpression to maintain limiting levels), and we assessed its binding to MYC_2_-TNKS2 (Figure 7D). AXIN1 robustly bound to TNKS2 and its catalytically inactive mutant G1032W^T2^. However, recovery of TNKS2 ΔSAM^T2^, V903W^T2^, Y920A^T2^, and the xx3xx mutant was strongly reduced (Figure 7D). Taken together, the microscopy and binding studies illustrate that SAM domain-mediated polymerization promotes Tankyrase interaction with AXIN in β-catenin degradasomes.

## Discussion

We propose a model in which multivalency, mediated by two Tankyrase-binding motifs in AXIN (Morrone et al., 2012) and four AXIN-binding ARCs in Tankyrase (Guettler et al., 2011), combined with polymerization of both proteins, gives rise to avidity for efficient Tankyrase recruitment to DCs (Figure 7E). Additionally, Tankyrase polymerization supports auto-PARylation and is expected to promote recruitment and activity of the E3 ubiquitin ligase RNF146, which also binds the ARCs (DaRosa et al., 2015). Our observation that Tankyrase-mediated scaffolding can drive Wnt-β-catenin signaling independently of catalytic PARP activity has important implications for the use of Tankyrase inhibitors to oppose oncogenic Wnt signaling.

The SAM-SAM contacts seen in our crystal structures are relevant to the full-length proteins. First, the SAM domains present their termini toward the filament periphery, compatible with protruding ARCs and PARP domains (Knight et al., 2011). Second, Tankyrase polymerization and its ability to activate β-catenin-dependent transcription correlate. Mutagenesis suggests that activation may require a TNKS- and TNKS2-specific polymerization threshold to be surpassed. Third, in co-IP, gel filtration, and light microscopy, full-length Tankyrases respond to mutation of the identified head-to-tail interfaces, in line with previous deletion studies (De Rycker and Price, 2004, Hatsugai et al., 2010). Tankyrase polymers display a punctate localization, as observed for other polymerizers, such as AXIN and Dishevelled in Wnt signaling (Fiedler et al., 2011), Polyhomeotic orthologs in transcriptional repression (Isono et al., 2013), and proteins of supramolecular organizing centers (SMOCs) in innate immune signaling (Kagan et al., 2014, Sherman et al., 2013). Puncta also were observed for endogenous Tankyrase in XAV939-treated colorectal cancer cells (de la Roche et al., 2014). Correlative light and EM showed that β-catenin DCs are of a filamentous sub-organization (Thorvaldsen et al., 2015). That these filaments do not grow to substantial length in cells likely reflects their dynamic nature (Bienz, 2014), a view compatible with micromolar SAM-SAM affinities and nanomolar Tankyrase concentrations in cells (Hein et al., 2015).

Compared to the TNKS2 SAM domain, that of TNKS polymerizes less efficiently. The higher molecular weight reported for chicken Tnks SAM polymers (De Rycker and Price, 2004) is based on elution volume, rather than static light scattering, and likely affected by the globular affinity tag and long flexible termini in the construct. Chicken MBP-Tnks SAM filaments are thus likely to be of similar length to the human TNKS SAM filaments analyzed here. R896^T2^, responsible for differential polymerization of isolated TNKS and TNKS2 SAM domains, or a basic residue is conserved across TNKS2 orthologs and Tankyrases from species lacking TNKS2 (Figure 4D). However, its role remains unclear. First, our crystal structures do not reveal how R896^T2^ contributes to polymerization. Although all crystal structures of polymerizing SAM domains to date support the EH-ML interaction mode (Harada et al., 2008, Kim et al., 2001, Kim et al., 2002, Leettola et al., 2014, Nanyes et al., 2014, Stafford et al., 2011), crystallization may impose constraints onto some aspects of filament architecture and conceal the role of R896^T2^. Second, TNKS and TNKS2 SAM domain affinities are similar by ITC, suggesting that the differences only become apparent in the context of WT filaments rather than pairs of mutant SAM domains. Third, the TNKS and TNKS2 SAM domains are mutually interchangeable for Wnt signaling, and the interconverting T1049R^T1^ and R896T^T2^ mutations do not appear to affect polymerization of the full-length proteins. Thus, differential polymerization may not occur in full-length Tankyrases or requires a yet unknown regulatory event. Given their heteropolymerization, the TNKS and TNKS2 pools may in fact not be separable.

Surprisingly, Tankyrase can induce β-catenin-dependent transcription independently of its catalytic PARP activity. The underlying mechanism relies on ARC- and SAM domain-dependent scaffolding but remains incompletely understood. Inactive Tankyrase may have a direct role in establishing β-catenin degradasomes (Martino-Echarri et al., 2016). TNKS or TNKS2 overexpression, either at the mRNA or protein level, has been described in numerous malignancies, including gastric cancer (Gao et al., 2011, Matsutani et al., 2001), breast cancer (Gelmini et al., 2004), bladder cancer (Gelmini et al., 2007), astrocytoma (Tang et al., 2012), glioblastoma (Shervington et al., 2007), pancreatic cancer (Zhao et al., 2009), lung cancer (Busch et al., 2013), and colon cancer (Gelmini et al., 2006, Shebzukhov et al., 2008). Polymerization and thus catalysis-independent Tankyrase functions may prevail when Tankyrase is overexpressed. Therefore, the effectiveness of catalytic Tankyrase inhibitors may be limited when Tankyrase levels are high (see Figure 1D). Likewise, Tankyrase inhibitors stabilize Tankyrases through the blockage of PARdU (Huang et al., 2009), which may exacerbate polymerization. Blockage of scaffolding provides an additional promising avenue for pharmacologic inhibition of Tankyrase function.

Roles of polymeric Tankyrase likely extend beyond Wnt signaling, given the high prevalence of Tankyrase-binding proteins (Guettler et al., 2011). In analogy to SAM domain-containing transcriptional regulators (Isono et al., 2013), Tankyrase polymerization may facilitate protein regulation over an extensive physical range. Two such examples may be telomeres (Hsiao and Smith, 2008) and DNA repair sites (Nagy et al., 2016). Conversely, polymerization may suppress Tankyrase function in some cellular contexts. This study provides the tools to explore these questions.

## Experimental Procedures

The Supplemental Experimental Procedures are available in the Supplemental Information online.

### Luciferase Reporters

HEK293T cells were transfected in technical triplicate with TOPFlash or FOPFlash reporter plasmids (Veeman et al., 2003), a reference Renilla luciferase reporter, and the indicated Tankyrase or AXIN constructs. One replicate was analyzed for protein expression. Cells were maintained in low serum (DMEM with 0.3% fetal bovine serum [FBS]) following transfection or treated with XAV939. Luciferase activities were measured 24 hr after transfection complex addition and Renilla luciferase activity used for normalization. Data were analyzed as detailed in the figure legends.

### Protein Expression and Purification

SAM domains of human TNKS (1,018–1,093) and TNKS2 (867–940) were expressed in *E. coli* as His_6_-MBP-Asn_10_ fusion proteins, and they were purified by Ni affinity purification, tag removal, anion exchange, and SEC. Proteins were dialyzed into buffer with 200 mM NaCl prior to experiments. Proteins shown in Figures 3C and 5A were affinity purified.

### Crystallization, Structure Determination, and Analysis

Crystals of TNKS2 SAM DH902/924RE^T2^ and TNKS SAM D1055R^T1^ were grown and analyzed as detailed in the Supplemental Experimental Procedures. Crystal structures were determined by molecular replacement (Table 1). Interface residues were calculated using PISA (Proteins, Interfaces, Structures and Assemblies) (Krissinel and Henrick, 2007); contacts were analyzed and structural representations were generated using UCSF Chimera (Pettersen et al., 2004).

### Ultracentrifugation Sedimentation

SAM domains were centrifuged at an average speed of 200,000 × *g* at 20°C for 1 hr. Total, supernatant, and pellet samples were analyzed by SDS-PAGE and Coomassie staining.

### EM

SAM domains were applied to glow-discharged carbon-coated grids, negatively stained with 2% (w/v) uranyl acetate, and imaged on an FEI Tecnai 12 electron microscope.

### SEC-MALS

Proteins were resolved by size exclusion in a buffer with 200 mM NaCl. In-line light scattering was measured using a DAWN Heleos-II (Wyatt) and refractive index using an Optilab rEX (Wyatt). Overall weight-average molecular weight (M_w_) and dispersity (Ð) were calculated from two separate experiments analyzed in ASTRA (Wyatt).

### ITC

All proteins were dialysed into binding buffer with 200 mM NaCl. TNKS2 SAM Y920A^T2^ or TNKS SAM Y1073A^T1^ (500 μM) was injected in 2-μl increments into TNKS2 SAM V903W^T2^ or TNKS SAM V1056W^T1^ (50 μM) or buffer, using an ITC200 MicroCalorimeter (MicroCal/GE Healthcare). Data were processed using Origin7 (MicroCal/GE Healthcare) using a one-site binding model.

### In Vitro PARylation

MYC_2_-TNKS2 and derivatives were expressed in HEK293T cells and immunoprecipitated. PARP activity assays were performed with 1 mM NAD^+^ and 5 μCi ^32^P-NAD^+^ for 30 min at 30°C. PAR chains were detached and analyzed essentially as described previously (Alvarez-Gonzalez and Jacobson, 1987, Panzeter and Althaus, 1990). Immunoprecipitates and in vitro reactions were analyzed by western blotting and autoradiography, respectively.

### Co-IPs

HEK293T cells were transfected with the indicated Tankyrase or control constructs. For Figure 7D, cells were serum starved to match luciferase assays. Immunoprecipitates with anti-AXIN1 (C76H11 clone, Cell Signaling Technologies) or control IgG (sc-2027, Santa Cruz Biotechnology) were captured on Protein A/G magnetic resin (Thermo Scientific/Pierce). For Figures 6A and S6B, IPs were performed with anti-FLAG M2 affinity gel (Sigma). Lysates and immunoprecipitates were analyzed by SDS-PAGE and western blotting.

### Fluorescence Microscopy

HeLa or SW480 cells were transiently transfected with the indicated Tankyrase constructs. Cells in DMEM containing 0.3% FBS were treated either with DMSO vehicle or 2 μM XAV939 for 20 hr directly after transfection. Cells were fixed by the addition of 4% formaldehyde. Cells were immuno- and DAPI-stained as indicated.

## Author Contributions

C.M.T., L.M., P.P., M.R., and S.G. generated DNA constructs. L.M. and C.M.T. purified proteins. L.M. crystallized proteins, with N.C. collected diffraction data, and with N.C. and S.G. determined crystal structures. L.M. and C.M.T. performed ultracentrifugation assays. L.M. performed SEC-MALS, CD, and ITC. L.M. and F.B. performed EM and with E.M. analyzed the data. C.M.T. and S.G. performed luciferase reporters. M.R. performed PARP activity assays. L.M. and M.R. performed co-immunoprecipitations. M.R. and S.G. performed lysate fractionations and fluorescence microscopy. P.P. contributed to protein purification, ultracentrifugation, and EM. S.G. designed the study together with the other authors and supervised the research. S.G. wrote the manuscript with input from all authors.

## Figures and Tables

**Table 1:** Data Collection and Refinement Statistics

Data Collection^a^	TNKS2 SAM DH902/924RE	TNKS SAM D1055R Crystal Form 1 (Five Datasets/Three Crystals)	TNKS SAM D1055R Crystal Form 2 (Two Datasets/Two Crystals)
PDB ID	5JRT	5JU5	5JTI
Beamline	Diamond I03	Diamond I03	Diamond I03
Wavelength (Å)	0.976	0.976	0.976
Space group	P6_5_	P2_1_	P2_1_
Unit cell			
a, b, c (Å)	56.63, 56.63, 46.11	52.24, 55.22, 83.05	70.93, 55.48, 79.41
α, β, γ (°)	90, 90, 120	90, 96.2, 90	90, 102.9, 90
Molecules/ASU	1	6	6
Resolution (Å)	28.32–1.53 (1.56–1.53)	82.57–2.5(2.6–2.5)	77.41–2.9 (3.0–2.9)
Total number of reflections	207,561 (10,406)	454,569 (51,435)	87,050 (13,527)
Number of unique reflections	12,797 (618)	16,511 (1,870)	13,604 (2,185)
R_merge_^b^	0.058 (2.932)	0.406 (5.731)	2.297 (6.557)
R_meas_^b^	0.061 (3.120)	0.422 (6.002)	2.525 (7.589)
Mean I/σI	20.5 (0.9)	11.2 (1.3)	9.6 (1.4)
CC_1/2_^c^	0.999 (0.408)	0.997 (0.333)	0.892 (0.35)
CC:d1		0.996 (0.030)	0.969 (0.494)
CC:d2		0.998 (0.713)	0.817 (0.256)
CC:d12	0.99 (0.323)		
CC:d3	0.99 (0.480)	0.999 (0.683)	0.973 (0.678)
Completeness (%)	100 (100)	100 (100)	100 (100)
Multiplicity	16.2 (16.7)	27.5 (27.5)	6.6 (6.5)
Wilson B factor (Å)^b^	37.69	34.51	18.57

**Refinement**^a^			

Resolution (Å)	28.32–1.53	82.57–2.5	77.41–2.9
R_work_/R_free_ (test set 5%)	0.201/0.233	0.191/0.211	0.193/0.232
Reflections used in refinement	12,770	16,498	13,593
Reflections in R_free_ test set	636	802	681
RMSD bond lengths (Å)	0.01	0.01	0.01
RMSD bond angles (°)	0.94	1.13	1.19
Number of protein atoms	510	2,855	2,854
Number of solvent atoms	40	24	62
B factor protein (Å)^b^	46.71	65.21	53.1
B factor solvent (Å)^b^	50.92	52.81	36.54
Ramachandran favored (%)	100	99	96.5
Ramachandran allowed (%)	0	1	3.5
Ramachandran disallowed (%)	0	0	0

a Values for the highest-resolution shell are shown in parentheses.

b R_merge_ and R_meas_ are as calculated in AIMLESS (Winn et al., 2011). High R_merge_ and R_meas_ are attributable to the high-resolution cutoff (Karplus and Diederichs, 2012), multi-dataset merging, and anisotropy (see next footnote).

c The principal directions of anisotropy are as defined by symmetry (axes or planes), as analyzed in AIMLESS (Winn et al., 2011). For TNKS SAM crystal form 1, the anisotropy is pronounced along CC_d1 (along 0.91 hr–0.40 l), with CC_1/2_ falling below 0.30 at 3.0 Å.

## Accession Numbers

The accession numbers for the structure coordinates and experimental structure factors reported in this paper are PDB: 5JRT, 5JU5, and 5JTI.

## Note Added in Proof

While this study was in press, DaRosa et al. reported a model for the TNKS SAM domain polymer, supported by biophysical evidence. The findings are in agreement with this study.

DaRosa, P.A., Ovchinnikov, S., Xu, W., and Klevit, R.E. (2016). Structural insights into SAM domain-mediated tankyrase oligomerization. Protein Sci. Published online June 21, 2016. http://dx.doi.org/10.1002/pro.2968.

## Supplemental Information

Document S1. Supplemental Experimental Procedures, Figures S1–S7, and Table S1

Document S2. Article plus Supplemental Information
