# Supplementary material for: Tankyrase Requires SAM Domain-Dependent Polymerization to Support Wnt-β-Catenin Signaling
Source: Mol Cell. 2016 Aug 4;63(3):498–513. doi: 10.1016/j.molcel.2016.06.019 (PMC4980433; doi:10.1016/j.molcel.2016.06.019)
Supplement: Document S1. Supplemental Experimental Procedures, Figures S1–S7, and Table S1 [file mmc1.pdf]

Molecular Cell, Volume *63*

Supplemental Information

Tankyrase Requires SAM Domain-Dependent Polymerization to Support Wnt-β-Catenin Signaling

Laura Mariotti, Catherine M. Templeton, Michael Ranes, Patricia Paracuellos, Nora Cronin, Fabienne Beuron, Edward Morris, and Sebastian Guettler

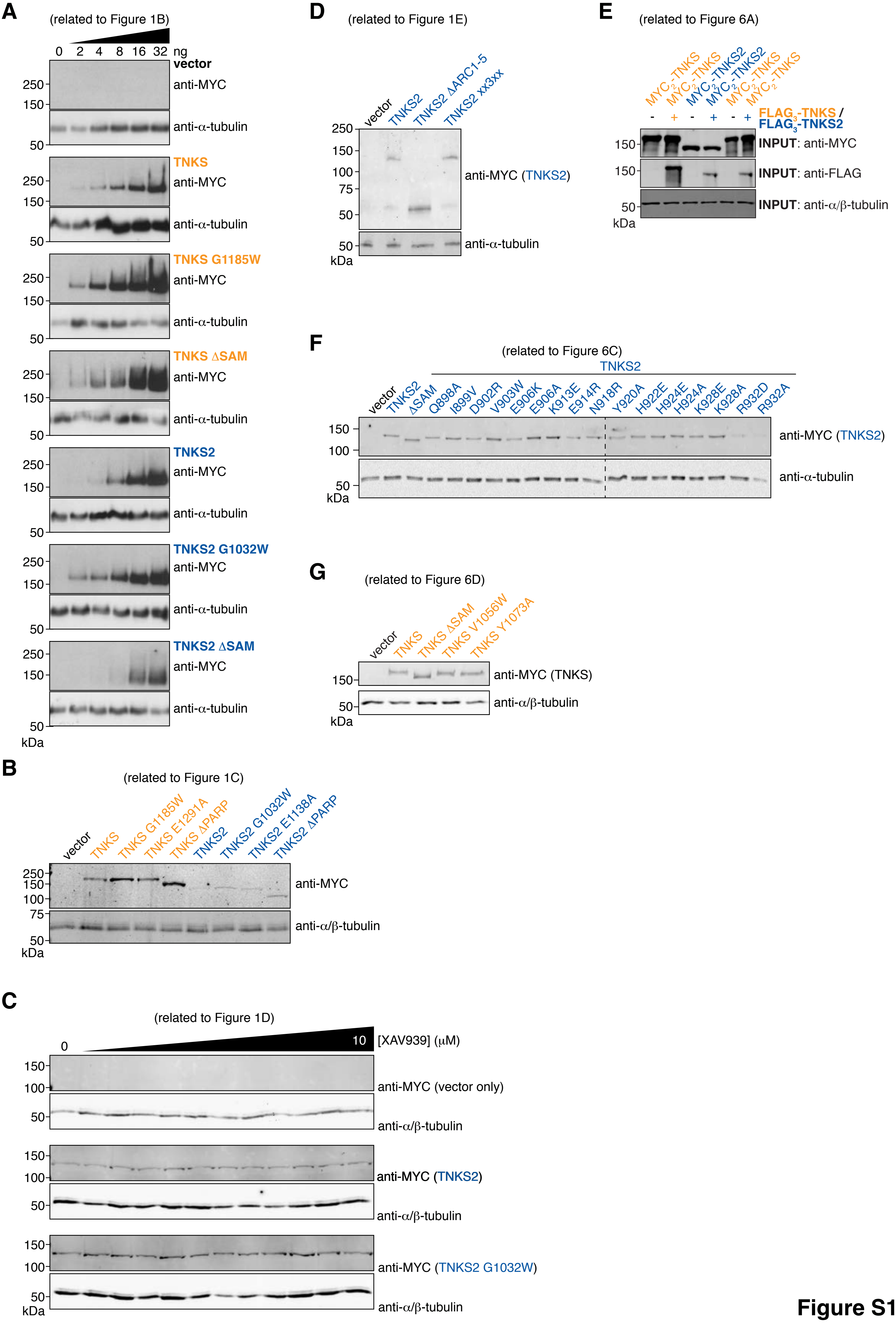
A
(related to Figure 1B)
0 2 4 8 16 32 ng
vector
TNKS
TNKS G1185W
TNKS ΔSAM
TNKS2
TNKS2 G1032W
TNKS2 ΔSAM
anti-MYC
anti-α-tubulin
250
150
50
kDa
B
(related to Figure 1C)
vector
TNKS
TNKS G1185W
TNKS E1291A
TNKS ΔPARP
TNKS2
TNKS2 G1032W
TNKS2 E1138A
TNKS2 ΔPARP
anti-MYC
anti-α/β-tubulin
250
150
100
75
50
kDa
C
(related to Figure 1D)
0
10
[XAV939] (μM)
anti-MYC (vector only)
anti-α/β-tubulin
anti-MYC (TNKS2)
anti-MYC (TNKS2 G1032W)
150
100
50
kDa
D
(related to Figure 1E)
vector
TNKS2
TNKS2 ΔARC1-5
TNKS2 xx3xx
anti-MYC (TNKS2)
anti-α-tubulin
250
150
100
75
50
kDa
E
(related to Figure 6A)
MYC2-TNKS
MYC2-TNKS
MYC2-TNKS2
MYC2-TNKS2
MYC2-TNKS
MYC2-TNKS
- + - + - +
FLAG3-TNKS /
FLAG3-TNKS2
INPUT: anti-MYC
INPUT: anti-FLAG
INPUT: anti-α/β-tubulin
150
50
kDa
F
(related to Figure 6C)
TNKS2
vector
TNKS2
ΔSAM
Q898A
I899V
D902R
V903W
E906K
E906A
K913E
E914R
N918R
Y920A
H922E
H924E
H924A
K928E
K928A
R932D
R932A
anti-MYC (TNKS2)
anti-α-tubulin
150
100
50
kDa
G
(related to Figure 6D)
vector
TNKS
TNKS ΔSAM
TNKS V1056W
TNKS Y1073A
anti-MYC (TNKS)
anti-α/β-tubulin
150
50
kDa

Figure S1

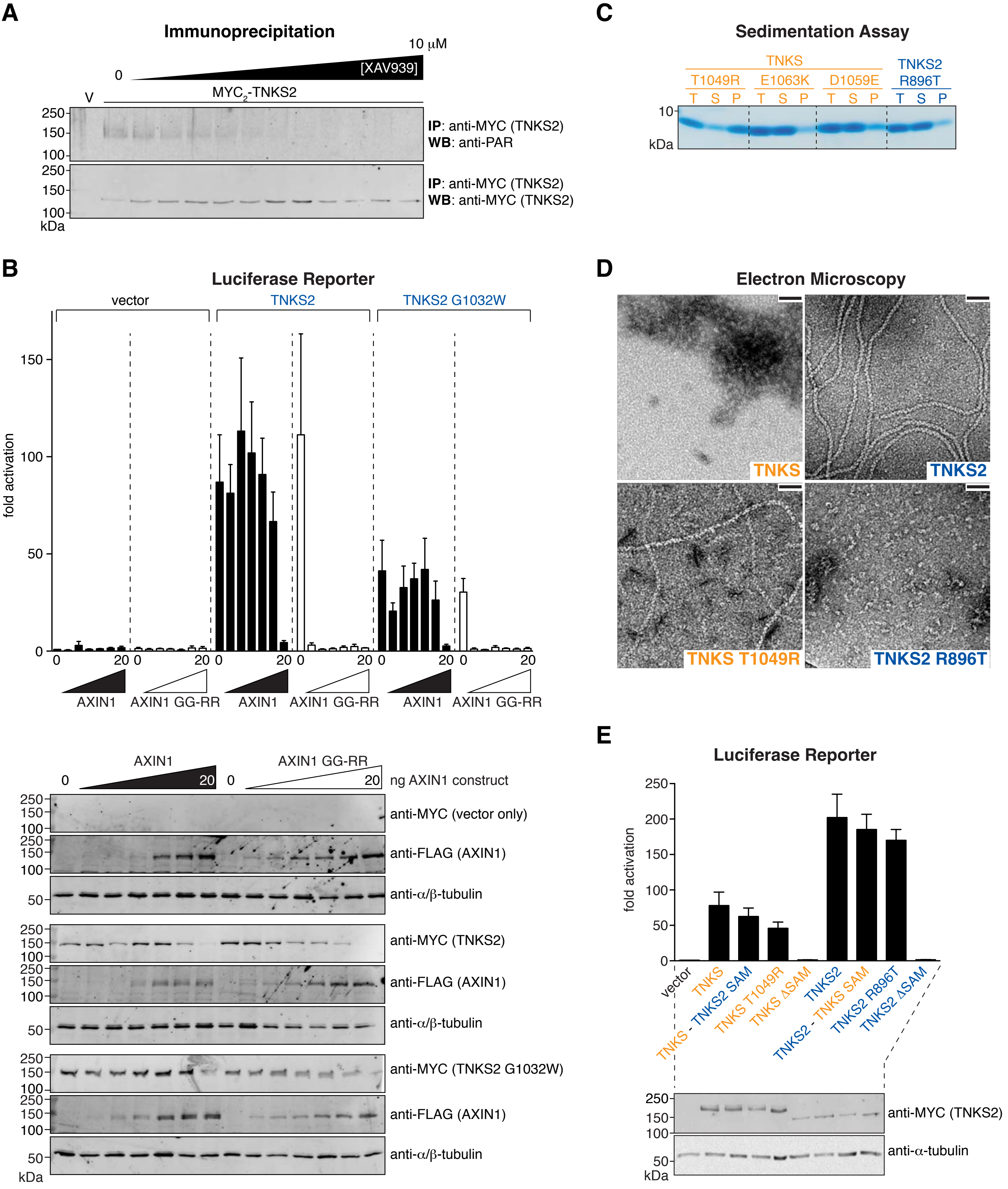

A
Immunoprecipitation
0
10 μM
[XAV939]
V
MYC2-TNKS2
IP: anti-MYC (TNKS2)
WB: anti-PAR
IP: anti-MYC (TNKS2)
WB: anti-MYC (TNKS2)
kDa
B
Luciferase Reporter
vector
TNKS2
TNKS2 G1032W
fold activation
AXIN1
AXIN1 GG-RR
ng AXIN1 construct
anti-MYC (vector only)
anti-FLAG (AXIN1)
anti-α/β-tubulin
anti-MYC (TNKS2)
anti-MYC (TNKS2 G1032W)
C
Sedimentation Assay
TNKS
T1049R
E1063K
D1059E
TNKS2
R896T
T S P
D
Electron Microscopy
TNKS
TNKS2
TNKS T1049R
TNKS2 R896T
E
Luciferase Reporter
fold activation
vector
TNKS
TNKS - TNKS2 SAM
TNKS T1049R
TNKS ΔSAM
TNKS2
TNKS2 - TNKS SAM
TNKS2 R896T
TNKS2 ΔSAM
anti-MYC (TNKS2)
anti-α-tubulin

Figure S2

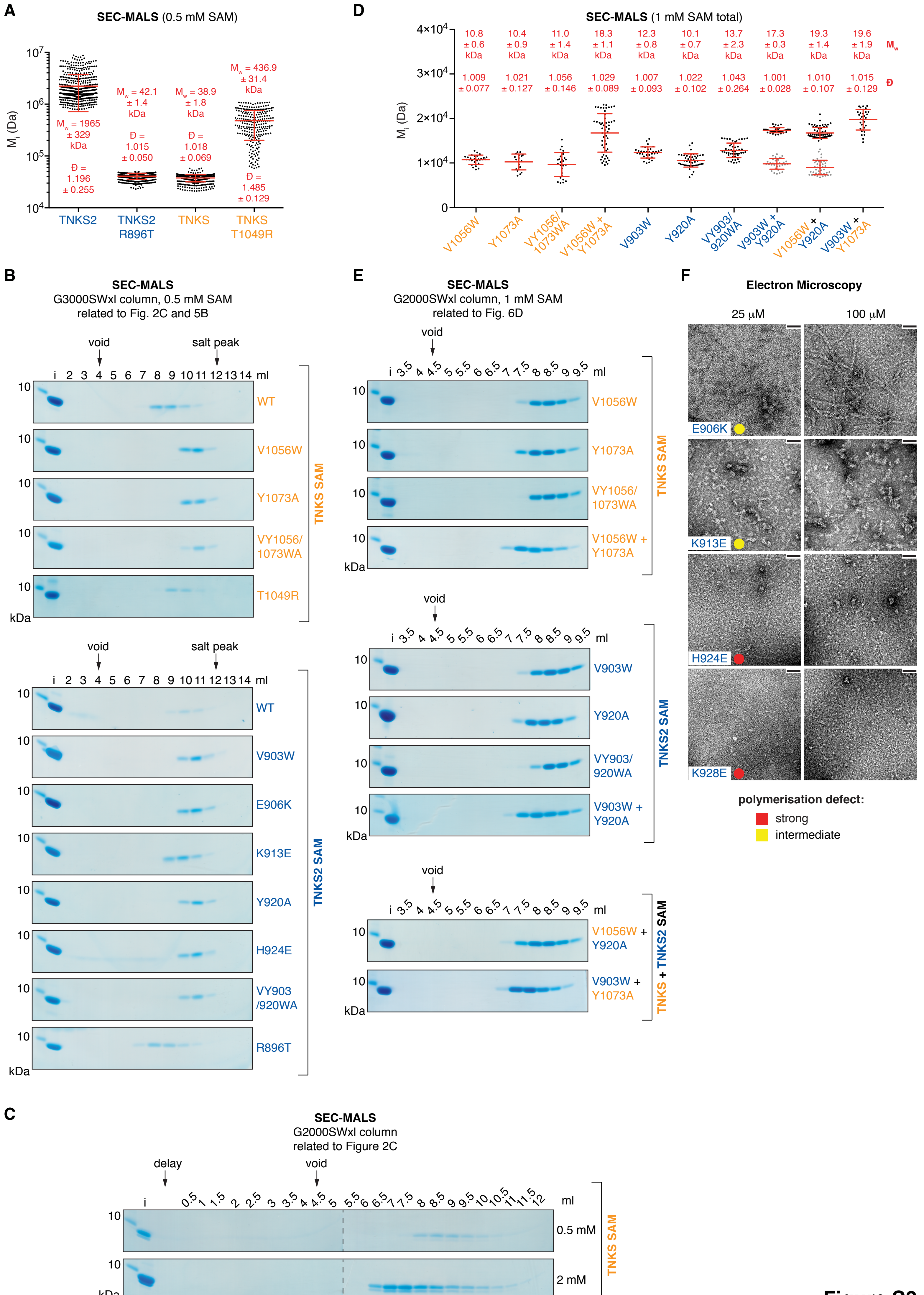
A
SEC-MALS (0.5 mM SAM)
TNKS2
TNKS2 R896T
TNKS
TNKS T1049R
B
SEC-MALS
G3000SWxl column, 0.5 mM SAM
related to Fig. 2C and 5B
TNKS SAM
TNKS2 SAM
C
SEC-MALS
G2000SWxl column
related to Figure 2C
D
SEC-MALS (1 mM SAM total)
E
SEC-MALS
G2000SWxl column, 1 mM SAM
related to Fig. 6D
F
Electron Microscopy
polymerisation defect:
strong
intermediate

Figure S3

A

B

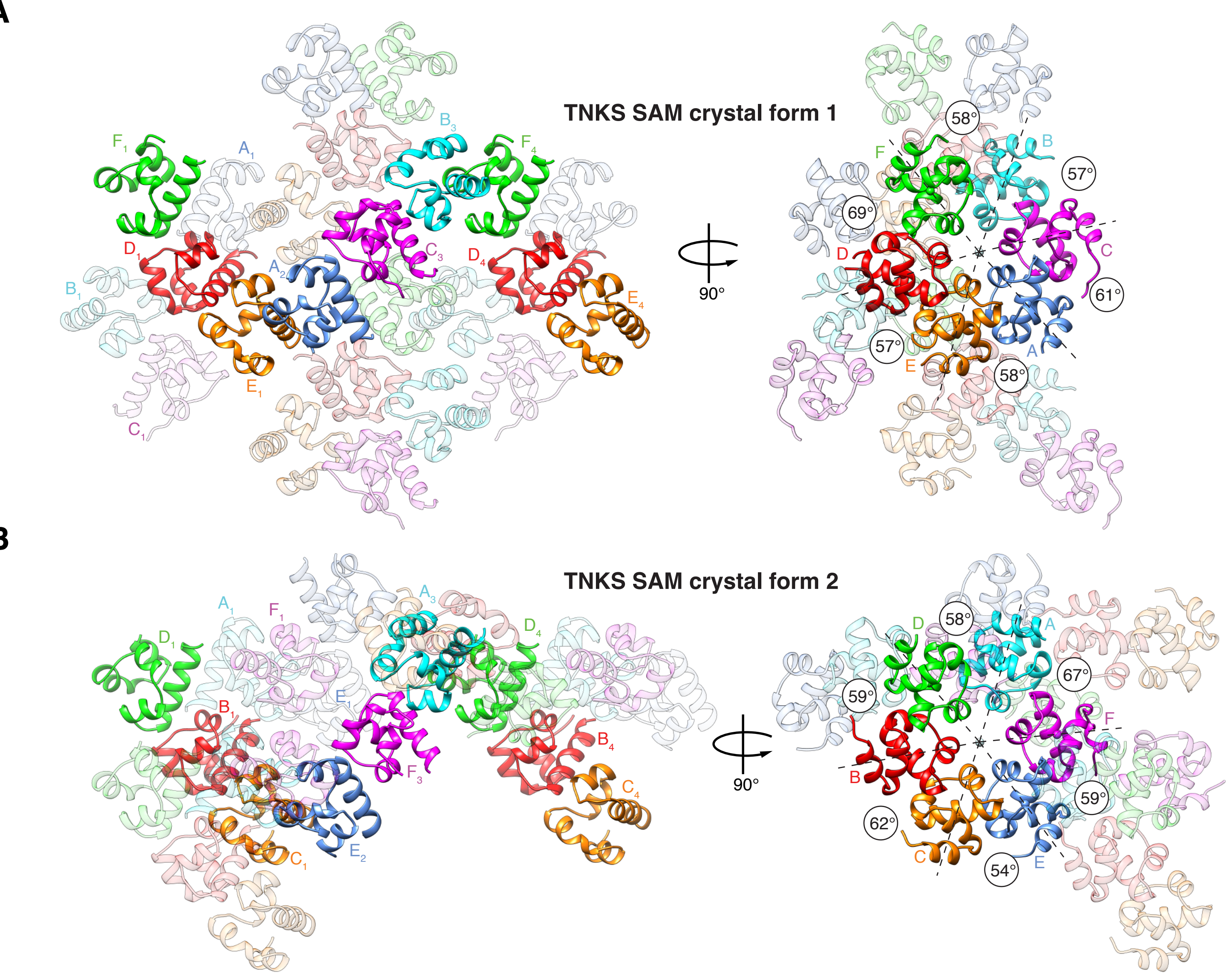

C

Contact Analysis

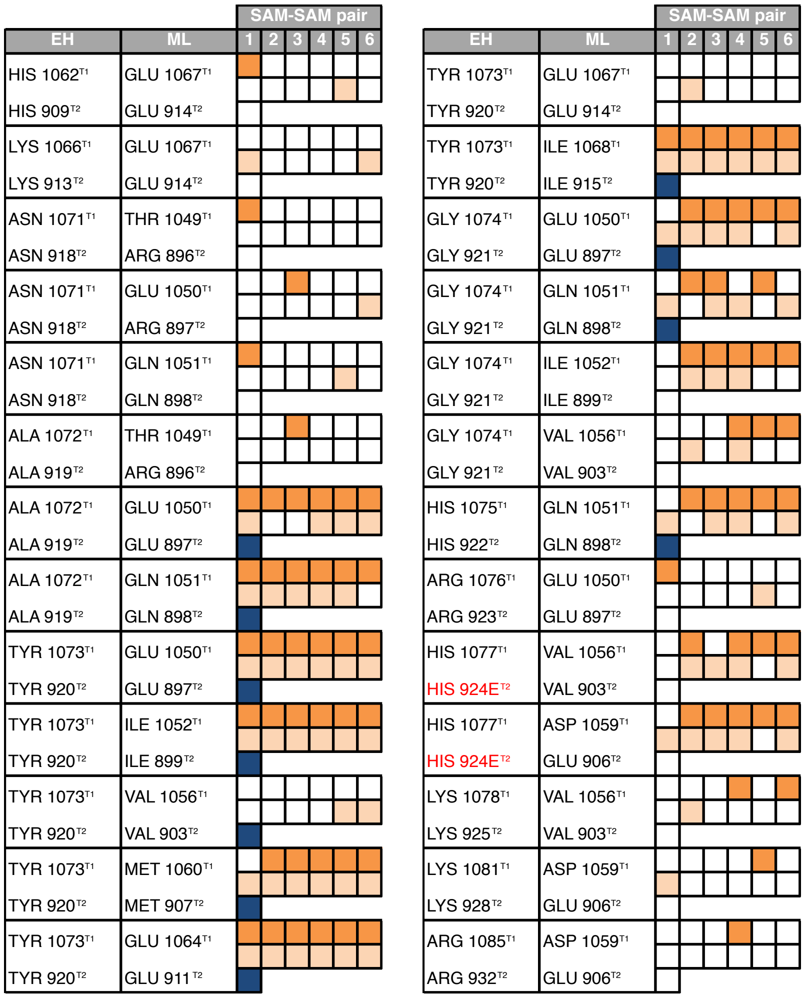

	TNKS SAM		TNKS2 SAM
SAM-SAM pair	crystal form 1	crystal form 2	
1	F_1-D_1	D_1-B_1	A-B
2	D_1-E_1	B_1-C_1	
3	E_1-A_2	C_1-E_2	
4	A_2-C_3	E_2-F_3	
5	C_3-B_3	F_3-A_3	
6	B_3-F_4	A_3-D_4	

Figure S4

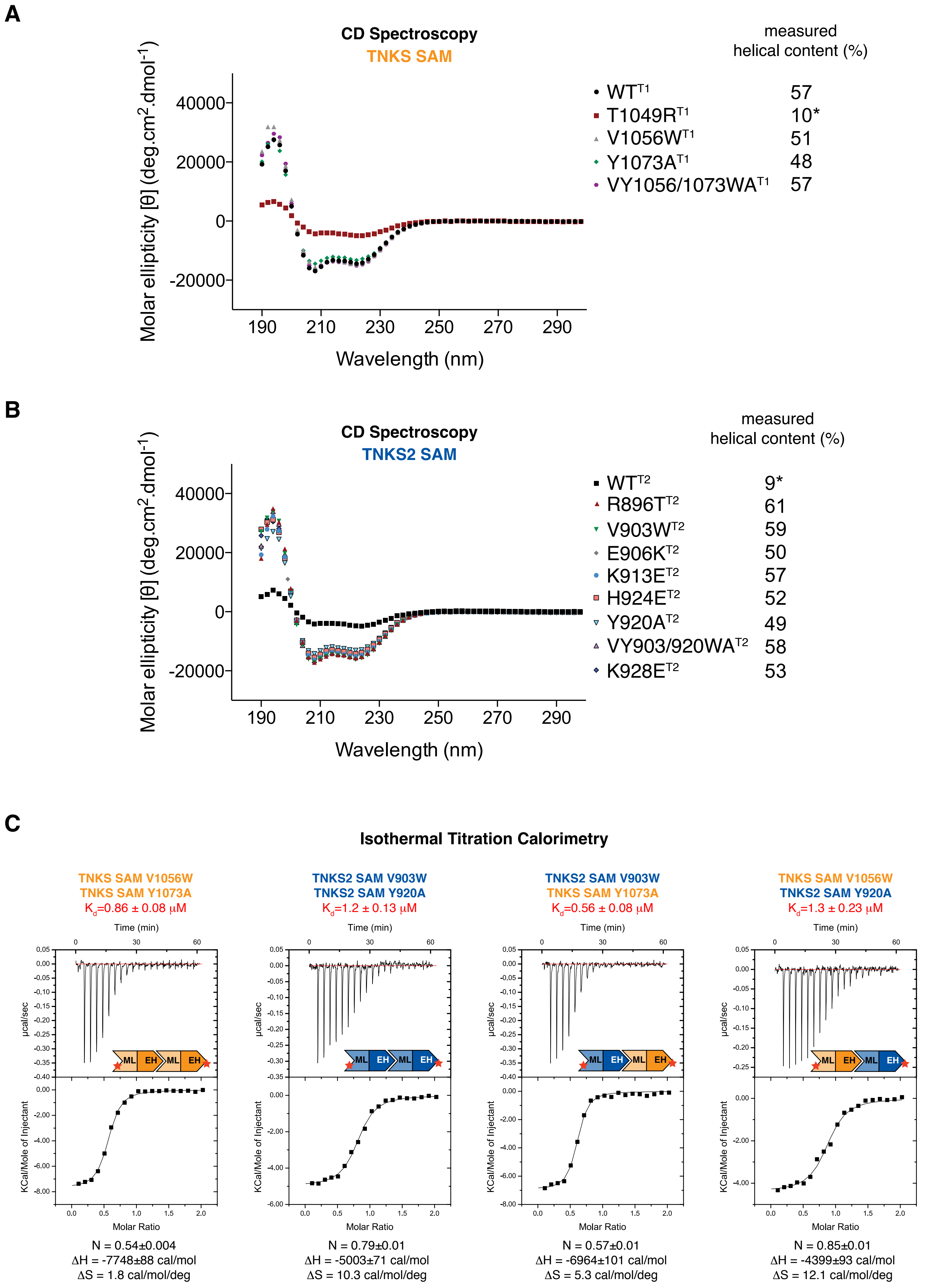
A
CD Spectroscopy
TNKS SAM
measured helical content (%)
WT^T1 57
T1049R^T1 10*
V1056W^T1 51
Y1073A^T1 48
VY1056/1073WA^T1 57
Molar ellipticity [θ] (deg.cm².dmol⁻¹)
Wavelength (nm)
B
CD Spectroscopy
TNKS2 SAM
measured helical content (%)
WT^T2 9*
R896T^T2 61
V903W^T2 59
E906K^T2 50
K913E^T2 57
H924E^T2 52
Y920A^T2 49
VY903/920WA^T2 58
K928E^T2 53
Molar ellipticity [θ] (deg.cm².dmol⁻¹)
Wavelength (nm)
C
Isothermal Titration Calorimetry
TNKS SAM V1056W
TNKS SAM Y1073A
Kd=0.86 ± 0.08 µM
N = 0.54±0.004
ΔH = -7748±88 cal/mol
ΔS = 1.8 cal/mol/deg
TNKS2 SAM V903W
TNKS2 SAM Y920A
Kd=1.2 ± 0.13 µM
N = 0.79±0.01
ΔH = -5003±71 cal/mol
ΔS = 10.3 cal/mol/deg
TNKS2 SAM V903W
TNKS SAM Y1073A
Kd=0.56 ± 0.08 µM
N = 0.57±0.01
ΔH = -6964±101 cal/mol
ΔS = 5.3 cal/mol/deg
TNKS SAM V1056W
TNKS2 SAM Y920A
Kd=1.3 ± 0.23 µM
N = 0.85±0.01
ΔH = -4399±93 cal/mol
ΔS = 12.1 cal/mol/deg
Time (min)
µcal/sec
KCal/Mole of Injectant
Molar Ratio
ML
EH

Figure S5

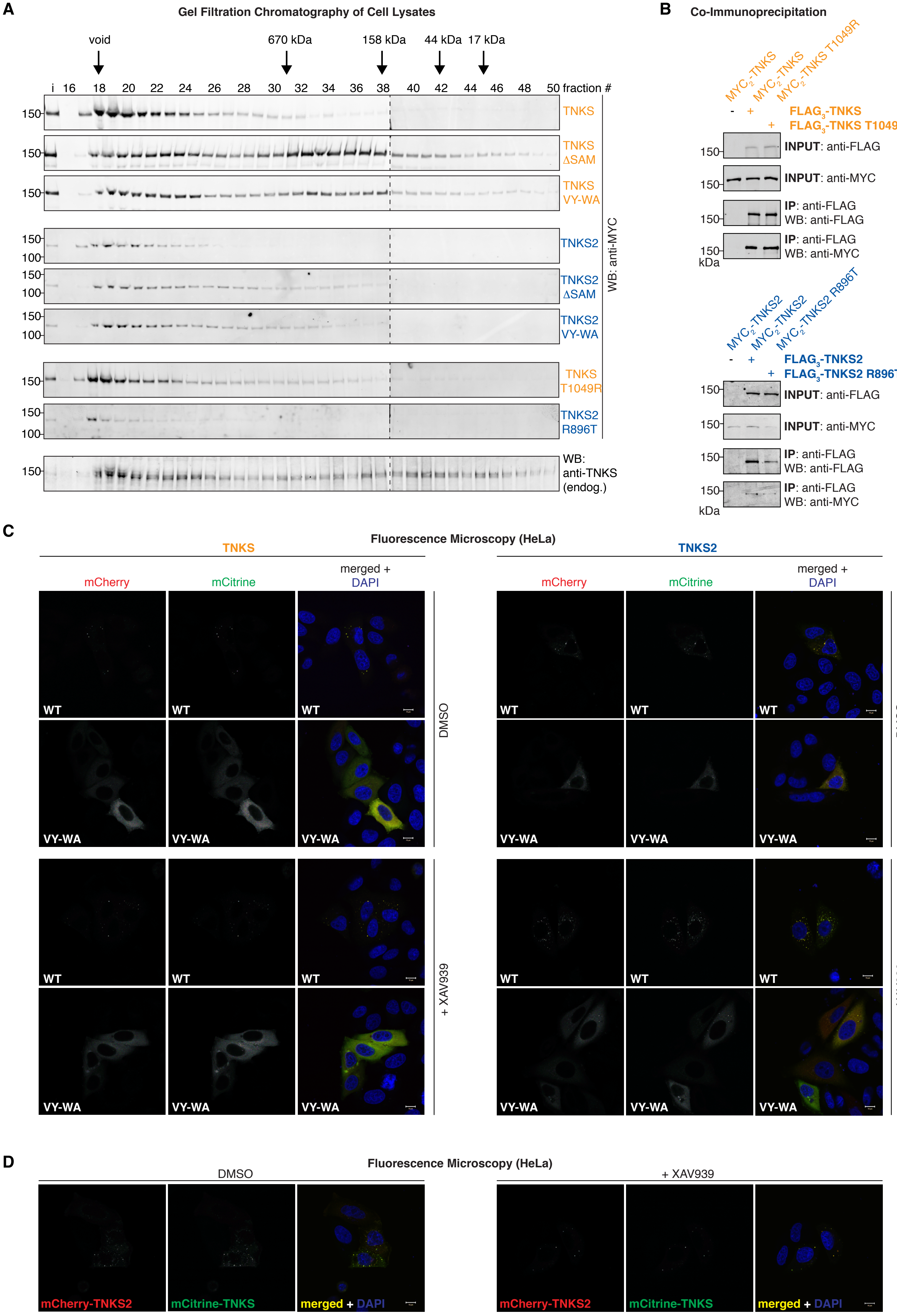
A
Gel Filtration Chromatography of Cell Lysates
void
670 kDa
158 kDa
44 kDa
17 kDa
i 16 18 20 22 24 26 28 30 32 34 36 38 40 42 44 46 48 50 fraction #
150
100
TNKS
TNKS ΔSAM
TNKS VY-WA
TNKS2
TNKS2 ΔSAM
TNKS2 VY-WA
TNKS T1049R
TNKS2 R896T
WB: anti-MYC
WB: anti-TNKS (endog.)
B
Co-Immunoprecipitation
MYC2-TNKS
FLAG3-TNKS
FLAG3-TNKS T1049R
INPUT: anti-FLAG
INPUT: anti-MYC
IP: anti-FLAG WB: anti-FLAG
IP: anti-FLAG WB: anti-MYC
kDa
MYC2-TNKS2
MYC2-TNKS2 R896T
FLAG3-TNKS2
FLAG3-TNKS2 R896T
C
Fluorescence Microscopy (HeLa)
TNKS
TNKS2
mCherry
mCitrine
merged + DAPI
WT
VY-WA
DMSO
+ XAV939
D
Fluorescence Microscopy (HeLa)
DMSO
+ XAV939
mCherry-TNKS2
mCitrine-TNKS
merged + DAPI

Figure S6

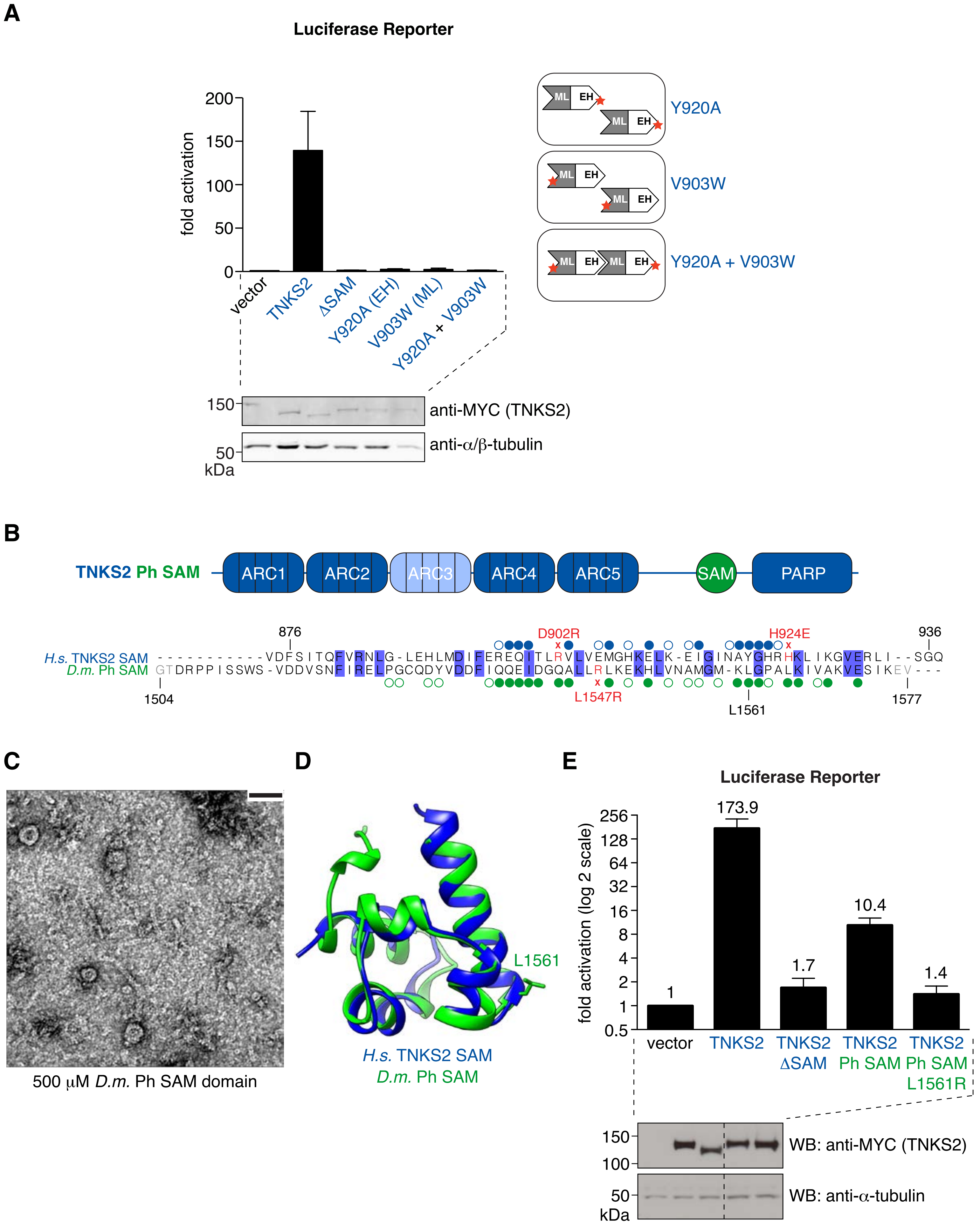
A
Luciferase Reporter
fold activation
200
150
100
50
0
vector
TNKS2
ΔSAM
Y920A (EH)
V903W (ML)
Y920A + V903W
Y920A
V903W
Y920A + V903W
ML
EH
150
50
kDa
anti-MYC (TNKS2)
anti-α/β-tubulin
B
TNKS2 Ph SAM
ARC1
ARC2
ARC3
ARC4
ARC5
SAM
PARP
876
936
1504
1577
D902R
H924E
L1547R
L1561
H.s. TNKS2 SAM
D.m. Ph SAM
C
500 μM D.m. Ph SAM domain
D
L1561
H.s. TNKS2 SAM
D.m. Ph SAM
E
Luciferase Reporter
fold activation (log 2 scale)
256
128
64
32
16
8
4
2
1
0.5
173.9
10.4
1.7
1.4
1
vector
TNKS2
TNKS2 ΔSAM
TNKS2 Ph SAM
TNKS2 Ph SAM L1561R
150
100
50
kDa
WB: anti-MYC (TNKS2)
WB: anti-α-tubulin

Figure S7

Supplemental Figure Legends

Figure S1: Protein expression levels in luciferase reporter assays and immunoprecipitation, related to Figures 1 and 6

(A) to **(D)**, **(F)**, **(G)** HEK293T cells were transiently transfected in technical triplicate for luciferase reporter assays. Two replicates were assessed for luciferase activity (see main figures). A third replicate was analysed by SDS-PAGE and Western blotting as indicated to assess protein expression levels. The dashed line in (F) separates lanes from two different gels/membranes. **(E)** The same INPUT samples shown in the first two lanes of each of the three panels of Figure 6A were analysed by SDS-PAGE and Western blotting on the same gel and membrane for direct comparison, as indicated. MYC_2-TNKS is consistently more highly expressed than MYC_2-TNKS2.

Figure S2: Tankyrase modulation by enzymatic inhibition, AXIN1 and the SAM domain, related to Figures 1 and 2

(A) HEK293T cells were transfected with MYC_2-TNKS2 and treated with the same concentrations of XAV939 as for Figure 1D for 20 h. MYC_2-TNKS2 was immunoprecipitated, and immunoprecipitates were analysed for PAR by Western blotting as indicated. **(B)** TOPFlash transcription reporter assay. MYC_2-TNKS2, TNKS2 G1032W^{T2} or empty vector (16 ng) were co-expressed with increasing amounts of the indicated $FLAG_3$-AXIN1 constructs (0.6 to 20 ng in a two-fold dilution series), either in wild-type form or as Tankyrase-binding deficient mutant (GG27/74RR, GG-RR). Data are expressed relative to the mean reporter activity in the “vector” control without AXIN1. n=3 independent experiments done in technical duplicate; error bars, SEM. Western blots to assess protein expression levels in the assay are shown below. Note that at the highest dose of AXIN1 construct, TNKS2 expression is reduced, accounting for the drop in reporter activation at this dose. **(C)** Ultracentrifugation sedimentation assay as in Figure 2A. Data are from the same experiment as those shown in Figure 2A. **(D)** Electron microscopy analysis of negatively

stained Tankyrase SAM domains at 0.025 mM. See Figure S3A for SEC-MALS analysis. **(E)** TOPFlash transcription reporter assay as in Figure 1C, using 16 ng of MYC_2-TNKS2 constructs. n=3 to 6 independent experiments done in technical duplicate; error bars, SEM. Tankyrase expression levels in the assay are shown below.

Figure S3: SEC-MALS and EM of Tankyrase SAM domains, related to Figures 2 and 5

(A) and **(D)** Molecular weight scatter plots from two separate SEC-MALS analyses for the indicated SAM domains at 0.5 mM (A) and 1 mM in total (0.5 + 0.5 mM for paired mutant domains) (D). Weight-average molecular weights (M_w) and dispersity (Đ) ±SD are indicated. Plotted data points with mean and SD refer to single molecular weight data points (M_i) corresponding to measurement slices. SEC-MALS for V903W^{T2} + Y920A^{T2} and V1056W^{T1} + Y920A^{T2} TNKS2, shown in (D), also revealed monomeric sub-populations (grey data points). M_w and Đ shown for these samples refer to the dimeric sub-population only. TNKS2 WT reference data are the same as shown in Figures 2C and 5B (acquired as part of the same sample set). Likewise, TNKS WT reference data are the same as shown in Figure 5B; TNKS SAM T1049R^{T1} data were acquired as part of the same sample set. See below ((B) and (E)) for an analysis of the corresponding elution fractions. See Figures S5A and B for quality control of purified mutant proteins by CD spectroscopy. **(B)**, **(C)**, **(E)** Samples from SEC-MALS elution fractions (10 μl) were analysed by SDS-PAGE and Coomassie Brilliant Blue staining. Void volume (V_0) and salt peak volume are indicated where corresponding fractions were analysed. i, input (1 μl). Input protein concentration and column are indicated. The dashed line in (C) separates lanes from two different gels. **(F)** Electron microscopy analysis of the indicated TNKS2 SAM domains at 25 μM and 100 μM. Scale bars, 50 nm. Colour coding indicates the degree of abrogated sedimentation, as in Figure 5.

Figure S4: TNKS SAM domain packing and comparative analysis of SAM-SAM contacts in the TNKS and TNKS2 SAM domain crystal structures, related to Figure 4

Non-crystallographic and crystallographic symmetry in two TNKS D1055R^{T1} SAM domain crystal forms gives rise to filaments in the crystals. **(A)** Crystal form 1. **(B)** Crystal form 2. In both cases, the asymmetric unit consists of six SAM domains, but the two asymmetric units are distinct from each other. Left, four adjacent asymmetric units are shown in ribbon representation with one filament in side view highlighted by higher opacity. The six chains within the left-most asymmetric unit are named A_1 to F_1. Subscript numbers of chain identifiers denote the corresponding asymmetric units (1-4). A repeating unit in the filament consists of six protomers. Right, the same assembly rotated by 90° along the y axis reveals the approximate six-fold symmetry. Centroids for each SAM domain in the repeating unit, depicted as spheres, were calculated for TNKS SAM residues 1030-1087. Approximate angles between the centroids of each SAM domain in the axial projection were measured, using the overall centroid of all six centroids as vertex. **(C)** Contact analysis for all SAM-SAM domain pairs observed in all three crystal structures, analysed in UCSF Chimera. SAM-SAM domain pairs are numbered as indicated in the table on the right. A coloured field (TNKS crystal form 1, orange; TNKS crystal form 2, pale orange; TNKS2, blue) represents the occurrence of the respective contact.

Figure S5: CD spectroscopy analysis of purified TNKS and TNKS2 SAM domains and a second ITC experiment, related to Figure 5

(A) and **(B)** Purified proteins at 0.2 mg/ml were analysed by CD spectroscopy. The calculated helical contents of the TNKS and TNKS2 SAM domains from the crystal structures is 61%. Measured helical contents are comparable, with the exception of strongly polymerising SAM domains (TNKS2 SAM and TNKS SAM T1049R^{T1}, denoted by asterisks), where helical contents appear underestimated. Reduced molar ellipticities were previously observed for a polymerising C-terminal truncation of serum amyloid A (SSA), and absorption flattening due to a changed protein

environment in the polymer was proposed to account for this effect (Patke et al., 2012). The observation that SAM domains with mutations in opposite interfaces are still able to form dimers (see Figures S3D and E) provides additional documentation for their correct folding. **(C)** Replicate isothermal titration calorimetry (ITC) experiment as in Figure 5D.

Figure S6: Assessment of TNKS and TNKS2 polymerisation by biochemical assays and microscopy, related to Figure 6

(A) Full-length TNKS and TNKS2 polymerise in a SAM-domain dependent manner. Lysates from cells expressing the indicated MYC_2-Tankyrase constructs were fractionated by size exclusion chromatography. Fractions were analysed by Western blotting as indicated. Dashed lines separate lanes from two different gels/membranes. **(B)** The T1049R^{T1} and R896^{T2} mutations do not appear to affect Tankyrase self-interaction in the context of the full-length protein, at least under the experimental conditions. The indicated $FLAG_3$- and MYC_2-tagged TNKS and TNKS2 constructs were co-expressed in HEK293T cells and $FLAG_3$-Tankyrases immunoprecipitated, as for Figure 6A. Lysates and immunoprecipitates were analysed by SDS-PAGE and Western blotting as indicated. The observations are consistent with the size exclusion experiment shown in (A). **(C)** Tankyrase polymerisation controls localisation. Serum-starved HeLa cells expressing the indicated mCherry- and mCitrine-tagged Tankyrase constructs were either vehicle- or XAV939-treated as indicated, fixed and imaged by fluorescence microscopy. Microscopy data were obtained together with those shown in Figure 6B. Scale bar, 10 μm. **(D)** TNKS and TNKS2 colocalise. Microscopy was performed as for (C). Scale bar, 10 μm.

Figure S7: Polymerisation requirement of TNKS2 to drive Wnt-β-catenin signalling, related to Figure 6

(A) Co-expression of TNKS2 V903W^{T2} and Y920A^{T2}, which would be able to form dimers (see Figures S3D and E), does not rescue the lost ability of the individual mutants to induce Wnt

signalling. TOPFlash transcription reporter assays for the indicated pairs of TNKS2 SAM domain mutant derivatives, performed as for Figure 1C. The total amount of MYC_2-TNKS2 construct per transfection was 16 ng (8 ng + 8 ng for paired TNKS2 mutants). n=3 independent experiments done in technical duplicate; error bars, SEM. Western blots to assess TNKS2 expression levels in the assay are shown below. The schematic on the right illustrates the formation of heterodimers between ML and EH mutant derivatives, which are monomeric on their own, as shown by SEC-MALS (see Figure S3D and E). Although unlikely, we cannot rule out that SAM-SAM interactions within the dimer are weakened by the distal mutations. **(B)-(E)** Polymerisation of an orthologous SAM domain partially compensates for loss of the SAM domain in TNKS2. (**B)** Top, schematic representation of a chimeric TNKS2 construct with the SAM domain of *D. melanogaster* Polyhomeotic (Ph). Bottom, structure-based alignment of the SAM domains of *H. sapiens* TNKS2 (DH902/924RE^{T2}) and *D. melanogaster* Ph (L1547R). Amino acids shown in grey are not resolved in the crystal structures. Mutations introduced for crystallization are indicated in red. Interface residues, as identified by PISA and defined through limited solvent accessibility, and explicit contact residues, as analysed in UCSF Chimera, are indicated by open and closed circles, respectively, as in Figure 4D. For the TNKS2 Ph SAM chimera, TNKS2 residues 876-936 were replaced by Ph residues 1504-1577, as indicated. **(C)** Electron micrograph of negatively stained *D. melanogaster* Ph SAM domain polymers. Scale bar, 50 nm. **(D)** Structural representations of the superimposed TNKS2 and *D. melanogaster* Ph SAM domains (PDB accession code: 1KW4). A L1561R mutation results in loss of Ph SAM domain polymerisation (Kim et al., 2002). **(E)** TOPFlash transcription reporter assay as in Figure 1C. n=6 independent experiments done in technical duplicate; error bars, SEM. Western blots to assess Tankyrase expression levels in the assay are shown below. The dashed line in the Western blot indicates the position where an irrelevant lane has been spliced out. The Ph SAM domain conferred a weak activation of the transcription reporter by TNKS2, illustrating incomplete rescue, but this was abolished by a validated, structure-based polymer-breaking mutation in the heterologous domain (Kim et al.,

2002). Note the log 2 scale.

Supplemental Tables

Table S1. Plasmids used in this study, related to Experimental Procedures
Point mutations and deletions were generated from these plasmids by site-directed mutagenesis (see section "Plasmids" in Extended Experimental Procedures).

plasmid name	species	accession no.	sites	references/information
**bacterial expression constructs**				
pET-His$_6$-MBP-Asn$_{10}$-TEV (1C)-TNKS(1018-1093)	*H. sapiens*	NM_003747.2	LIC v1	1
pET-His$_6$-MBP-Asn$_{10}$-TEV (1C)-TNKS2(867-940)	*H. sapiens*	NM_025235.3	LIC v1	1
pET-His$_6$-MBP-Asn$_{10}$-TEV (1C)-Ph(1502-1587)	*D. melanogaster*	NM_057523.5	LIC v1	1, 2
**mammalian expression constructs / reporter plasmids / vectors**				
pLP-dMYC SD-TNKS	*H. sapiens*	NM_003747.2	*AscI-PacI & loxP*	3, 4
pLP-dMYC SD-TNKS2	*H. sapiens*	NM_025235.3	*AscI-PacI & loxP*	4, 5
pLP-tripleFLAG SD-TNKS	*H. sapiens*	NM_003747.2	*AscI-PacI & loxP*	4
pLP-tripleFLAG SD-TNKS2	*H. sapiens*	NM_025235.3	*AscI-PacI & loxP*	4
pLP-mCitrine C1 SD-TNKS	*H. sapiens*	NM_003747.2	*AscI-PacI & loxP*	4, 6
pLP-mCitrine C1 SD-TNKS2	*H. sapiens*	NM_025235.3	*AscI-PacI & loxP*	4, 6
pLP-mCherry C1 SD-TNKS	*H. sapiens*	NM_003747.2	*AscI-PacI & loxP*	4, 6
pLP-mCherry C1 SD-TNKS2	*H. sapiens*	NM_025235.3	*AscI-PacI & loxP*	4, 6
pLP-tripleFLAG SD-AXIN1	*H. sapiens*	NM_003502.3	*AscI-PacI & loxP*	4, 7
M50 Super 8x TOPFlash	-	-	-	8
M51 Super 8x FOPFlash (TOPFlash mutant)	-	-	-	8
ptkRL	-	-	-	9
pDNR-MCS SA	-	-	-	4

[1] The empty vector was a gift from Dr. Scott Gradia (UC Berkeley) via Addgene (Addgene plasmid # 29654)
[2] The Ph cDNA was a gift from Dr. Robert Kingston (Harvard Medical School) via Addgene (Addgene plasmid # 1925) (Francis et al., 2001)
[3] The pLP-dMyc SD-TNKS plasmid was a kind gift from Dr. Robert Rottapel (OCI, Toronto).
[4] (Colwill et al., 2006)
[5] (Guettler et al., 2011)
[6] The pLP-Citrine C1 SD and pLP-mCherry C1 SD plasmids were a kind gift from Dr. Oliver Rocks (MDC, Berlin).
[7] The AXIN1 cDNA (OriGene) was a kind gift from Dr. Alan Ashworth and Dr. Chris Lord (ICR, London).
[8] M50 Super 8x TOPFlash and M51 Super 8x FOPFlash (TOPFlash mutant) were a gift from Randall Moon (Addgene plasmid # 12456) (Veeman et al., 2003)
[9] ptkRL, originally from Promega, was a kind gift from Dr. Richard Treisman (Francis Crick Institute, London).

Extended Experimental Procedures

Plasmids

Plasmids (see Table S1) were generated from human Tankyrase (TNKS, NM_003747.2), Tankyrase 2 (TNKS2, NM_025235.3), AXIN1 (NM_003502.3) and *D. melanogaster* Ph (NM_057523.5) cDNAs by standard recombinant DNA techniques involving PCR, restriction endonucleases and ligation-independent cloning (Li and Elledge, 2007). For full-length mammalian expression constructs, the initiator methionine codon was omitted. PCRs, including those for site-directed mutagenesis, were performed using KAPA HiFi HotStart DNA polymerase (KAPA Biosystems). Site-directed point and deletion mutant derivatives and chimeric constructs were obtained by using either a modified QuikChange protocol (Agilent Technologies), a two-step megaprimer method or overlap extension. SAM domain deletions in TNKS and TNKS2 encompassed the equivalent regions in both proteins: TNKSΔSAM (Δ1026-1091), TNKS2 ΔSAM (Δ873-938). TNKS2 ΔARC1-5 lacks amino acids 23-794. The TNKS2 xx3xx (L92W, L245W, L560W, L713W) construct was reported previously (Guettler et al., 2011). In the TNKS/TNKS2 SAM domain chimeras, residues $1025\text{-}1093^{T1}$ and $873\text{-}940^{T2}$, which span the variable range within the SAM domains of TNKS and TNKS2, were mutually exchanged. The TNKS2 Ph SAM domain chimera was generated by replacing TNKS2 residues 876-936 by Ph residues 1504-1577 (numbering for *D. melanogaster* ph-p, transcript variant A, NM_057523.5). Two Tankyrase-binding motifs were mutated in AXIN1 GG-RR (GG27/74RR). All other mutant derivatives are named by their respective mutations. All constructs were sequence-verified.

Antibodies and compounds

Antibodies were anti-MYC 9E10 (MA1-81358, Thermo Fisher Scientific), anti-FLAG FG4R (MA1-91878, Thermo Fisher Scientific), anti-FLAG M2 (F3165, Sigma), anti-AXIN1 (C76H11, 2087S, Cell Signaling Technology), anti-AXIN2 (76G6, 2151S, Cell Signaling Technology), anti-PAR (rabbit polyclonal, 4336-BPC-100, Trevigen), anti-TNKS1/2 (H-350, sc-

8337, Santa Cruz Biotechnology), anti-α-tubulin (TU-01, MA1-19162, Thermo Fisher Scientific), anti-α/β-tubulin (2148S, Cell Signaling Technology), and control IgG (sc-2027, Santa Cruz Biotechnology). Secondary antibodies for Western blotting with detection using an Odyssey infrared imaging system (LI-COR) were goat-anti-mouse-DyLight680 (35518, Thermo Fisher Scientific), goat-anti-rabbit-DyLight800 (35571, Thermo Fisher Scientific), IRDye 800CW donkey anti-mouse (926-32212, LI-COR) and IRDye 800CW donkey anti-rabbit (926-32213, LI-COR). Secondary antibodies for Western blotting with ECL detection were goat-anti-mouse-HRP (32430, Thermo Fisher Scientific) and goat-anti-rabbit-HRP (32460, Thermo Fisher Scientific). Secondary antibodies for immunofluorescence microscopy were goat-anti-mouse-DyLight488 (35502, Thermo Fisher Scientific) and goat-anti-rabbit-DyLight633 (35562, Thermo Fisher Scientific). XAV939 was obtained from Dr. Chris Lord (ICR, London).

Mammalian cell culture

HEK293T and SW480 cells, obtained from Dr. Chris Lord (ICR, London), and HeLa cells, obtained from Dr. Chris Bakal (ICR, London), were cultured in a humidified incubator at 37 °C with 5% CO_2 in Dulbecco's Modified Eagle's Medium (DMEM) supplemented with antibiotics (streptomycin sulfate, benzylpenicillin) and 10% FBS (F7524, Sigma). Cells were serum-starved (0.3% FBS) where indicated.

Luciferase reporter assays

The TOPFlash reporter construct contains six TCF/LEF transcription factor binding sites and responds to active β-catenin (Veeman et al., 2003). On day one, HEK293T cells were plated on white 96-well plates (30,000 cells/well). On day two, cells were transfected, in technical triplicate, with the indicated vector (pLP-dMYC SD, pLP-tripleFLAG SD), Tankyrase constructs in pLP-dMYC SD (16 ng/well or as indicated) or the specified amounts of AXIN1 constructs in pLP-tripleFLAG SD, 10 ng/well TOPFlash or FOPFlash, and 2 ng/well ptkRL. DNA was filled up to a

total amount of 50 ng/well using pDNR-MCS SA. Cell media were changed for 100 μl Opti-MEM II (Thermo Fisher Scientific / Gibco), and cells were transfected using Lipofectamine 2000 (Thermo Fisher Scientific / Invitrogen) in a DNA:transfectant ratio of 1:3 in Opti-MEM II. Four h after complex addition, media were changed for DMEM with 0.3% FBS. XAV939 was added in a two-fold dilution series from 9.8 nM to 10 μM at the media change step, maintaining a constant DMSO concentration of 0.2%. Twenty h after media change, cells from two technical replicates were lysed using Passive Lysis Buffer (Promega) and processed for luminometry using the Dual-Luciferase Reporter Assay system (Promega). Plates were read using a Perkin Elmer VICTOR X5 plate reader using an integration time of 5 s. Upon background subtraction, ratios of Firefly Luciferase to Renilla Luciferase signals were calculated for each of the two technical replicates. The means of the technical replicates were further analysed as indicated in the figure legends. Data shown are from at least three independent experiments performed in technical duplicate, as detailed in the figure legends. A third technical replicate was processed for analysis by SDS-PAGE and Western blotting to assess protein levels (see Figure S1). Immunodetection in Western blots was done using an Odyssey infrared imaging system (LI-COR) or ECL.

Expression and purification of TNKS and TNKS2 SAM domains for crystallisation, electron microscopy, multi-angle light scattering, circular dichroism spectroscopy and isothermal titration calorimetry

Human TNKS SAM (1018-1093) and TNKS2 SAM (867-940) domain constructs were expressed as His_6-MBP-Asn_{10} fusion proteins in *E. coli* BL21-CodonPlus(DE3)-RIL (Stratagene) grown in TB media. Expression was induced at an OD_{600} of 2.0 with 0.5 mM IPTG overnight at 18 °C. Cells were collected by centrifugation, resuspended in a buffer containing 50 mM Tris-HCl (pH 7.5), 1.5 M NaCl (high to limit SAM domain polymerisation), 5 mM imidazole (pH 7.5), 10 mM β-mercaptoethanol and protease inhibitors (1 mM PMSF, 1 μg/ml leupeptin, 1 μg/ml aprotinin, 1 μg/ml pepstatin A) (pellet from 1 l of culture resuspended in 50 ml of buffer), lysed by

homogenisation using an EmulsiFlex-C5 homogenizer (Avestin) or by sonication using a Vibra-Cell sonicator (Sonics & Materials), and centrifuged to remove insoluble material. Lysates were briefly sonicated to shear *E. coli* genomic DNA and filtered through a 0.45 μm filter. Filtered lysates were loaded onto 5 ml Ni HisTrap HP affinity columns (GE Healthcare). Columns were washed with at least 5 column volumes (CV) of wash buffer (identical to lysis buffer but lacking protease inhibitors). His_6-MBP fusion proteins were eluted with a linear imidazole gradient (5 to 250 mM imidazole, pH 7.5) in a buffer also containing 50 mM Tris-HCl (pH 7.5), 1.5 M NaCl and reducing agent. To remove the His_6-MBP tag, the fusion proteins were incubated with recombinant TEV protease overnight while dialysing against 50 mM Tris-HCl (pH 7.5), 200 mM NaCl (1.5 M NaCl for TNKS SAM T1049R and TNKS2 SAM), 10 mM β-mercaptoethanol. The His_6-MBP-Asn_{10} tag was removed by another Ni affinity chromatography step using a 5 ml Ni HisTrap HP affinity column (GE Healthcare), with the exception of TNKS SAM T1049R and TNKS2 SAM. The latter two were diluted 10-fold and incubated for ≈48 h with 25 ml HisPur Ni-NTA Superflow Agarose (Thermo Fisher Scientific) to efficiently remove the His_6-MBP tag entrapped by the polymerising proteins and further dialysed against 50 mM Tris-HCl (pH 7.5), 200 mM NaCl, 10 mM β-mercaptoethanol. All proteins were applied onto 5-ml HisTrap Q HP columns (GE Healthcare) for ion-exchange chromatography. The proteins were eluted in a linear NaCl gradient (0.2 to 1 M NaCl) in a buffer also containing 50 mM Tris-HCl (pH 7.5) and 10 mM β-mercaptoethanol. The resulting protein was dialysed against 25 mM HEPES-NaOH (pH 7.5), 1.5 M NaCl, 10 mM β-mercaptoethanol, concentrated and subjected to size-exclusion chromatography on a 120-ml HiLoad 16/60 Superdex 75 (prep grade, GE Healthcare) equilibrated in 25 mM HEPES-NaOH (pH 7.5), 1.5 M NaCl, 2 mM TCEP. Pure fractions were pooled, concentrated and flash-frozen in liquid nitrogen. *D. melanogaster* Ph SAM domain (1502-1587) was purified as TNKS/TNKS2 SAM, but instead of 1.5 M NaCl, only 500 mM NaCl were used. Before experiments, proteins were dialysed against 25 mM HEPES-NaOH (pH 7.5), 200 mM NaCl, 2 mM TCEP overnight to lower the NaCl concentration kept high to limit polymer formation during

purification. Proteins were quantified spectrophotometrically, using extinction coefficients calculated by ExPASy ProtParam (Gasteiger et al., 2005), and by amino acid analysis (Protein & Nucleic Acid Chemistry Facility, Department of Biochemistry, University of Cambridge, UK). Given that the Tankyrase SAM domains only contain a single aromatic residue enabling A_{280} measurements, spectrophotometrically measured SAM domain concentrations were corrected by a calibration factor based on amino acid analysis performed earlier.

Protein crystallisation

Initial crystal hits of TNKS2(867-940) DH902/924RE^{T2} obtained from the Index HT sparse-matrix screen (Hampton Research) were optimised by mixing 1 μl of a 13.5 mg/ml protein solution (in 25 mM HEPES-NaOH (pH 7.5), 100 or 200 mM NaCl, 2 mM TCEP) with 1 μl of a precipitant solution containing 0.1 M Tris-HCl (pH 8.5), 0.2 M ammonium acetate, 20% PEG 3350 in a hanging-drop vapour-diffusion setup at 12 °C, using a 24-well setup and 1 ml of precipitant solution in the wells. Streak seeding with horse tail hair (Nenê) was performed one day after setting up crystallisation trays with seeds from crystals obtained earlier. Crystals grew within 2 days upon seeding. Before flash-freezing in liquid nitrogen, crystals were cryo-protected in a stabilisation solution identical to the precipitant solution but also containing 30% PEG 400.

Crystals for TNKS SAM (1018-1093) D1055R^{T1} were grown by sitting-drop vapour diffusion at 12 °C using the Index HT (Hampton Research) sparse matrix screen by mixing 150 nl of protein solution at 10 mg/ml (in 25 mM HEPES-NaOH (pH 7.5), 200 mM NaCl, 2 mM TCEP) with 150 nl of a precipitant solution containing 0.2 M $MgCl_2$, 0.1 M Bis-Tris (pH 5.5), 25% PEG 3350. Crystals grew within 3 weeks. Before flash-freezing in liquid nitrogen, crystals were cryo-protected in a stabilisation solution identical to the precipitant solution but also containing 25% ethylene glycol.

Data collection, structure determination and structure analyses

Diffraction data for TNKS2 SAM DH902/924RE^{T2} were collected at the Diamond Light Source on beamline IO3. Data were processed and scaled using XDS (Kabsch, 2010) and merged using AIMLESS (Winn et al., 2011). The structure was solved by molecular replacement using Phaser (Adams et al., 2010; McCoy et al., 2007) with a homology model generated by Modeller/HHPred (Šali et al., 1995) based on 5 templates (PDB codes: 1v85_A, 3bs5_B, 3bq7_A, 2e8o_A, 2gle_A) as a search model. The structure model was generated iteratively by manual model building in Coot (Emsley et al., 2010) and refinement using Phenix Refine (Adams et al., 2010) and BUSTER (Bricogne et al., 2016).

Diffraction data for two crystal forms obtained for TNKS SAM D1055R^{T1} were collected at the Diamond light source on beamline IO3, processed using XDS (Kabsch, 2010) and scaled and merged using AIMLESS (Collaborative Computational Project, 1994). For TNKS SAM crystal form 1, five datasets from a total of three crystals were analysed using BLEND (Foadi et al., 2013) giving a Linear Cell Variation of 0.7 Å. BLEND was subsequently run in synthesis mode on all datasets and merging statistics indicated a high data resolution cutoff of 2.5 Å in order to achieve a half-dataset correlation coefficient CC(1/2) of 0.3 (Karplus and Diederichs, 2012). A second TNKS SAM crystal form was processed using merged data from two crystals as above. The structure was solved by molecular replacement in Phaser (Adams et al., 2010; McCoy et al., 2007) using a homology model for TNKS SAM D1055R^{T1}, generated by SWISS-MODEL (Arnold et al., 2006), based on the crystal structure of TNKS2 SAM DH902/924RE^{T2}. The structure model was generated iteratively by manual model building in Coot (Emsley et al., 2010) and refinement using Phenix Refine (Adams et al., 2010) and BUSTER (Bricogne et al., 2016). High-resolution cut-offs were defined as described by Karplus and Diederichs (Karplus and Diederichs, 2012). During refinement, side chain atoms not accounted for by density due to residue mobility were removed. Structure coordinates and experimental structure factors have been deposited in the Protein Data Bank (PDB IDs 5JRT, 5JU5 and 5JTI).

The structural representations were generated and structural analyses performed using UCSF Chimera (a product of the Resource for Biocomputing, Visualization, and Informatics at the University of California, San Francisco, supported by NIGMS P41-GM103311) (Pettersen et al., 2004). For the electrostatics analysis shown in Figure 3B, the $D902R^{T2}$ and $H924E^{T2}$ mutations were reverted to wild-type; incompletely resolved side-chains were added in full, and the structure was energy-minimised using UCSF Chimera. Interface residues in SAM-SAM domain pairs for Figures 4D, 4E and S7B were identified using the PISA web server (Krissinel and Henrick, 2007). Head-to-tail SAM-SAM domain contacts were analysed using the 'Find Clashes/Contacts' function in UCSF Chimera (allowed overlap: -0.4 Å; H-bond overlap reduction: 0) (Pettersen et al., 2004). Note that PISA defines interface residues on the basis of (at least partial) solvent inaccessibility. Thus, not every interface residue will be involved in an explicit contact.

Bioinformatics analyses

Sequences of Tankyrase orthologues used for the multiple sequence alignment in Figure 4D have the following NCBI accession numbers (in the same order as within the figure): NP_003738.2 (Homo sapiens), NP_780300.2 (*Mus musculus*), NP_989671.1 (*Gallus gallus*), XP_012428885.1 (*Taeniopygia guttata*), XP_004911090.1 (*Xenopus tropicalis*), XP_005451454.1 (*Oreochromis niloticus*), XP_003445711.1 (*Oreochromis niloticus*), NP_079511.1 (*Homo sapiens*), NP_001157107.1 (*Mus musculus*), NP_989672.1 (*Gallus gallus*), XP_012429997.1 (*Taeniopygia guttata*), NP_001017008.2 (*Xenopus tropicalis*), XP_005471626.1 (*Oreochromis niloticus*), XP_687410.4 (*Danio rerio*), NP_001082884.1 (*Danio rerio*), NP_651410.1 (*Drosophila melanogaster*), XP_002121662.3 (*Ciona intestinalis*), XP_001897965.1 (*Brugia malayi*), XP_789260.3 (*Strongylocentrotus purpuratus*), XP_005099438.1 (*Aplysia californica*), CDS23197.1 (*Echinococcus granulosus*), XP_006825651.1 (*Saccoglossus kowalevskii*), XP_012563232.1 (*Hydra vulgaris*), XP_011410275.1 (*Amphimedon queenslandica*). Clear TNKS and TNKS2 orthologues seem to first appear in the fish *Oreochromis niloticus*, which has two

TNKS-like Tankyrases and one TNKS2-like Tankyrase. Another fish species, *Danio rerio*, only appears to have two TNKS-like Tankyrases. Conservation-based and structure-based sequence alignments were generated using ClustalX (Larkin et al., 2007) and UCSF Chimera (Pettersen et al., 2004), respectively. Alignments, coloured by % identity, were visualised using Jalview (Waterhouse et al., 2009). For Figure S7B, the SAM domain sequence of *Drosophila melanogaster* Polyhomeotic (NM_057523.5) was obtained from the SAM domain crystal structure (PDB accession code 1KW4) (Kim et al., 2002).

Ultracentrifugation sedimentation assays

SAM domains were purified as described above (see "Expression and Purification of TNKS and TNKS2 SAM Domains …", for the experiment shown in Figures 2A and S2C) or affinity-purified on a small scale using amylose resin (NEB) (for experiments shown in Figures 3C and 5A). 50 μl of a 25 μM solution (approximately 25 μM for experiments shown in Figures 3C and 5A) of SAM domains (in 25 mM HEPES-NaOH (pH 7.5), 200 mM NaCl, 2 mM TCEP for Figure 2 and 50 mM Tris-HCl (pH 7.5), 200 mM NaCl, 10 mM β-mercaptoethanol for Figures 3 and 5) were subjected to centrifugation at 200,000 x g (average speed) at 20 °C for 1 h in a TLA100 rotor (Beckman Optima TLX centrifuge). Supernatants were removed and pellets resuspended in 50 μl of SDS sample buffer. Equivalent amounts of total, supernatant and pellet samples were analysed by SDS-PAGE on Tris-Tricine gels and Coomassie Brilliant Blue staining.

Circular dichroism spectroscopy

Proteins at 0.2 mg/ml were dialysed into 10 mM Tris-HCl (pH 7.5), 200 mM NaF. CD spectra were collected on a Jasco J-720 spectrometer using a 0.1 cm pathlength cell at the ISMB Biophysics Centre (London). Spectra were averaged over 5 scans and corrected for buffer baseline using CDtool (Lees et al., 2004). The analysis of the spectra was performed in DICHROWEB using the CDSSTR algorithm with SP175 as the reference set (Whitmore and Wallace, 2004; 2008).

Electron microscopy

3 μl of purified TNKS2 SAM domains at 25 μM or 100 μM and TNKS SAM domains at 25 μM, 0.25 mM, 0.5 mM, 1 mM or 2 mM (in 25 mM HEPES-NaOH (pH 7.5), 200 mM NaCl, 2 mM TCEP) were applied to glow-discharged carbon-coated grids and negatively stained with 2% (w/v) uranyl acetate. Electron micrographs were recorded at magnifications of either 11,000x (Figures 2 and S2) or 42,000x (Figures 5, S3 and S7) on an FEI Tecnai 12 electron microscope operating at an accelerating voltage of 120 kV, equipped with an F114 1k x 1k CCD detector (TVIPS, Germany).

SEC-MALS

20 μl of 0.5 mM, 1 mM or 2 mM protein samples were resolved by size exclusion chromatography on an Agilent Prostar HPLC system with a TSKgel G2000SWxl or G3000SWxl column (Tosoh Biocience LLC). Separation was performed in 25 mM HEPES-NaOH (pH 7.5), 200 mM NaCl, 2 mM TCEP at a flow rate of 1 ml/min. In-line light scattering was measured using DAWN Heleos-II light scattering instrument (Wyatt) and differential refractive index using an Optilab rEX instrument (Wyatt). Data analysis, using the Zimm light scattering model and a dn/dc of 0.185 ml/g, was performed using Wyatt's ASTRA software. Weight-average molecular weights (M_w) and dispersities (Đ) with standard deviations were calculated for the elution peak areas in ASTRA. Two separate experiments were performed, and weighted averages of M_w and Đ and associated SD calculated. Weighting was performed by the number of data slices i. Plotted mean and error bars (SD) in scatter plots refer to molecular weights measured in individual slices (data points) M_i. Note that strongly polymerising SAM domains show a lower dRI signal, which is due to a more spread-out elution behaviour. The average SAM domain concentrations (from dRI measurements) in the selected peak areas for the wild-type SAM domain proteins analysed for Figure 2C were as follows: 0.5 mM TNKS SAM sample: 29 μM; 2 mM TNKS SAM sample: 108 μM; 0.5 mM TNKS2 SAM sample: 10 μM. The surprisingly late elution of TNKS2 SAM and TNKS SAM T1049R[T1] may indicate an interaction or entanglement of the long polymers with the

solid phase of the gel filtration column. An earlier elution peak close to the void volume did not contain any SAM domain (see Figure S3B).

Isothermal titration calorimetry

All proteins were dialysed in parallel into binding buffer containing 25 mM HEPES-NaOH (pH 7.5), 200 mM NaCl, 1 mM TCEP. Titrations were carried out at 25 °C on an ITC200 MicroCalorimeter (Microcal/GE Healthcare). TNKS2 SAM Y920A^{T2} or TNKS SAM Y1073A^{T1} at 500 µM were serially injected in 2-µl increments into TNKS2 SAM V903W^{T2} or TNKS SAM V1056W^{T1} at 50 µM or buffer only. ITC data were processed using the Origin7 software (MicroCal). For all calculations, the signals obtained upon titrating injectant into buffer were subtracted. Integrated data were fitted using a one-site binding model.

TNKS2 auto-PARylation assays and PAR analysis

HEK293T cells were seeded on 15-cm cell culture dishes at 9 x 10^6 cells per dish. On the next day, cells were transfected with 30 µg per dish of pLP-dMYC SD empty vector or the indicated TNKS2 constructs (2 dishes per construct) using calcium phosphate. 24 h post-transfection, cells were scraped in ice-cold PBS and cell pellets lysed in 1 ml high-salt RIPA buffer (50 mM HEPES-NaOH (pH 7.5), 750 mM NaCl, 1% Triton X-100, 0.5% sodium deoxycholate, 0.1% SDS, 1 mM DTT, 2 µM ADP-HPD PARG inhibitor (Merck) and protease inhibitors (Pierce protease inhibitor tablets, EDTA-free, Thermo Fisher Scientific). Cell lysates were briefly sonicated on ice to shear DNA and cleared by centrifugation (20,817 xg, 15 min) at 4 °C. The cleared cell lysates were incubated with 75 µl (packed volume) of pre-equilibrated anti-c-Myc-agarose resin (9E10; Thermo Fisher Scientific or Takara Bio) for 3 h, rotating at 4 °C. Resin samples were washed 9 times with 4 ml lysis buffer and 3 times with 1 ml PARP assay buffer (50 mM HEPES–NaOH (pH 7.5), 150 mM NaCl, 0.01% Triton X-100, 10% glycerol, 1 mM DTT). After the final wash step, 75 µl of PAR assay buffer were added to the resin and 40 µl of the suspension removed

for analysis by SDS-PAGE and Commassie Brilliant Blue staining to estimate protein levels. The remaining samples were centrifuged and the buffer removed. Sample volumes were adjusted to 54 µl by addition of PARP assay buffer, estimating that the resin after buffer removal entraps approximately half its volume of buffer. 6 µl of 10x NAD^+ stock (10 mM NAD^+ and 5 µCi/6 µl ^{32}P-NAD^+ (Perkin Elmer)) were added to each sample and PARP reactions performed for 30 min at 30 °C on a horizontal shaker at 800 rpm. 30 µl of suspension were taken from each sample, boiled with 2x SDS sample buffer and comparable amounts of MYC_2-Tankyrases, based on previous protein level estimates, analysed by SDS-PAGE and Coomassie Brilliant Blue staining. The gel was dried on a gel dryer and exposed to a phosphoimager plate (GE Healthcare) for 24 h. If MYC_2-Tankyrase levels were low, their levels in the PARylation reaction were analysed by Western blotting, using equivalent amounts of immunoprecipitate samples set aside prior to the *in-vitro* PARylation reaction. Endogenous PARylation (prior to the *in-vitro* PARylation reaction) was assessed by Western blotting using an anti-PAR antibody.

PAR chains were analysed essentially as described previously (Alvarez-Gonzalez and Jacobson, 1987; Panzeter and Althaus, 1990), as follows. The PARP assay samples remaining upon SDS-PAGE analysis were precipitated by addition of an equal volume of ice-cold 40% (w/v) trichloroacetic acid (TCA) and incubated on ice for 15 min. The resins were settled by slow centrifugation (1,000 xg, 10 s) and the supernatants recovered. Precipitates were collected by centrifugation (16,300 xg, 10 min) and the pellets washed 3 times with 100 µl 5% TCA followed by 2 wash steps with 100 µl ice-cold diethyl ether (Sigma-Aldrich). The pellets were dried using a Micro-Cenvac NB-503CIR vacuum concentrator at 50 °C for 10 min at 2,500 rpm. The PAR chains were detached from the precipitated material by resuspending the dried pellets in 100 µl of 10 mM Tris (base), 1 mM EDTA, pH 12 and incubated for 3 h at 60 °C. PAR was extracted with 100 µl of phenol/chloroform/isoamyl alcohol (25:24:1) and the aqueous phase recovered followed by two rounds of phenol back-extraction with 100 µl chloroform/isoamyl alcohol (24:1). After the final phenol back-extraction, the samples were dried in the vacuum concentrator at 50 °C for 60 min at

2,500 rpm. PAR was dissolved in 10 μl of PAR sample buffer (50% urea, 25 mM NaCl, 4 mM EDTA (pH 8.0), 0.02% xylene cyanol, 0.02% bromophenol blue). The amounts of ^{32}P-labelled PAR chains were quantified by Cerenkov counting and equal counts per minute (cpm) of samples were loaded onto a 40% acrylamide/bisacrylamide (19:1) sequencing gel (in a kuroGEL Verti 1824 apparatus), except for the negative control samples (vector only and TNKS2 G1032W^{T2}), for which the whole sample was loaded. The sequencing gel electrophoresis was performed in 1x TBE running buffer using a constant current of 25 mA. The electrophoresis was stopped when the bromophenol blue band had migrated 11 cm from the bottom of the wells. The gel was fixed for 1 h with 40% methanol, 10% acetic acid, 3% glycerol (to protect it from cracking during drying) and dried using a temperature gradient cycle to 60 °C for 2-3 h. The gel was exposed for 24 h to a phosphoimager plate (GE Healthcare). Phosphoimager plates were read using a Typhoon FL9500 biomolecular imager (GE Healthcare) and analysed using ImageQuant TL. Xylene cyanol and bromophenol blue were used to determine the PAR chain length (Alvarez-Gonzalez and Jacobson, 2011).

Tankyrase-Tankyrase co-immunoprecipitation

HEK293T cells were seeded on 10-cm cell culture dishes at 6 x 10^6 cells per dish. On the next day, cells were transfected with expression plasmids for pLP-tripleFLAG SD-TNKS (wild-type or T1049R^{T1}) or pLP-tripleFLAG SD-TNKS2 (wild-type or R896T^{T2}) as bait or empty vector as control (10 μg) and co-transfected with the indicated pLP-dMYC SD-TNKS or pLP-dMYC SD-TNKS2 constructs (10 μg) using calcium phosphate. After 24 h, cells were scraped in ice-cold PBS and collected by centrifugation. Cells were lysed in 900 μl of lysis buffer containing 50 mM HEPES-NaOH (pH 7.5), 200 mM NaCl, 0.2% Triton X-100, 10% glycerol, 5 mM DTT and protease inhibitors (Pierce protease inhibitor tablets, EDTA-free, Thermo Fisher Scientific). Cell lysates were briefly sonicated on ice to shear genomic DNA and clarified by centrifugation at 20,817 xg for 15 min at 4 °C. Immunoprecipitation was performed with 30 μl of packed,

equilibrated anti-FLAG M2 agarose (Sigma), rotating for 3 h at 4 °C. The immunoprecipitates were washed 5 times with 1 ml lysis buffer (without protease inhibitors and with only 1 mM DTT). Immunoprecipitated proteins were recovered by boiling the resin in 50 μl of 2x SDS-PAGE sample buffer. Lysate samples (input) and immunoprecipitates were analysed by SDS-PAGE (10%) and Western blotting. Western blots were imaged using an Odyssey infrared imaging system (LI-COR).

Endogenous AXIN1 immunoprecipitation

HEK293T cells were seeded on 10-cm cell culture dishes at 6 x 10^6 cells per dish. On the next day, the media were replaced with DMEM containing 0.3% FBS. (Serum starvation was performed to match conditions used in the luciferase reporter assays.) Each dish was transfected with 10 μg of pLP-dMYC SD empty vector or the indicated TNKS2 constructs using calcium phosphate. In order to maintain its limiting cellular concentrations, we did not overexpress AXIN bait protein but instead relied on endogenous AXIN1. 24 h post-transfection, cells were scraped in ice-cold PBS and cell pellets lysed in 0.75 ml of the stringent RIPA buffer (50 mM HEPES-NaOH (pH 7.5), 150 mM NaCl, 1% Triton X-100, 0.5% sodium deoxycholate, 0.1% SDS, 1 mM DTT, 2 μM ADP-HPD PARG inhibitor (Merck) and protease inhibitors (Pierce protease inhibitor tablets, EDTA-free, Thermo Fisher Scientific). Cell lysates were briefly sonicated on ice to shear DNA and cleared by centrifugation (20,817 xg, 15 min) at 4 °C. Cleared cell lysates were incubated rotating at 4 °C overnight with rabbit anti-AXIN1 (C76H11 clone, Cell Signaling Technologies) at a 1:50 dilution, as recommended by supplier. Rabbit IgG antibody (sc-2027, Santa Cruz Biotechnology), at comparable concentration, was used as a negative control. 25 μl of pre-equilibrated Pierce Protein A/G magnetic resin (Thermo Fisher Scientific) were incubated with the samples for 2 h rotating at 4 °C. The resin samples were washed extensively: 6 times with 1 ml RIPA buffer (without ADP-HPD and protease inhibitors) each. Immunoprecipitated proteins were recovered by boiling the resin samples in 60 μl of 2x SDS-PAGE sample buffer. Lysate samples (input) and

immunoprecipitates were analysed by SDS-PAGE and Western blotting. Western blots were imaged using an Odyssey infrared imaging system (LI-COR).

Assessment of Tankyrase inhibition by XAV939

HEK293T cells were seeded on six-well cell culture dishes at 3.5 x 10^5 cells per well. On the next day, cells were transfected in Opti-MEM II (Thermo Fisher Scientific / Gibco) with expression plasmids for pLP-dMYC SD-TNKS2 or empty vector as control (1 μg) using Lipofectamine 2000 in a DNA:transfectant ratio of 1:3. Four h after transfection complex addition, media were changed for DMEM with 0.3% FBS, to match conditions of the luciferase reporter assay. XAV939 was included in a two-fold dilution series from 9.8 nM to 10 μM at the media change step, maintaining a constant DMSO concentration of 0.2%. Twenty h after XAV939 treatment, cells were lysed in 50 mM HEPES-NaOH (pH 7.5), 150 mM NaCl, 0.1% SDS, 0.5% sodium deoxycholate, 1% Triton X-100, 5 mM DTT, 2 μM ADP-HPD PARG inhibitor (Merck) and protease inhibitors (Pierce protease inhibitor tablets, EDTA-free, Thermo Fisher Scientific). Cell lysates were cleared by centrifugation (20,817 xg, 15 min) at 4 °C. MYC_2-TNKS2 was immunoprecipitated using 20 μl of packed, equilibrated anti-MYC 9E10 agarose (Takara) per sample on a rotating wheel for 3 h at 4 °C. Immunoprecipitates were washed 3 times with 1 ml wash buffer (as lysis buffer, but without PARG inhibitor, protease inhibitors and containing only 1 mM DTT). Resin samples were taken up in 20 μl 4x SDS-PAGE sample buffer, boiled and processed for SDS-PAGE and Western blotting.

Cell lysate fractionations by gel filtration chromatography

HEK293T cells were seeded on 10-cm cell culture dishes at 6 x 10^6 cells per dish. On the next day, cells were transfected with the indicated pLP-dMYC SD-TNKS or pLP-dMYC SD-TNKS2 constructs or empty vector (20 μg) using calcium phosphate. Cells were maintained in DMEM with 10% FBS. After 24 h, cells were washed in ice-cold PBS, scraped in ice-cold PBS, collected by centrifugation and flash-frozen in liquid nitrogen. Cells were lysed in 600 μl of

lysis/fractionation buffer containing 50 mM HEPES-NaOH (pH 7.5), 200 mM NaCl, 0.2% Triton X-100, 2 mM TCEP and protease inhibitors (Pierce protease inhibitor tablets, EDTA-free, Thermo Fisher Scientific). Lysates were briefly sonicated (3 s at 20% output on a Vibra-Cell sonicator (Sonics & Materials) equipped with a micro-tip). After 10 min extraction time on ice, cell lysates were cleared by centrifugation (20,817 xg, 15 min) at 4 °C. 500 μl of cleared lysates were subjected to size exclusion chromatography on a Superose 6 10/300 GL column (GE Healthcare) equilibrated in lysis/fractionation buffer (without protease inhibitors, which were only included in the sample) with a flow rate of 400 μl/min and collection of 400-μl fractions. 200 μl of each fraction were subjected to acetone precipitation with 800 μl acetone at -20 °C. Precipitates were collected by centrifugation (20,817 xg, 10 min) at 4 °C, air-dried and taken up in 10 μl of 2x SDS-PAGE sample buffer. 5 μl of lysate (input) and fraction samples (total 10 μl corresponding to 200 μl of fractionated sample) were analysed by SDS-PAGE and Western blotting. The column was calibrated using a gel filtration standard protein mix (BIO-RAD, 151-1901) under identical fractionation conditions. Note that apparent molecular weights of eluting proteins are not only sensitive to polymeric status but also to interactions in the cell lysates and protein/protein complex shape.

Fluorescence microscopy

HeLa or SW480 cells were plated in 6-well dishes with glass coverslips (200,000 cells/well). On the following day, the media were changed for Opti-MEM II (Thermo Fisher Scientific / Gibco), and the cells were transiently transfected with 1 μg of the indicated MYC_2-, mCitrine- or mCherry-tagged TNKS2 constructs each, using Lipofectamine 2000 (Thermo Fisher Scientific / Invitrogen) in a DNA:transfectant ratio of 1:3. 4 h after complex addition, transfection mix was replaced by DMEM containing 0.3% FBS supplemented with either DMSO (0.04%) or 2 μM XAV939. 20 h after media change, cells were fixed by addition of 4% formaldehyde (AMRESCO) and incubation at 37 °C for 10 min. For immunofluorescence microscopy, cells were

washed once with PBS, permeabilised with 0.2% Triton X-100 in PBS for 10 min. Permeabilisation buffer was replaced by PBS. Non-specific epitopes were blocked with blocking solution (PBS, 5% (w/v) dry milk powder, 10% FBS, 0.05% Tween-20) for 1 h. Cells were immunostained with primary (anti-MYC 9E10 (1:1000, MA1-81358, Thermo Fisher Scientific), anti-AXIN2 (1:100, 76G6, Cell Signaling Technology) and secondary antibodies in blocking solution for 1 h, respectively, and DAPI-stained to visualise DNA. DAPI staining without permeabilisation was performed for HeLa cells expressing mCitrine and mCherry fusion proteins. Coverlips were mounted on glass slides using fluorescent mounting media (DAKO). Cells were imaged on an LSM710 confocal laser scanning microscope (Zeiss). Micrographs were acquired in the Zen software (Zeiss) and channels separated using Adobe Photoshop. A uniform exposure adjustment across all panels was performed for Figures 6B and S7C and D to enhance visibility of localisation features in the figures.

Supplemental References

Adams, P.D., Afonine, P.V., Bunkóczi, G., Chen, V.B., Davis, I.W., Echols, N., Headd, J.J., Hung, L.-W., Kapral, G.J., Grosse-Kunstleve, R.W., et al. (2010). PHENIX: a comprehensive Python-based system for macromolecular structure solution. Acta Crystallogr. D Biol. Crystallogr. *66*, 213–221.

Alvarez-Gonzalez, R., and Jacobson, M.K. (1987). Characterization of polymers of adenosine diphosphate ribose generated in vitro and in vivo. Biochemistry *26*, 3218–3224.

Alvarez-Gonzalez, R., and Jacobson, M.K. (2011). Quantification of poly(ADP-ribose) in vitro: determination of the ADP-ribose chain length and branching pattern. Methods Mol. Biol. *780*, 35–46.

Arnold, K., Bordoli, L., Kopp, J., and Schwede, T. (2006). The SWISS-MODEL workspace: a web-based environment for protein structure homology modelling. Bioinformatics *22*, 195–201.

Collaborative Computational Project, N.4. (1994). The CCP4 suite: programs for protein crystallography. Acta Crystallogr. D Biol. Crystallogr. *50*, 760–763.

Colwill, K., Wells, C., Elder, K., Goudreault, M., Hersi, K., Kulkarni, S., Hardy, W.R., Pawson, T., and Morin, G. (2006). Modification of the Creator recombination system for proteomics applications – improved expression by addition of splice sites. BMC Biotechnol *6*, 13.

Emsley, P., Lohkamp, B., Scott, W.G., and Cowtan, K. (2010). Features and development of Coot. Acta Cryst (2010). D66, 486-501 [Doi:10.1107/S0907444910007493] 1–16.

Foadi, J., Aller, P., Alguel, Y., Cameron, A., Axford, D., Owen, R.L., Armour, W., Waterman, D.G., Iwata, S., and Evans, G. (2013). Clustering procedures for the optimal selection of data sets from multiple crystals in macromolecular crystallography. Acta Crystallogr. D Biol. Crystallogr. *69*, 1617–1632.

Francis, N.J., Saurin, A.J., Shao, Z., and Kingston, R.E. (2001). Reconstitution of a functional core polycomb repressive complex. Molecular Cell *8*, 545–556.

Gasteiger, E., Hoogland, C., Gattiker, A., and Wilkins, M.R. (2005). Protein identification and analysis tools on the ExPASy server (Totowa, NJ: Humana Press).

Guettler, S., LaRose, J., Petsalaki, E., Gish, G., Scotter, A., Pawson, T., Rottapel, R., and Sicheri, F. (2011). Structural basis and sequence rules for substrate recognition by Tankyrase explain the basis for cherubism disease. Cell *147*, 1340–1354.

Kabsch, W. (2010). XDS. Acta Crystallogr. D Biol. Crystallogr. *66*, 125–132.

Karplus, P.A., and Diederichs, K. (2012). Linking crystallographic model and data quality. Science *336*, 1030–1033.

Kim, C.A., Gingery, M., Pilpa, R.M., and Bowie, J.U. (2002). The SAM domain of polyhomeotic forms a helical polymer. Nature Structural & Molecular Biology *9*, 453–457.

Krissinel, E., and Henrick, K. (2007). Inference of macromolecular assemblies from crystalline state.

Larkin, M.A., Blackshields, G., Brown, N.P., and Chenna, R. (2007). Clustal W and Clustal X

version 2.0.

Lees, J.G., Smith, B.R., Wien, F., Miles, A.J., and Wallace, B.A. (2004). CDtool-an integrated software package for circular dichroism spectroscopic data processing, analysis, and archiving. Analytical Biochemistry *332*, 285–289.

Li, M.Z., and Elledge, S.J. (2007). Harnessing homologous recombination in vitro to generate recombinant DNA via SLIC. Nat Meth *4*, 251–256.

McCoy, A.J., Grosse-Kunstleve, R.W., Adams, P.D., Winn, M.D., Storoni, L.C., and Read, R.J. (2007). Phaser crystallographic software. J Appl Crystallogr *40*, 658–674.

Panzeter, P.L., and Althaus, F.R. (1990). High resolution size analysis of ADP-ribose polymers using modified DNA sequencing gels. Nucleic Acids Research *18*, 2194.

Patke, S., Maheshwari, R., Litt, J., Srinivasan, S., Aguilera, J.J., Colón, W., and Kane, R.S. (2012). Influence of the carboxy terminus of serum amyloid A on protein oligomerization, misfolding, and fibril formation. Biochemistry *51*, 3092–3099.

Pettersen, E.F., Goddard, T.D., Huang, C.C., Couch, G.S., Greenblatt, D.M., Meng, E.C., and Ferrin, T.E. (2004). UCSF Chimera--a visualization system for exploratory research and analysis. J Comput Chem *25*, 1605–1612.

Šali, A., Potterton, L., and Yuan, F. (1995). Evaluation of comparative protein modeling by MODELLER. Proteins: Structure.

Veeman, M.T., Slusarski, D.C., Kaykas, A., Louie, S.H., and Moon, R.T. (2003). Zebrafish prickle, a modulator of noncanonical Wnt/Fz signaling, regulates gastrulation movements. Curr. Biol. *13*, 680–685.

Waterhouse, A.M., Procter, J.B., Martin, D.M.A., Clamp, M., and Barton, G.J. (2009). Jalview Version 2--a multiple sequence alignment editor and analysis workbench. Bioinformatics *25*, 1189–1191.

Whitmore, L., and Wallace, B.A. (2004). DICHROWEB, an online server for protein secondary structure analyses from circular dichroism spectroscopic data. Nucleic Acids Research *32*, W668–W673.

Whitmore, L., and Wallace, B.A. (2008). Protein secondary structure analyses from circular dichroism spectroscopy: Methods and reference databases. Biopolymers *89*, 392–400.

Winn, M.D., Ballard, C.C., Cowtan, K.D., Dodson, E.J., Emsley, P., Evans, P.R., Keegan, R.M., Krissinel, E.B., Leslie, A.G.W., McCoy, A., et al. (2011). Overview of the CCP4 suite and current developments. Acta Crystallogr. D Biol. Crystallogr. *67*, 235–242.
