# Supplementary material for: Tankyrase Requires SAM Domain-Dependent Polymerization to Support Wnt-β-Catenin Signaling
Source: Mol Cell. 2016 Aug 4;63(3):498–513. doi: 10.1016/j.molcel.2016.06.019 (PMC4980433; doi:10.1016/j.molcel.2016.06.019)
Supplement: Document S2. Article plus Supplemental Information [file mmc2.pdf]

Article

Molecular Cell

Tankyrase Requires SAM Domain-Dependent Polymerization to Support Wnt-β-Catenin Signaling

Graphical Abstract

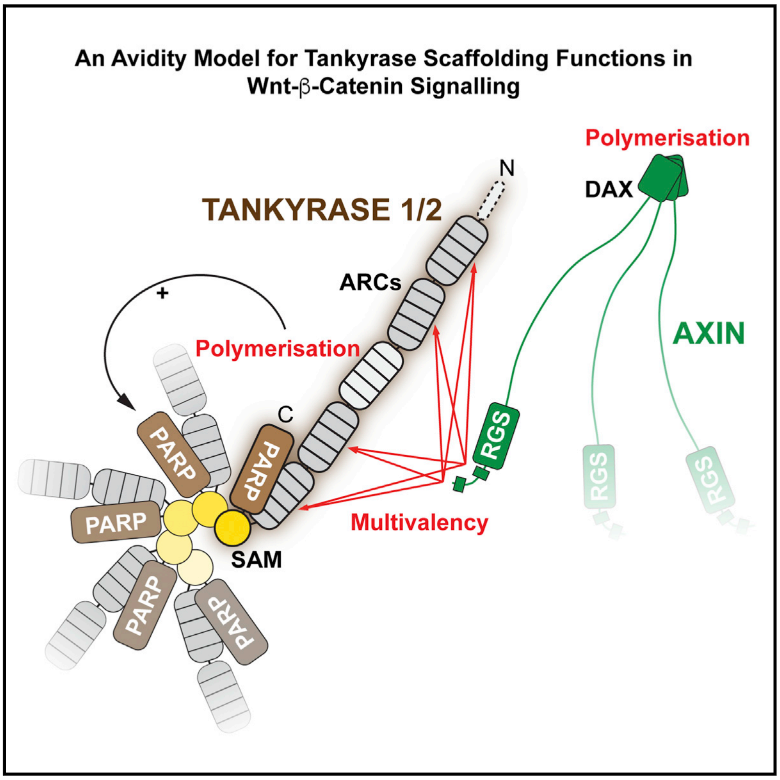

Authors

Laura Mariotti,
Catherine M. Templeton,
Michael Ranes, ..., Fabienne Beuron,
Edward Morris, Sebastian Guettler

Correspondence

sebastian.guettler@icr.ac.uk

In Brief

Catalysis-independent scaffolding by Tankyrase supports Wnt-β-catenin signaling. Scaffolding is mediated by AXIN-binding ankyrin repeat clusters and a polymerizing SAM domain. In a structure-function approach, Mariotti et al. show that polymerization supports Tankyrase PARP activity and enables avidity-dependent AXIN binding.

Highlights

- SAM domain crystal structures reveal mechanism of Tankyrase polymerization
- Catalysis-independent Tankyrase scaffolding drives Wnt-β-catenin signaling
- Tankyrase polymerization supports PARP activity and AXIN binding

Accession Numbers

5JRT
5JU5
5JTI

Mariotti et al., 2016, Molecular Cell *63*, 498–513
August 4, 2016 © 2016 The Author(s). Published by Elsevier Inc.
http://dx.doi.org/10.1016/j.molcel.2016.06.019

Cell Press
Molecular Cell
Article

Tankyrase Requires SAM Domain-Dependent Polymerization to Support Wnt-β-Catenin Signaling

Laura Mariotti,[1,2,3] Catherine M. Templeton,[1,2,3] Michael Ranes,[1,2,3] Patricia Paracuellos,[1,2,4] Nora Cronin,[1] Fabienne Beuron,[1] Edward Morris,[1] and Sebastian Guettler[1,2,*]
[1]Division of Structural Biology
[2]Division of Cancer Biology
The Institute of Cancer Research (ICR), London SW7 3RP, UK
[3]Co-first author
[4]Present address: Department of Life Sciences, Imperial College London, London SW7 2AZ, UK
*Correspondence: sebastian.guettler@icr.ac.uk
http://dx.doi.org/10.1016/j.molcel.2016.06.019

SUMMARY

The poly(ADP-ribose) polymerase (PARP) Tankyrase (TNKS and TNKS2) is paramount to Wnt-β-catenin signaling and a promising therapeutic target in Wnt-dependent cancers. The pool of active β-catenin is normally limited by destruction complexes, whose assembly depends on the polymeric master scaffolding protein AXIN. Tankyrase, which poly(ADP-ribosyl)ates and thereby destabilizes AXIN, also can polymerize, but the relevance of these polymers has remained unclear. We report crystal structures of the polymerizing TNKS and TNKS2 sterile alpha motif (SAM) domains, revealing versatile head-to-tail interactions. Biochemical studies informed by these structures demonstrate that polymerization is required for Tankyrase to drive β-catenin-dependent transcription. We show that the polymeric state supports PARP activity and allows Tankyrase to effectively access destruction complexes through enabling avidity-dependent AXIN binding. This study provides an example for regulated signal transduction in non-membrane-enclosed compartments (signalosomes), and it points to novel potential strategies to inhibit Tankyrase function in oncogenic Wnt signaling.

INTRODUCTION

Signal transduction often occurs through large and transient multi-protein complexes. Polymerizing proteins can nucleate the assembly of higher-order structures termed signalosomes, which enable locally increased protein concentrations for efficient, transient, and spatially confined processes (Bienz, 2014; Wu, 2013). Wnt-β-catenin signaling, which is dysregulated in most colorectal cancers, provides prominent examples for signalosomes (Bienz, 2014; Polakis, 2012). At basal signaling, a destruction complex (DC) composed of the scaffolding proteins AXIN and adenomatous polyposis coli (APC), glycogen synthase kinase 3 (GSK3), and casein kinase 1 (CK1) captures and phosphorylates β-catenin to initiate its degradation (Stamos and Weis, 2013). AXIN is the central and concentration-limiting component of the DC (Lee et al., 2003; Stamos and Weis, 2013). Microscopically, DCs manifest as dynamic puncta with a filamentous sub-organization, so-called β-catenin degradasomes, whose formation is dependent on AXIN polymerization (Fiedler et al., 2011; de la Roche et al., 2014; Martino-Echarri et al., 2016; Thorvaldsen et al., 2015).

The poly(ADP-ribose) polymerases (PARPs) Tankyrase (TNKS and ARTD5) and Tankyrase 2 (TNKS2 and ARTD6) regulate Wnt-β-catenin signaling (Huang et al., 2009). We shall refer to TNKS and TNKS2 collectively as Tankyrase where principles apply to both. Tankyrase binds and poly(ADP-ribosyl)ates (PARylates) AXIN, targeting it for PAR-dependent ubiquitination (PARdU) and degradation to stabilize transcriptionally active β-catenin (Callow et al., 2011; DaRosa et al., 2015; Huang et al., 2009; Morrone et al., 2012; Zhang et al., 2011). Tankyrase buffers negative regulation of Wnt signaling by AXIN for robust pathway activation (Wang et al., 2016). Upon Wnt stimulation, AXIN PARylation by Tankyrase promotes its function in active signaling complexes (Yang et al., 2016).

The two Tankyrases are highly similar (Hsiao and Smith, 2008; Smith et al., 1998) (Figure 1A), sharing a set of five ankyrin repeat clusters (ARCs) for substrate binding (Guettler et al., 2011; Seimiya et al., 2004), a sterile alpha motif (SAM) domain (De Rycker and Price, 2004; De Rycker et al., 2003), and a catalytic PARP domain (Rippmann et al., 2002). Tankyrase's biological functions are complex (Haikarainen et al., 2014), and simultaneous loss of both Tankyrases results in embryonic lethality in mice (Chiang et al., 2008). Tankyrase contributes to telomere maintenance (Canudas et al., 2007; Dynek and Smith, 2004; Smith et al., 1998), which together with Wnt signaling is relevant to stem cell renewal, development, and certain types of cancer (Bernardes de Jesus and Blasco, 2013; Clevers et al., 2014). Given these functions and a dependency of BRCA1/2-deficient cancer cells on Tankyrase (McCabe et al., 2009), Tankyrase is a promising anti-cancer target (Haikarainen et al., 2014; Lehtiö et al., 2013; Riffell et al., 2012).

It is intriguing that Tankyrase, like AXIN, polymerizes (De Rycker and Price, 2004; De Rycker et al., 2003). Tankyrase polymerization is mediated by the SAM domain, a small helical fold highly prevalent in eukaryotes (Knight et al., 2011; Qiao and

 © 2016 The Author(s). Published by Elsevier Inc.
This is an open access article under the CC BY license (http://creativecommons.org/licenses/by/4.0/).

CellPress

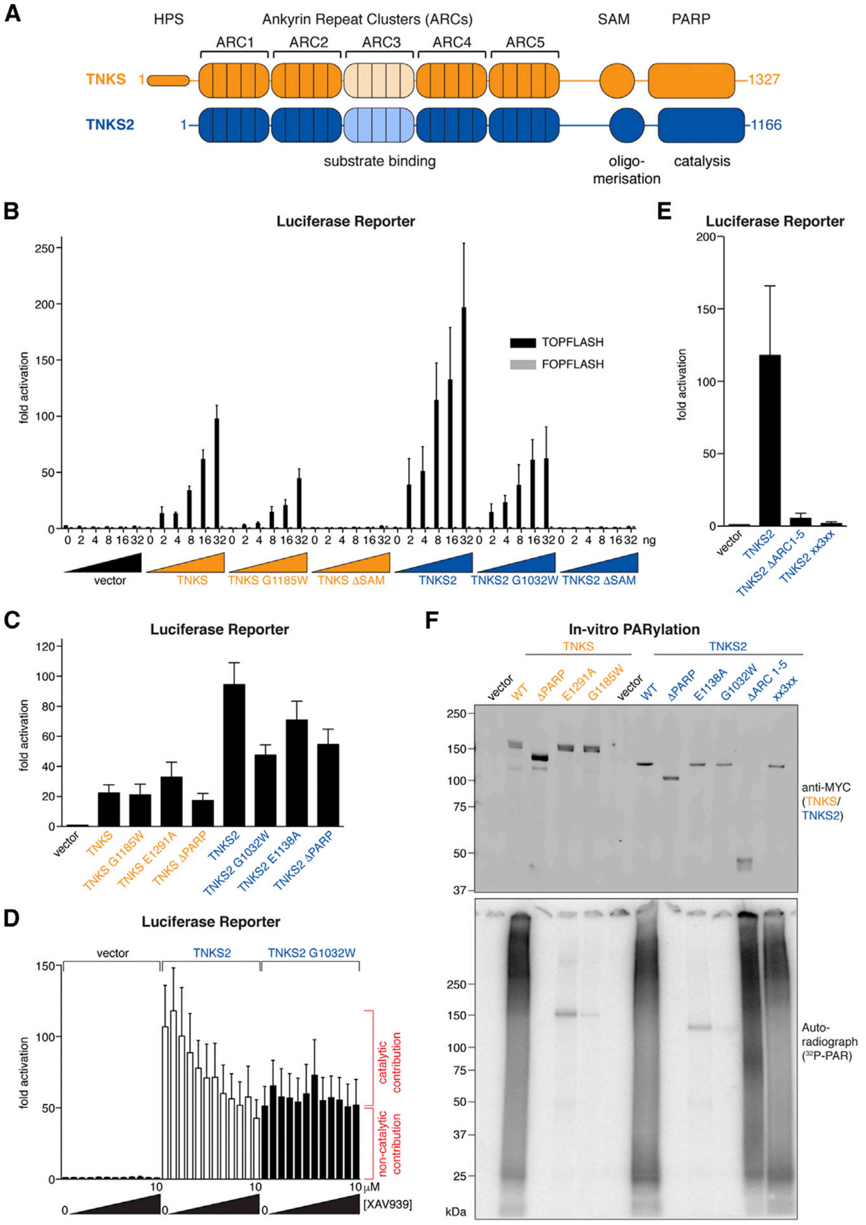

Figure 1. Requirement of ARCs and SAM Domains for Tankyrase-Driven Wnt Signaling

(A) Domains of human TNKS and TNKS2 are shown.

(B) Activation of β-catenin/TCF/LEF-dependent transcription by MYC_2-Tankyrases in unstimulated HEK293T cells, assayed by TOPFlash and control FOPFlash reporters. Data are expressed relative to mean reporter activities obtained without MYC_2 construct (seven samples in set; n = 3 duplicate experiments; error bars, SEM).

(C) Transcription reporter assay as in (B), using 16 ng of MYC_2-Tankyrase constructs. Fold activation is relative to vector only (n = 6 duplicate experiments; error bars, SEM).

(D) Transcription reporter assay as in (C). Cells were treated with 9.8 nM to 10 μM XAV939 in a 2-fold dilution series. Data are expressed relative to reporter activity in the vector control in the absence of XAV939 (n = 3 duplicate experiments; error bars, SEM). See Figure S2A for TNKS2 PARylation assessment.

(E) Transcription reporter assay as in (C) (n = 3 duplicate experiments; error bars, SEM). See Figure S1 for Tankyrase expression levels in luciferase reporter assays.

(F) In vitro PARylation assay for the indicated immunoprecipitated MYC_2-tagged Tankyrases. Top: western blot analysis of immunoprecipitates is shown; and bottom: autoradiograph is shown.

Bowie, 2005). The structural basis of Tankyrase polymerization and its function have remained unknown. Moreover, we currently lack insight into the regulation of Tankyrase activity.

Here we show that Tankyrase can induce Wnt-β-catenin signaling independently of its catalytic activity, through ARC- and SAM domain-dependent scaffolding. This redefines our view on pharmacologic inhibition of Tankyrase. Informed by crystal structures of the TNKS and TNKS2 SAM domains, we demonstrate that Tankyrase polymerization is critical for its function in Wnt signaling, required for full PARP activity, and necessary for efficient interaction with AXIN. We propose a model in which recruitment of Tankyrase to β-catenin DCs is promoted by avidity effects that arise from multivalency and polymerization inherent to the Tankyrase-AXIN complex.

RESULTS

Tankyrase Requires ARCs and SAM Domain to Promote Wnt Signaling

To explore the domain requirements of Tankyrase for Wnt-β-catenin signaling, we measured β-catenin/TCF/LEF-dependent transcription in reporter assays. While basal Wnt signaling in HEK293T cells is low (Li et al., 2012), expression of TNKS or TNKS2 activated the reporter in a specific, dose-dependent manner (Figure 1B; see Figure S1 for protein expression levels). Intriguingly, reporter activation by either TNKS or TNKS2 was not abolished but merely reduced by ≈50% when poly- and mono(ADP-ribosyl)ation were inactivated by point mutation ($G1185W^{T1}$ and $G1032W^{T2}$; Figures 1B and 1F) (Yu et al., 2005). Likewise, mutation of a glutamate that is part of the catalytic H-Y-E triad ($E1291A^{T1}$ and $E1138A^{T2}$) (Hottiger et al., 2010), or deletion of the PARP domain altogether, did not abolish reporter activation (Figures 1C and 1F). Concordantly, saturating concentrations of the Tankyrase inhibitor XAV939 reduced TNKS2-dependent reporter activation only to a level that also was conferred by PARP-inactive TNKS2 $G1032W^{T2}$ (Figures 1D and S2A). This suggests that both catalysis-dependent and -independent functions of Tankyrase contribute to Wnt signaling.

We hypothesized that scaffolding through the SAM domain and ARCs contributes to signaling. Deletion of the SAM domain

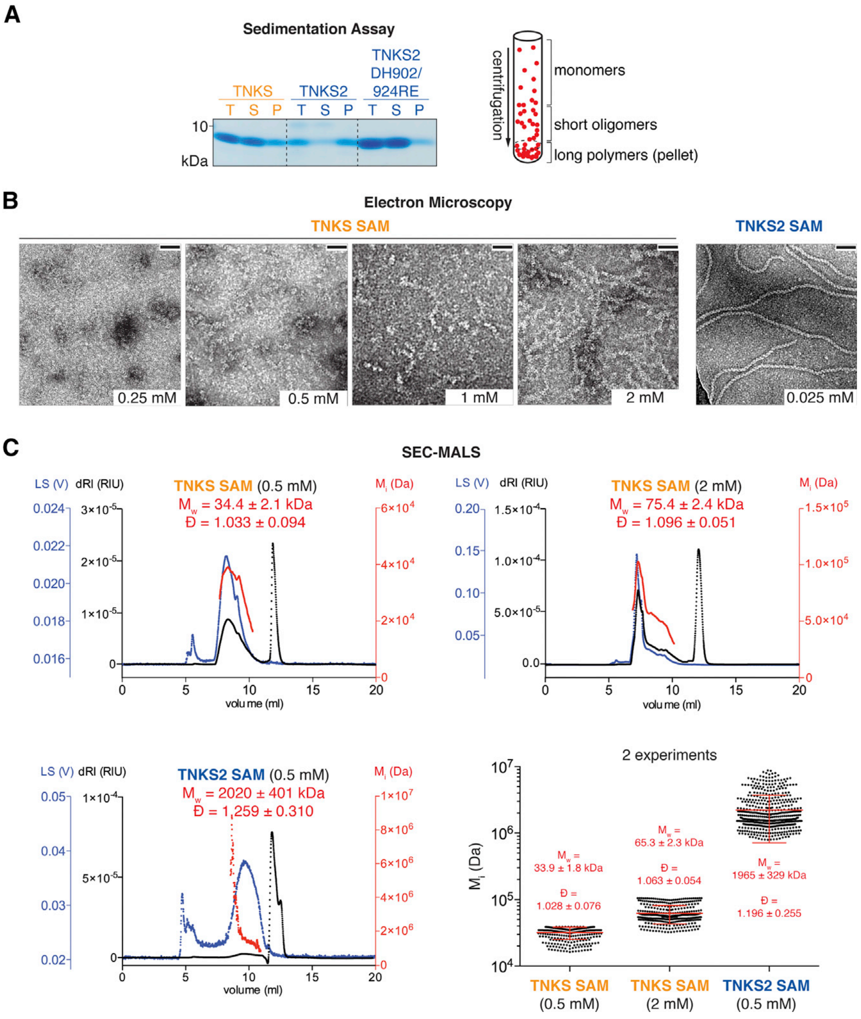

Figure 2. Polymerization of the TNKS and TNKS2 SAM Domains

(A) Ultracentrifugation sedimentation assay. Purified SAM domains (25 μM) were centrifuged and total samples (T), supernatants (S), and pellets (P) were analyzed by SDS-PAGE and Coomassie staining. The diagram illustrates the assay principle.

(B) Electron micrographs of SAM domains at the indicated concentrations are shown. Scale bars, 50 nm.

(C) SEC-MALS. Chromatograms show one experiment with differential refractive index (dRI), light scattering (LS), and calculated molecular weight per slice i (M_i). Weight-average molecular weights (M_w) and dispersity (Đ) ± SD over peaks are indicated. See Figures S3B and S3C for eluate analyses by SDS-PAGE. The atypically delayed elution of the long TNKS2 SAM filaments likely reflects an interaction/entanglement with the column solid phase. Scatterplots combine data from two experiments with M_w, Đ, and associated SD indicated. Plotted data points with mean and error bars (SD) refer to M_i. See Figures S2 and S3 for further data.

fully abrogated Tankyrase-dependent reporter activation (Figure 1B), as did deletion of all ARCs or mutation of ARCs 1, 2, 4, and 5 (mutant xx3xx) to prevent substrate binding (Guettler et al., 2011) without impairing catalysis (Figures 1E and 1F). Our observations expand upon and are in line with previous deletion studies (Huang et al., 2009). ARCs and the SAM domain may collaborate in recruiting Tankyrase to AXIN and/or facilitate productive PARylation. Overexpression of Tankyrase-binding-deficient, but not wild-type (WT), AXIN1 impeded TNKS2-dependent Wnt signaling (Figure S2B). This is in agreement with Tankyrase activating Wnt-β-catenin signaling at the level of AXIN, and it illustrates the strong buffering capacity of Tankyrase toward AXIN (Huang et al., 2009; Wang et al., 2016).

Polymerization of TNKS and TNKS2 SAM Domains

AXIN binding by the Tankyrase ARCs is well understood (Guettler et al., 2011; Huang et al., 2009; Morrone et al., 2012). Conversely, it remains unclear how the SAM domain enables Tankyrase function in Wnt signaling and whether polymerization is involved. To study SAM domain polymerization, we performed ultracentrifugation sedimentation assays, in which polymers of purified SAM domains partition into the pellet (Figure 2A). While the TNKS2 SAM domain readily sedimented, that of TNKS did not, suggesting that TNKS SAM forms less stable polymers in vitro (Figure 2A). We observed filaments for both the TNKS2 and TNKS SAM domains by electron microscopy (EM), but TNKS SAM required higher concentrations to form visible filaments (Figure 2B). Based on a homology model (not shown), we generated a DH902/924RE[T2] mutant of the TNKS2 SAM domain, which failed to sediment (Figure 2A).

We next analyzed highly purified SAM domains by size exclusion chromatography with in-line multi-angle light scattering (SEC-MALS), which is more sensitive than the sedimentation assay. When analyzed at 0.5 mM, the TNKS2 SAM domain (theoretical molecular weight [MW] ≈9 kDa) eluted as polydisperse assemblies of overall 1,965 ± 329 kDa, clearly indicating polymerization (Figure 2C). For 0.5 and 2 mM TNKS SAM, we detected polydisperse assemblies of 33.9 ± 1.8 kDa and 65.3 ± 2.3 kDa, respectively (Figure 2C), confirming that TNKS SAM also polymerizes, although to a lesser extent. We found that differential polymerization of the TNKS and TNKS2 SAM domains is largely due to a single amino acid difference (T1049[T1] and R896[T2], Figures S2C, S2D, S3A, and S3B). However, transcription reporter assays with TNKS/TNKS2 SAM domain chimeras and interconverting point mutants (T1049R[T1] and R896T[T2]) showed that both SAM domains equally enable Tankyrase to drive Wnt signaling (Figure S2E). Thus, the SAM domain may either promote Tankyrase function independently of its polymerization, or the lower polymerization of TNKS may still be sufficient for Wnt signaling under the assay conditions. In the latter case, a substantial impairment of

Table 1. Data Collection and Refinement Statistics

Data Collection[a]	TNKS2 SAM DH902/924RE	TNKS SAM D1055R Crystal Form 1 (Five Datasets/Three Crystals)	TNKS SAM D1055R Crystal Form 2 (Two Datasets/Two Crystals)
PDB ID	5JRT	5JU5	5JTI
Beamline	Diamond I03	Diamond I03	Diamond I03
Wavelength (Å)	0.976	0.976	0.976
Space group	$P6_5$	$P2_1$	$P2_1$
Unit cell			
a, b, c (Å)	56.63, 56.63, 46.11	52.24, 55.22, 83.05	70.93, 55.48, 79.41
α, β, γ (°)	90, 90, 120	90, 96.2, 90	90, 102.9, 90
Molecules/ASU	1	6	6
Resolution (Å)	28.32–1.53 (1.56–1.53)	82.57–2.5(2.6–2.5)	77.41–2.9 (3.0–2.9)
Total number of reflections	207,561 (10,406)	454,569 (51,435)	87,050 (13,527)
Number of unique reflections	12,797 (618)	16,511 (1,870)	13,604 (2,185)
R_{merge}[b]	0.058 (2.932)	0.406 (5.731)	2.297 (6.557)
R_{meas}[b]	0.061 (3.120)	0.422 (6.002)	2.525 (7.589)
Mean I/σI	20.5 (0.9)	11.2 (1.3)	9.6 (1.4)
$CC_{1/2}$[c]	0.999 (0.408)	0.997 (0.333)	0.892 (0.35)
CC:d1		0.996 (0.030)	0.969 (0.494)
CC:d2		0.998 (0.713)	0.817 (0.256)
CC:d12	0.99 (0.323)		
CC:d3	0.99 (0.480)	0.999 (0.683)	0.973 (0.678)
Completeness (%)	100 (100)	100 (100)	100 (100)
Multiplicity	16.2 (16.7)	27.5 (27.5)	6.6 (6.5)
Wilson B factor (Å)[b]	37.69	34.51	18.57
**Refinement**[a]			
Resolution (Å)	28.32–1.53	82.57–2.5	77.41–2.9
R_{work}/R_{free} (test set 5%)	0.201/0.233	0.191/0.211	0.193/0.232
Reflections used in refinement	12,770	16,498	13,593
Reflections in R_{free} test set	636	802	681
RMSD bond lengths (Å)	0.01	0.01	0.01
RMSD bond angles (°)	0.94	1.13	1.19
Number of protein atoms	510	2,855	2,854
Number of solvent atoms	40	24	62
B factor protein (Å)[b]	46.71	65.21	53.1
B factor solvent (Å)[b]	50.92	52.81	36.54
Ramachandran favored (%)	100	99	96.5
Ramachandran allowed (%)	0	1	3.5
Ramachandran disallowed (%)	0	0	0

[a]Values for the highest-resolution shell are shown in parentheses.
[b]R_{merge} and R_{meas} are as calculated in AIMLESS (Winn et al., 2011). High R_{merge} and R_{meas} are attributable to the high-resolution cutoff (Karplus and Diederichs, 2012), multi-dataset merging, and anisotropy (see next footnote).
[c]The principal directions of anisotropy are as defined by symmetry (axes or planes), as analyzed in AIMLESS (Winn et al., 2011). For TNKS SAM crystal form 1, the anisotropy is pronounced along CC_d1 (along 0.91 hr–0.40 l), with $CC_{1/2}$ falling below 0.30 at 3.0 Å.

TNKS/TNKS2 polymerization would abrogate Tankyrase-dependent Wnt signaling.

Crystal Structures of TNKS2 and TNKS SAM Domains

To enable the generation of well-defined Tankyrase mutants, we crystallized the TNKS2 SAM domain. Since polymerization was likely to hinder crystallization, we used the polymerization-impaired DH902/924RE[T2] mutant. Reasoning that the mutant domain would still retain most polymerization interface residues, polymer contacts would be recoverable at the high protein concentration during crystallization, as illustrated for other polymerizing SAM domains (Kim et al., 2001, 2002). We obtained well-diffracting crystals (1.53 Å) and solved the structure by molecular replacement (Table 1; Supplemental Experimental

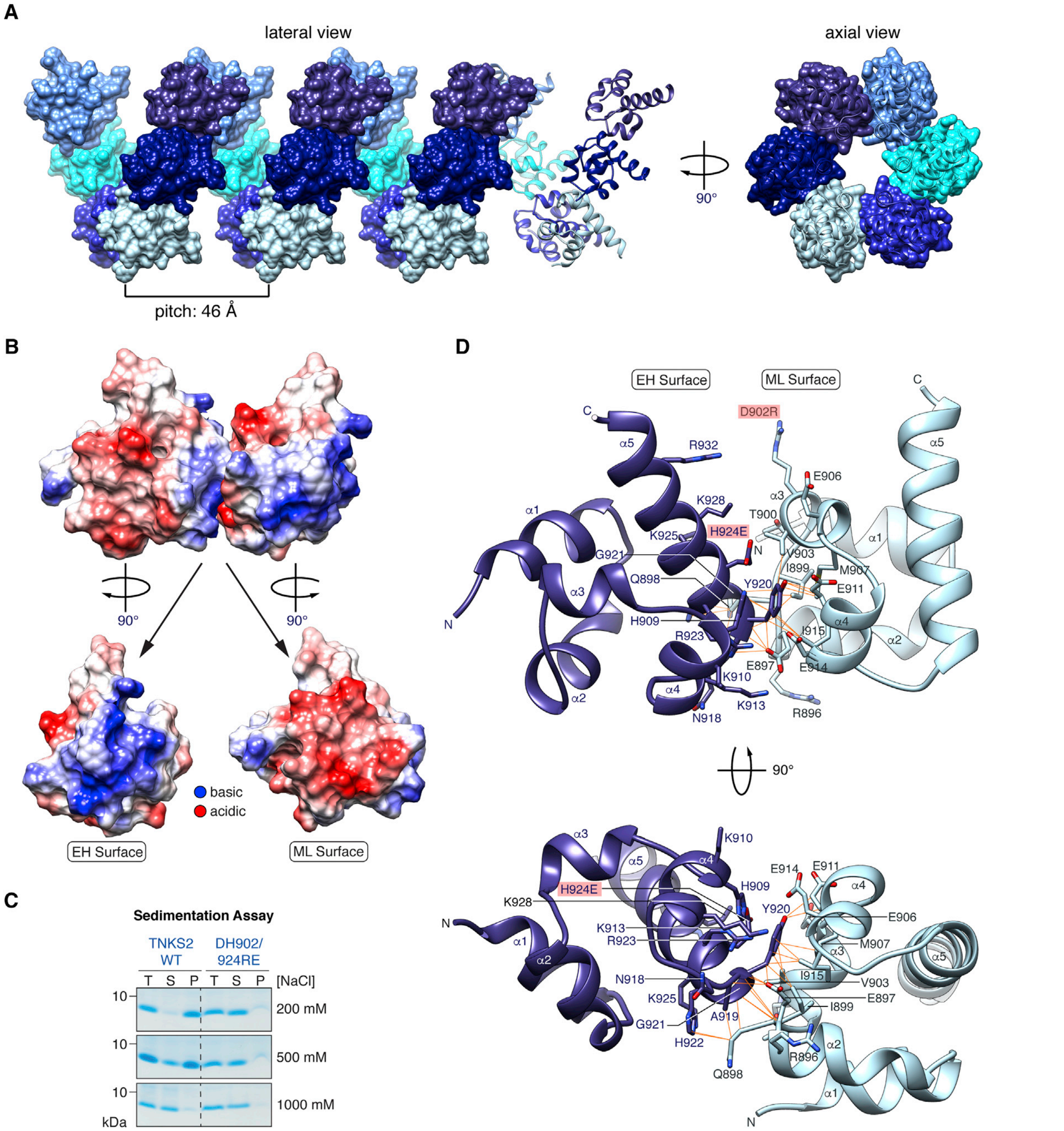

Figure 3. Crystal Structure of the TNKS2 SAM Domain

(A) A structural representation of the TNKS2 DH902/924RE^{T2} SAM domain filament is shown.

(B) A pair of WT-rendered TNKS2 SAM domains from the filament, colored by Coulombic surface electrostatic potential, is shown.

(C) Ultracentrifugation sedimentation assay as for Figure 2A at increasing [NaCl] is shown.

(D) Detailed representation of a TNKS2 DH902/924RE^{T2} SAM domain pair. Interface residues are in stick representation with orange lines indicating contacts. Mutations required for crystallization are indicated.

Procedures). The TNKS2 SAM domain, a 5-α-helix bundle similar to other SAM domains, formed a left-handed helix with a pitch of 46 Å, whose axis coincided with the crystallographic $P6_5$ screw axis (Figure 3A). The SAM domains interacted in the well-established end-helix (EH)-mid-loop (ML) binding mode (Qiao and Bowie, 2005) (Figures 3B and 3D). On the EH surface, basic electrostatic potential predominated while the ML surface was predominantly acidic, in line with the salt sensitivity of the polymer (Figures 3B and 3C). The closest approach between the two surfaces occurred around the N terminus of helix α5, where EH

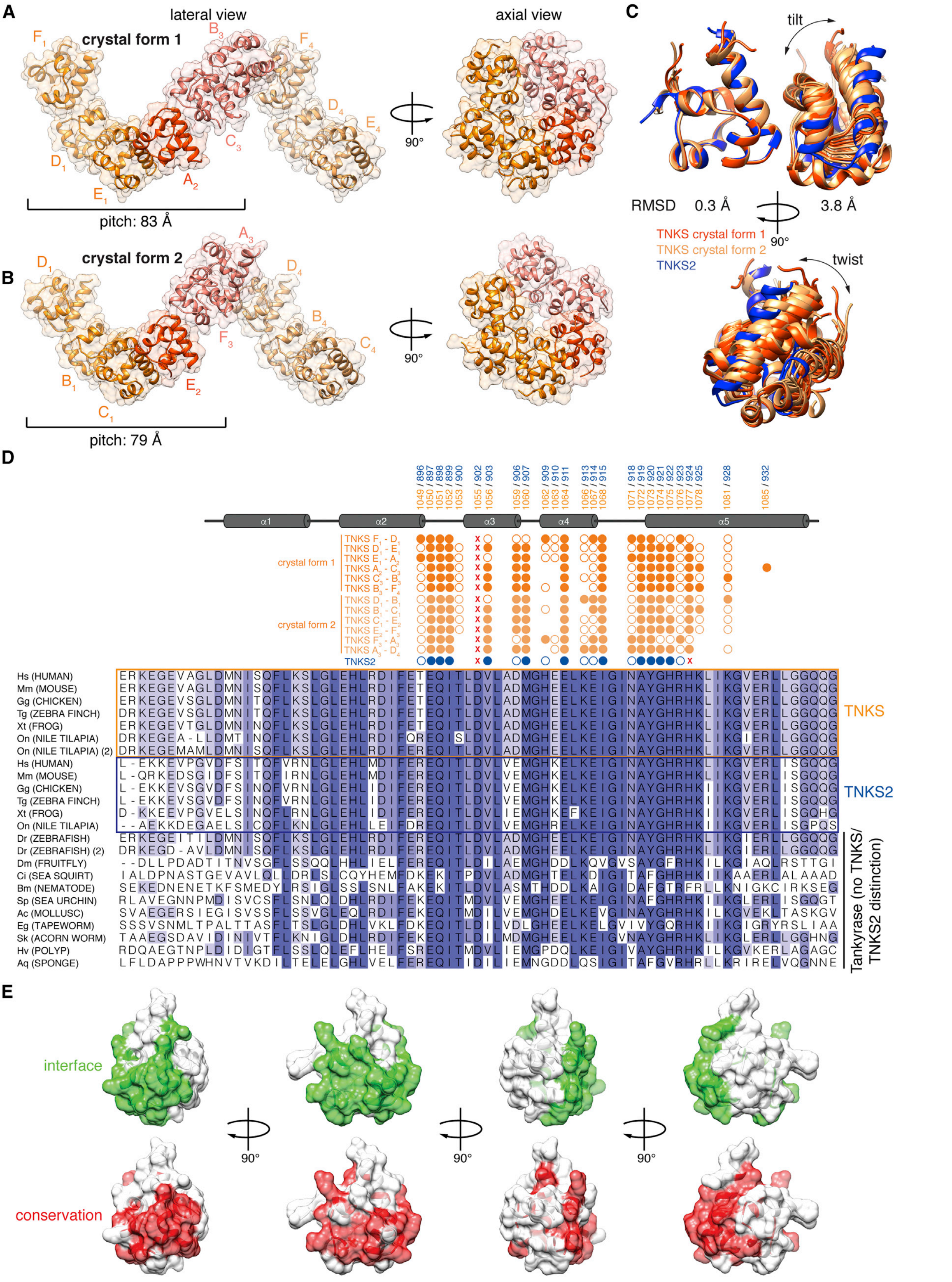

(legend on next page)

surface residues A919^{T2}, Y920^{T2}, G921^{T2}, and H922^{T2} engaged in a network of hydrogen bonds and van der Waals contacts (Figure 3D). H922^{T2} and A919^{T2} contacted the Q898^{T2} side chain. Y920^{T2} was the most buried side chain at the interface (125 Å^2), interacting with various hydrophobic ML side chains (V903^{T2}, I899^{T2}, I915^{T2}, and M907^{T2}), which collectively formed a shallow pocket, as well as E911^{T2} and E897^{T2}. In turn, E897^{T2} bound the protein main chain at A919^{T2} and Y920^{T2}. The main chains of adjacent SAM domains interacted between G921^{T2} and E897^{T2}/Q898^{T2}. The interface opened up toward the outside of the filament. In its non-mutated form, D902^{T2} likely forms a salt bridge with R932^{T2}, which may promote another salt bridge between K928^{T2} and E906^{T2}. Surprisingly, despite its importance for TNKS2 SAM domain polymerization, R896^{T2} was not involved in any contact (Figure 3D).

We also crystallized the TNKS SAM domain, which again required a polymer-breaking mutation. TNKS SAM D1055R^{T1}, equivalent to D902R^{T2}, produced two crystal forms in space group P2$_1$, diffracting to 2.5 Å (crystal form 1) and 2.9 Å (crystal form 2), both with six molecules in distinct asymmetric units (Table 1; Figures 4A, 4B, and S4). The TNKS SAM domain was highly similar to that of TNKS2 (Figure 4C, left). For both TNKS crystal forms, non-crystallographic and crystallographic symmetry gave rise to left-handed helical filaments established by EH-ML contacts (Figures 4A, 4B, and S4). The repeating unit consisted of six SAM domains with pitches of 83 and 79 Å, almost twice as long as for TNKS2 (Figures 4A and 4B). Unlike for TNKS2, where protomer contacts relied on crystallographic symmetry only and were therefore uniform, the TNKS SAM EH-ML contacts varied substantially. This was apparent from the merely approximate 6-fold axial symmetry and the variable tilt and twist between adjacent SAM domains (Figures S4 and 4C). The three crystal structures provided snapshots of 13 unique SAM domain pairs. Many contacts were shared between all EH-ML interactions, but a subset was specific to certain binding geometries, sometimes involving the same residue in alternative interactions (Figures 4D and S4C). We conjecture that the variable relative orientations of SAM domains reflect filament flexibility (Figure 2B). SAM-SAM interface residues were conserved across a wide range of phyla, including poriferans, indicating that polymerization is a common and ancient feature of Tankyrase (Figures 4D and 4E).

Characterization of Polymer Contacts by Mutagenesis

We performed site-directed mutagenesis of the TNKS2 SAM domain and assessed polymerization by ultracentrifugation sedimentation. Mutations strongly, intermediately, or weakly abrogated sedimentation (Figure 5A). In most cases, mutation of robust TNKS2 SAM contact residues (Y920^{T2}, H924^{T2}, E897^{T2}, and V903^{T2}) strongly impaired polymerization, as did mutation of E906^{T2}, K913^{T2}, and K928^{T2}. Although situated close to the SAM-SAM interface, the latter three formed no explicit contacts in the TNKS2 SAM crystal structure (Figures 3D and 4D). However, the equivalent residues (D1059^{T1}, K1066^{T1}, and K1081^{T1}, respectively) mediated binding between a subset of protomers in the TNKS SAM crystal structures (Figures 4D and S4C). Thus, contacts not seen in all SAM-SAM pairs are still generally relevant, probably occurring in some, but not all, configurations of the flexible filament.

We used SEC-MALS and EM to validate strong mutations (V903W^{T2}, E906K^{T2}, K913E^{T2}, Y920A^{T2}, H924E^{T2}, and a VY903/920WA^{T2} combination). Except for E906K^{T2} and K913E^{T2}, all mutations conferred monomeric behavior (Figures 5B and 5C). TNKS2 SAM K913E^{T2} and E906K^{T2} showed considerable residual polymerization (Figures 5B and S3F); we hence re-assigned their polymer-breaking scores to intermediate. As for TNKS2 SAM, the TNKS SAM mutations V1056W^{T1}, Y1073A^{T1}, and VY1056/1073WA^{T1} strongly abrogated polymerization (Figure 5B). Circular dichroism (CD) spectroscopy showed that the mutations did not impair SAM domain folding (Figures S5A and S5B).

Combining SAM domains with strong mutations in opposite polymerization surfaces (ML: V1056W^{T1} and V903W^{T2}; EH: Y1073A^{T1} and Y920A^{T2}) gave rise to homo- and heterotypic dimers (Figures S3D and S3E). This enabled us to assess the SAM-SAM binding affinities by isothermal titration calorimetry (ITC). TNKS and TNKS2 SAM domains bound homo- and heterotypically with comparable, low-micromolar affinities, typical for dynamic protein-protein interactions (Figures 5D and S5C; see Discussion).

Full-Length Tankyrases Interact through EH and ML SAM Domain Surfaces

We assessed self-interaction of full-length Tankyrases in co-immunoprecipitations (co-IPs) with WT Tankyrases as bait. Robust homotypic binding of TNKS and TNKS2 was abolished by SAM domain deletion or mutation of both the ML and EH surfaces (VY1056/1073WA^{T1} and VY903/920WA^{T2}), and it was reduced by mutation of either the ML surface (V1056W^{T1} and V903W^{T2}) or EH surface (Y1073A^{T1} and Y920A^{T2}) alone (Figure 6A, left and center). We also detected heterotypic binding of TNKS and TNKS2 and confirmed its sensitivity to SAM domain mutations (Figure 6A, right). The SAM domain previously was shown to confer high apparent molecular weight to TNKS in gel filtration experiments (De Rycker and Price, 2004). Using the VY1056/1073WA^{T1} and VY903/920WA^{T2} point mutants, we tested whether this reflects Tankyrase polymerization. WT TNKS and

Figure 4. Crystal Structures of the TNKS SAM Domain and Comparison with TNKS2

(A and B) Structural representations of TNKS D1055R^{T1} SAM domain filaments are shown. Subscript numbers of chain identifiers denote the corresponding asymmetric units. See Figure S4 for a contact analysis.

(C) EH-presenting SAM domains from unique SAM domain pairs were superimposed over residues 1,030–1,068^{T1}/877–933^{T2}, and average Cα root-mean-square deviation (RMSD) values for both protomers were calculated.

(D) Multiple sequence alignment of SAM domains from representative Tankyrase orthologs. Circles denote interface residues (by solvent inaccessibility); filled circles indicate explicit contacts in crystal structures. X, mutated residues. See Supplemental Experimental Procedures for sequence accession numbers.

(E) Conservation of the SAM-SAM interface. Top: interface residues observed in any of the crystal structures are in green, and bottom: residues identical in $\geq$80% of the orthologs shown in (D) are in red.

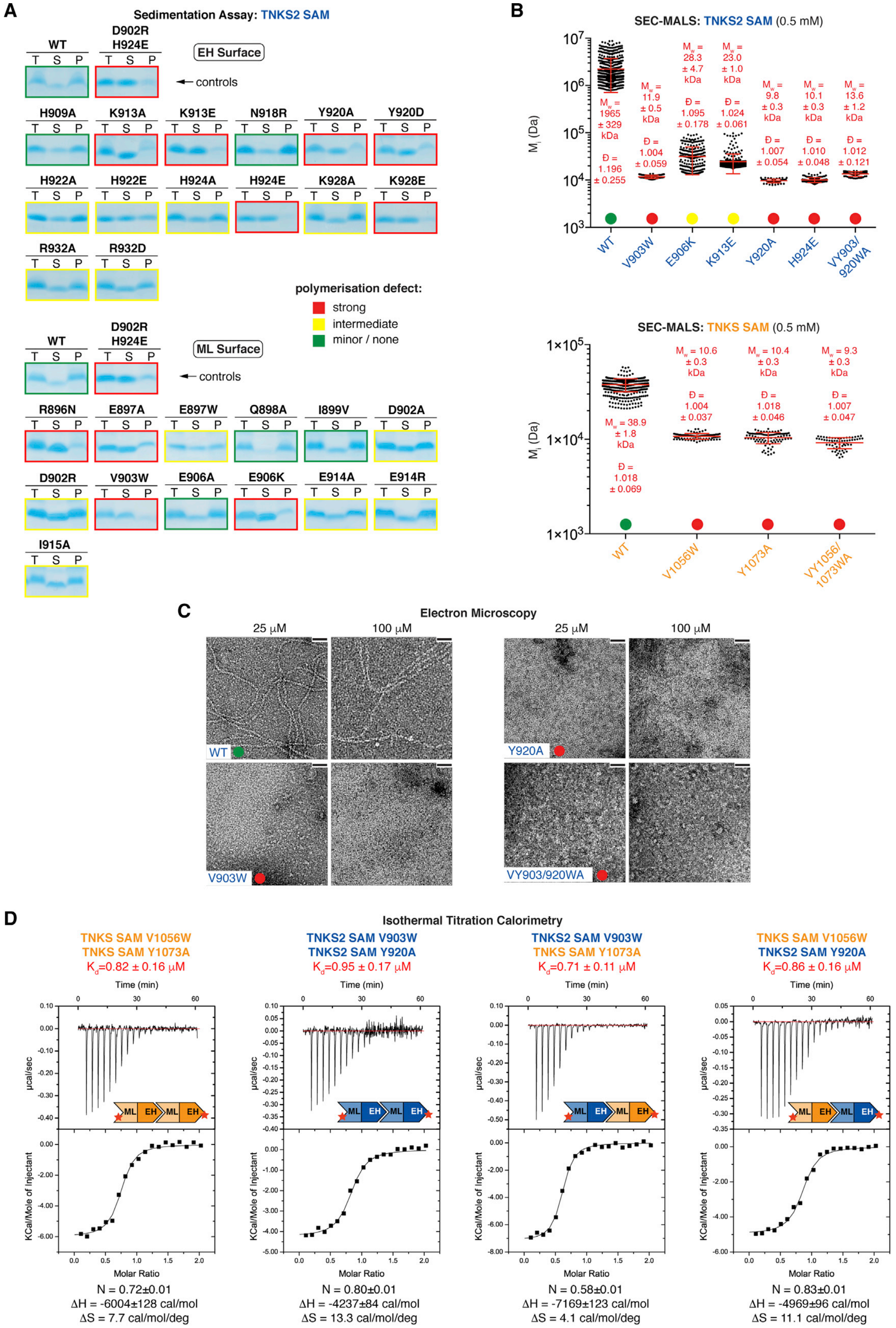

(legend on next page)

TNKS2 eluted close to the void volume with subsequent trails (Figure S6A). Both deletion and point mutation of the SAM domain resulted in an elution delay and increased trailing with an emerging late elution peak. We detected endogenous TNKS in both the early and late peaks, suggesting that TNKS exists in heterogeneous polymerization states (Figure S6A), but we were unable to detect endogenous TNKS2. Collectively, co-IP and gel filtration showed that full-length Tankyrases homo- and heteropolymerize. Using both assays, we found no evidence for modulated polymerization of full-length TNKS or TNKS2 by the T1049R^{T1} or R896T^{T2} mutations, suggesting that differential polymerization may not occur in a full-length context or only under particular conditions (Figures S6A and S6B).

Polymerization Controls Tankyrase Subcellular Localization

To address if polymerization affects Tankyrase subcellular localization, we imaged HeLa cells expressing mCitrine- and mCherry-tagged TNKS or TNKS2. Since Tankyrase PARP activity was proposed to inhibit polymerization (De Rycker and Price, 2004), we compared vehicle- and XAV939-treated cells (Figure 6B). Both mCherry-TNKS and -TNKS2 displayed a punctate, predominantly cytoplasmic distribution, with more pronounced puncta upon XAV939 treatment (Figure 6B). In contrast, co-expressed mCitrine-tagged non-polymerizing EH/ML double mutants (VY1056/1073WA^{T1} and VY903/920WA^{T2}) displayed mostly diffuse localization, even in the presence of XAV939 (Figure 6B; see Figure S6C for additional controls). This shows that polymerization enables the assembly of both TNKS and TNKS2 higher-order structures. In line with heteropolymerization, differentially tagged TNKS and TNKS2 colocalized (Figure S6D).

Polymerization Is Required for Tankyrase-Dependent Wnt Signaling

We tested how SAM domain mutations affect the ability of TNKS2 to drive Wnt signaling. We observed a correlation between the severity of the polymerization defect and diminished transcription reporter activity (Figure 6C). Likewise, strong polymer-breaking mutations abolished Wnt signaling induced by TNKS (Figure 6D). Transcription reporter assays using paired TNKS2 mutants with inactivated opposite SAM domain faces suggested that Tankyrase dimerization is insufficient to drive Wnt signaling (Figure S7A). A heterologous polymerizing SAM domain, that of *D. melanogaster* Polyhomeotic (Kim et al., 2002), only partially compensated for SAM domain loss in TNKS2; however, the partial rescue was dependent on polymerization (Figures S7B–S7E). In conclusion, SAM domain polymerization enables Tankyrase function in Wnt-β-catenin signaling.

Polymerization Promotes Tankyrase PARP Activity and Interaction with AXIN

To explore the mechanism by which Tankyrase polymerization promotes Wnt signaling, we assessed the in vitro auto-PARylation activity of immunoprecipitated MYC$_2$-TNKS2 WT, ΔSAM^{T2}, V903W^{T2}, Y920A^{T2}, and the catalytically inactive variant G1032W^{T2}. We readily observed TNKS2-dependent PARylation (Figure 7A). The ΔSAM^{T2}, V903W^{T2}, and Y920A^{T2} mutations reduced PARylation by ≈40%–50% and also accounted for strongly reduced endogenous PARylation, prior to the in vitro reaction (Figure 7A). Our observations agree with previous reports of reduced TNKS/TNKS2 activity upon SAM domain deletion (De Rycker and Price, 2004; Levaot et al., 2011), and they clarify that polymerization is required. To evaluate PARylation processivity, we detached PAR chains from the proteins and analyzed their size distribution. PAR from TNKS2 WT, V903W^{T2}, and Y920A^{T2} showed similar lengths, indicating that polymerization does not affect auto-PARylation processivity (Figure 7B). Conversely, TNKS2 ΔSAM^{T2} produced overall shorter PAR chains (Figure 7B), suggesting that the SAM domain may impact PAR chain length independently of its polymerization.

We next asked whether Tankyrase polymerization promotes its interaction with AXIN. In colorectal cancer cells, but not HeLa cells with their intact Wnt-β-catenin pathway, Tankyrase and AXIN1/2 have been shown to colocalize in β-catenin degradasomes induced by Tankyrase inhibitors (de la Roche et al., 2014; Martino-Echarri et al., 2016; Thorvaldsen et al., 2015). We hence analyzed SW480 colorectal cancer cells and observed that transiently expressed MYC$_2$-TNKS2 and endogenous AXIN2 accumulate in puncta upon XAV939 treatment (Figure 7C). Provided AXIN2 levels were sufficient for immunodetection, TNKS2 colocalized with AXIN2 in degradasomes (Figure 7C). Deletion or mutation of the SAM domain (ΔSAM^{T2} and VY903/920WA^{T2}) resulted in a more diffuse TNKS2 localization; however, we still detected substantial colocalization of these mutants with AXIN2 puncta, likely due to the interaction of the ARCs with AXIN at overexpression levels of Tankyrase. Inactivation of the ARCs (xx3xx) did not abolish puncta formation by TNKS2 but substantially reduced its colocalization with AXIN2 foci (Figure 7C). The retained colocalization may reflect residual AXIN2 binding by the xx3xx mutant and/or additional determinants, including bridging through endogenous Tankyrase. When combined with the xx3xx mutations, the ΔSAM^{T2} or VY903/920WA^{T2} mutations resulted in diffuse TNKS2 staining without colocalization in AXIN2 puncta (Figure 7C). Thus, polymerization contributes to the recruitment of TNKS2 to β-catenin degradasomes.

To more directly evaluate if Tankyrase polymerization promotes AXIN binding, we immunoprecipitated endogenous AXIN1 from

Figure 5. Characterization of Tankyrase SAM Domain Mutants

(A) Ultracentrifugation sedimentation assays as for Figure 2A. Color coding indicates the degree of abrogated sedimentation. H924^{T2}, K928^{T2}, and E906^{T2} charge reversals were more severe than changes to alanine.

(B) SEC-MALS of Tankyrase SAM domains, as in Figure 2C (M_w ± SD, Đ ± SD, n = 2). Color coding is as in (A). TNKS2 WT reference data, from the same experiment, are identical to Figure 2C. See Figure S3B for eluate analyses by SDS-PAGE and Figures S5A and S5B for CD spectroscopy.

(C) EM of TNKS2 SAM domains. Color coding is as in (A). See Figure S3F for further mutants. Scale bars, 50 nm.

(D) ITC analysis for the indicated SAM domain pairs. Mutated surfaces are indicated by the star in the schematics. See Figure S5C for a second experiment.

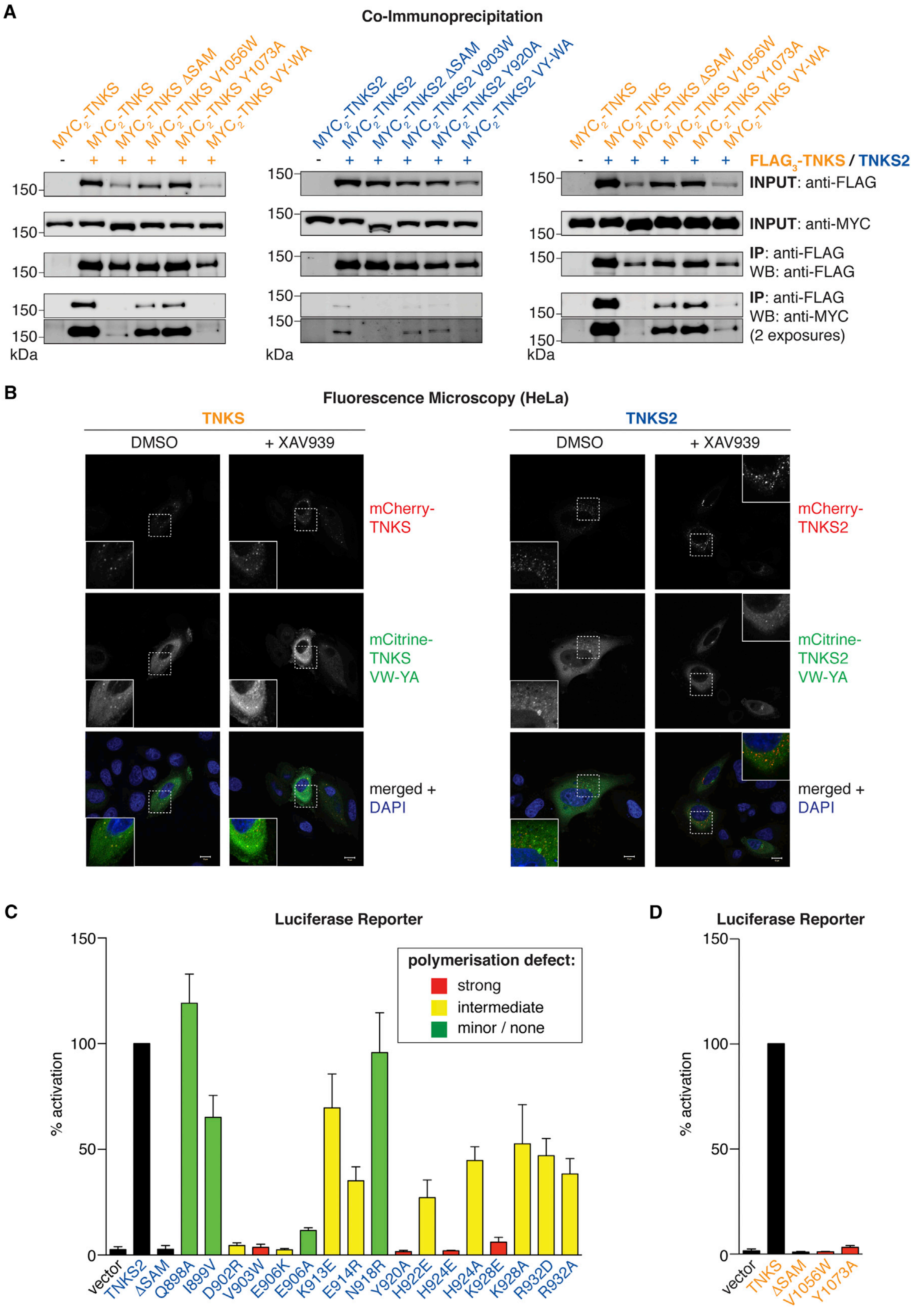

(legend on next page)

HEK293T cells (avoiding AXIN overexpression to maintain limiting levels), and we assessed its binding to MYC$_2$-TNKS2 (Figure 7D). AXIN1 robustly bound to TNKS2 and its catalytically inactive mutant G1032W^{T2}. However, recovery of TNKS2 ΔSAM^{T2}, V903W^{T2}, Y920A^{T2}, and the xx3xx mutant was strongly reduced (Figure 7D). Taken together, the microscopy and binding studies illustrate that SAM domain-mediated polymerization promotes Tankyrase interaction with AXIN in β-catenin degradasomes.

DISCUSSION

We propose a model in which multivalency, mediated by two Tankyrase-binding motifs in AXIN (Morrone et al., 2012) and four AXIN-binding ARCs in Tankyrase (Guettler et al., 2011), combined with polymerization of both proteins, gives rise to avidity for efficient Tankyrase recruitment to DCs (Figure 7E). Additionally, Tankyrase polymerization supports auto-PARylation and is expected to promote recruitment and activity of the E3 ubiquitin ligase RNF146, which also binds the ARCs (DaRosa et al., 2015). Our observation that Tankyrase-mediated scaffolding can drive Wnt-β-catenin signaling independently of catalytic PARP activity has important implications for the use of Tankyrase inhibitors to oppose oncogenic Wnt signaling.

The SAM-SAM contacts seen in our crystal structures are relevant to the full-length proteins. First, the SAM domains present their termini toward the filament periphery, compatible with protruding ARCs and PARP domains (Knight et al., 2011). Second, Tankyrase polymerization and its ability to activate β-catenin-dependent transcription correlate. Mutagenesis suggests that activation may require a TNKS- and TNKS2-specific polymerization threshold to be surpassed. Third, in co-IP, gel filtration, and light microscopy, full-length Tankyrases respond to mutation of the identified head-to-tail interfaces, in line with previous deletion studies (De Rycker and Price, 2004; Hatsugai et al., 2010). Tankyrase polymers display a punctate localization, as observed for other polymerizers, such as AXIN and Dishevelled in Wnt signaling (Fiedler et al., 2011), Polyhomeotic orthologs in transcriptional repression (Isono et al., 2013), and proteins of supramolecular organizing centers (SMOCs) in innate immune signaling (Kagan et al., 2014; Sherman et al., 2013). Puncta also were observed for endogenous Tankyrase in XAV939-treated colorectal cancer cells (de la Roche et al., 2014). Correlative light and EM showed that β-catenin DCs are of a filamentous sub-organization (Thorvaldsen et al., 2015). That these filaments do not grow to substantial length in cells likely reflects their dynamic nature (Bienz, 2014), a view compatible with micromolar SAM-SAM affinities and nanomolar Tankyrase concentrations in cells (Hein et al., 2015).

Compared to the TNKS2 SAM domain, that of TNKS polymerizes less efficiently. The higher molecular weight reported for chicken Tnks SAM polymers (De Rycker and Price, 2004) is based on elution volume, rather than static light scattering, and likely affected by the globular affinity tag and long flexible termini in the construct. Chicken MBP-Tnks SAM filaments are thus likely to be of similar length to the human TNKS SAM filaments analyzed here. R896^{T2}, responsible for differential polymerization of isolated TNKS and TNKS2 SAM domains, or a basic residue is conserved across TNKS2 orthologs and Tankyrases from species lacking TNKS2 (Figure 4D). However, its role remains unclear. First, our crystal structures do not reveal how R896^{T2} contributes to polymerization. Although all crystal structures of polymerizing SAM domains to date support the EH-ML interaction mode (Harada et al., 2008; Kim et al., 2001, 2002; Leettola et al., 2014; Nanyes et al., 2014; Stafford et al., 2011), crystallization may impose constraints onto some aspects of filament architecture and conceal the role of R896^{T2}. Second, TNKS and TNKS2 SAM domain affinities are similar by ITC, suggesting that the differences only become apparent in the context of WT filaments rather than pairs of mutant SAM domains. Third, the TNKS and TNKS2 SAM domains are mutually interchangeable for Wnt signaling, and the interconverting T1049R^{T1} and R896T^{T2} mutations do not appear to affect polymerization of the full-length proteins. Thus, differential polymerization may not occur in full-length Tankyrases or requires a yet unknown regulatory event. Given their heteropolymerization, the TNKS and TNKS2 pools may in fact not be separable.

Surprisingly, Tankyrase can induce β-catenin-dependent transcription independently of its catalytic PARP activity. The underlying mechanism relies on ARC- and SAM domain-dependent scaffolding but remains incompletely understood. Inactive Tankyrase may have a direct role in establishing β-catenin degradasomes (Martino-Echarri et al., 2016). TNKS or TNKS2 overexpression, either at the mRNA or protein level, has been described in numerous malignancies, including gastric cancer (Gao et al., 2011; Matsutani et al., 2001), breast cancer (Gelmini et al., 2004), bladder cancer (Gelmini et al., 2007), astrocytoma (Tang et al., 2012), glioblastoma (Shervington et al., 2007), pancreatic cancer (Zhao et al., 2009), lung cancer (Busch et al., 2013), and colon cancer (Gelmini et al., 2006; Shebzukhov et al., 2008). Polymerization and thus catalysis-independent Tankyrase functions may prevail when Tankyrase is overexpressed. Therefore, the effectiveness of catalytic Tankyrase inhibitors may be limited when Tankyrase levels are high (see

Figure 6. Tankyrase Requires Polymerization to Drive Wnt-β-Catenin Signaling

(A) Homo- and heterotypic interactions of TNKS and TNKS2 in HEK293T cells. FLAG$_3$-Tankyrases were immunoprecipitated, and co-precipitation of MYC$_2$-Tankyrases was assessed by SDS-PAGE and western blotting. TNKS2 expression is lower than TNKS, accounting for the weaker apparent TNKS2 self-association (Figure S1E). See Figures S6A and S6B for cell lysate fractionations and additional co-immunoprecipitations.

(B) Tankyrase polymerization controls localization. Serum-starved HeLa cells expressing the indicated mCherry- and mCitrine-tagged Tankyrases were vehicle or XAV939 treated. See Figures S6C and S6D for controls and additional experiments. Scale bar, 10 μm.

(C and D) Tankyrase polymerization controls Wnt-β-catenin signaling. Transcription reporter assays for selected TNKS2 and TNKS SAM domain mutants, as for Figure 1C. Reporter activity was normalized to WT TNKS2 or TNKS (100%). Color coding reflects polymerization defects of the corresponding SAM domains as assessed by sedimentation, SEC-MALS, and EM (see Figure 5; n = 3 duplicate experiments; error bars, SEM). See Figure S1 for expression levels and Figure S7 for further data.

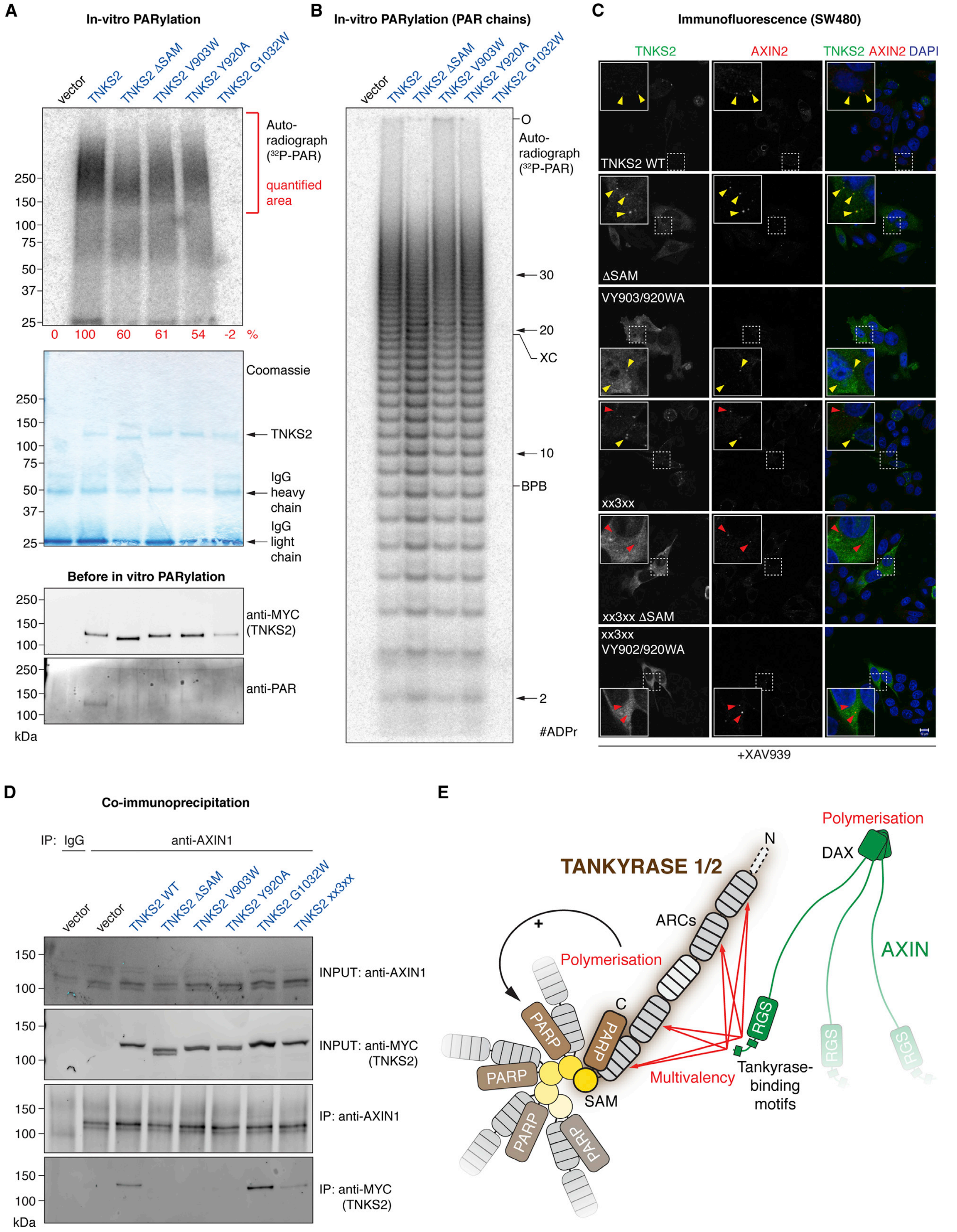

(legend on next page)

Figure 1D). Likewise, Tankyrase inhibitors stabilize Tankyrases through the blockage of PARdU (Huang et al., 2009), which may exacerbate polymerization. Blockage of scaffolding provides an additional promising avenue for pharmacologic inhibition of Tankyrase function.

Roles of polymeric Tankyrase likely extend beyond Wnt signaling, given the high prevalence of Tankyrase-binding proteins (Guettler et al., 2011). In analogy to SAM domain-containing transcriptional regulators (Isono et al., 2013), Tankyrase polymerization may facilitate protein regulation over an extensive physical range. Two such examples may be telomeres (Hsiao and Smith, 2008) and DNA repair sites (Nagy et al., 2016). Conversely, polymerization may suppress Tankyrase function in some cellular contexts. This study provides the tools to explore these questions.

EXPERIMENTAL PROCEDURES

The Supplemental Experimental Procedures are available in the Supplemental Information online.

Luciferase Reporters

HEK293T cells were transfected in technical triplicate with TOPFlash or FOPFlash reporter plasmids (Veeman et al., 2003), a reference Renilla luciferase reporter, and the indicated Tankyrase or AXIN constructs. One replicate was analyzed for protein expression. Cells were maintained in low serum (DMEM with 0.3% fetal bovine serum [FBS]) following transfection or treated with XAV939. Luciferase activities were measured 24 hr after transfection complex addition and Renilla luciferase activity used for normalization. Data were analyzed as detailed in the figure legends.

Protein Expression and Purification

SAM domains of human TNKS (1,018–1,093) and TNKS2 (867–940) were expressed in *E. coli* as His_6-MBP-Asn_{10} fusion proteins, and they were purified by Ni affinity purification, tag removal, anion exchange, and SEC. Proteins were dialyzed into buffer with 200 mM NaCl prior to experiments. Proteins shown in Figures 3C and 5A were affinity purified.

Crystallization, Structure Determination, and Analysis

Crystals of TNKS2 SAM DH902/924RE^{T2} and TNKS SAM D1055R^{T1} were grown and analyzed as detailed in the Supplemental Experimental Procedures. Crystal structures were determined by molecular replacement (Table 1). Interface residues were calculated using PISA (Proteins, Interfaces, Structures and Assemblies) (Krissinel and Henrick, 2007); contacts were analyzed and structural representations were generated using UCSF Chimera (Pettersen et al., 2004).

Ultracentrifugation Sedimentation

SAM domains were centrifuged at an average speed of 200,000 × *g* at 20°C for 1 hr. Total, supernatant, and pellet samples were analyzed by SDS-PAGE and Coomassie staining.

EM

SAM domains were applied to glow-discharged carbon-coated grids, negatively stained with 2% (w/v) uranyl acetate, and imaged on an FEI Tecnai 12 electron microscope.

SEC-MALS

Proteins were resolved by size exclusion in a buffer with 200 mM NaCl. In-line light scattering was measured using a DAWN Heleos-II (Wyatt) and refractive index using an Optilab rEX (Wyatt). Overall weight-average molecular weight (M_w) and dispersity (Đ) were calculated from two separate experiments analyzed in ASTRA (Wyatt).

ITC

All proteins were dialysed into binding buffer with 200 mM NaCl. TNKS2 SAM Y920A^{T2} or TNKS SAM Y1073A^{T1} (500 μM) was injected in 2-μl increments into TNKS2 SAM V903W^{T2} or TNKS SAM V1056W^{T1} (50 μM) or buffer, using an ITC200 MicroCalorimeter (MicroCal/GE Healthcare). Data were processed using Origin7 (MicroCal/GE Healthcare) using a one-site binding model.

In Vitro PARylation

MYC_2-TNKS2 and derivatives were expressed in HEK293T cells and immunoprecipitated. PARP activity assays were performed with 1 mM NAD^+ and 5 μCi ^{32}P-NAD^+ for 30 min at 30°C. PAR chains were detached and analyzed essentially as described previously (Alvarez-Gonzalez and Jacobson, 1987; Panzeter and Althaus, 1990). Immunoprecipitates and in vitro reactions were analyzed by western blotting and autoradiography, respectively.

Co-IPs

HEK293T cells were transfected with the indicated Tankyrase or control constructs. For Figure 7D, cells were serum starved to match luciferase assays. Immunoprecipitates with anti-AXIN1 (C76H11 clone, Cell Signaling Technologies) or control IgG (sc-2027, Santa Cruz Biotechnology) were captured on Protein A/G magnetic resin (Thermo Scientific/Pierce). For Figures 6A and S6B, IPs were performed with anti-FLAG M2 affinity gel (Sigma). Lysates and immunoprecipitates were analyzed by SDS-PAGE and western blotting.

Fluorescence Microscopy

HeLa or SW480 cells were transiently transfected with the indicated Tankyrase constructs. Cells in DMEM containing 0.3% FBS were treated either with DMSO vehicle or 2 μM XAV939 for 20 hr directly after transfection. Cells were fixed by the addition of 4% formaldehyde. Cells were immuno- and DAPI-stained as indicated.

ACCESSION NUMBERS

The accession numbers for the structure coordinates and experimental structure factors reported in this paper are PDB: 5JRT, 5JU5, and 5JTI.

SUPPLEMENTAL INFORMATION

Supplemental Information includes Supplemental Experimental Procedures, seven figures, and one table and can be found with this article online at http://dx.doi.org/10.1016/j.molcel.2016.06.019.

Figure 7. Tankyrase Polymerization Supports PARP Activity and Interaction with AXIN

(A) In vitro PARylation by immunoprecipitated MYC_2-TNKS2. Top: autoradiograph with quantitation is shown; middle: corresponding Coomassie-stained SDS-PAGE gel is shown, and bottom: western blot analysis of immunoprecipitates prior to in vitro PARylation is shown.

(B) PAR was released from samples analyzed in (A) and equal amounts of PAR, or all available sample for vector and TNKS2 G1032W, analyzed by PAGE and autoradiography. Origin (O), the xylene cyanol (XC) and bromophenol blue (BPB) markers and PAR chain length are indicated.

(C) SW480 cells expressing the indicated MYC_2-tagged TNKS2 constructs were XAV939 treated, fixed, and stained for MYC_2-TNKS2, endogenous AXIN2, and DNA. Yellow arrows denote degradasomes with AXIN2-TNKS2 colocalization; red arrows denote degradasomes containing AXIN2, but not TNKS2. Scale bar, 10 μm.

(D) Endogenous AXIN1 was immunoprecipitated from HEK293T cells expressing the indicated MYC_2-TNKS2 constructs. Samples were analyzed by SDS-PAGE and western blotting.

(E) A model for the role of polymers and multivalency in the Tankyrase-AXIN system. See the Discussion for details. Red arrows, interactions; black arrow, regulation.

AUTHOR CONTRIBUTIONS

C.M.T., L.M., P.P., M.R., and S.G. generated DNA constructs. L.M. and C.M.T. purified proteins. L.M. crystallized proteins, with N.C. collected diffraction data, and with N.C. and S.G. determined crystal structures. L.M. and C.M.T. performed ultracentrifugation assays. L.M. performed SEC-MALS, CD, and ITC. L.M. and F.B. performed EM and with E.M. analyzed the data. C.M.T. and S.G. performed luciferase reporters. M.R. performed PARP activity assays. L.M. and M.R. performed co-immunoprecipitations. M.R. and S.G. performed lysate fractionations and fluorescence microscopy. P.P. contributed to protein purification, ultracentrifugation, and EM. S.G. designed the study together with the other authors and supervised the research. S.G. wrote the manuscript with input from all authors.

ACKNOWLEDGMENTS

We thank members of the S.G. team and the Divisions of Structural and Cancer Biology for helpful comments. We are grateful to Tina Daviter (ISMB Biophysics Centre, Birkbeck College) for assistance with CD spectroscopy, Kevin Jackson for advice on SEC-MALS, Chris Richardson for IT support, Fredrik Wallberg for assistance with microscopy, and Jane Sandall for laboratory support. We thank the staff at beamline IO3 of the Diamond Light Source for their support during crystallographic data collection. We thank Alan Ashworth and Chris Lord for reagents and helpful discussions, Chris Bakal for HeLa cells, and Marc de la Roche for advice on AXIN immunofluorescence. We thank Frank Sicheri and the late Tony Pawson for support during the initial stages of this study. We are grateful to Mariann Bienz for valuable advice. We thank Scott Gradia, Robert Rottapel, Randall Moon, Frank Sicheri, Karen Colwill, Oliver Rocks, Robert Kingston, and Richard Treisman for plasmids. This work was supported by Cancer Research UK through a Career Establishment Award to S.G. (C47521/A16217) and The Institute of Cancer Research (ICR). L.M. is supported by an ICR studentship. E.M. is supported by Cancer Research UK (C12209/A16749).

Received: December 24, 2015
Revised: May 13, 2016
Accepted: June 13, 2016
Published: August 4, 2016

REFERENCES

Alvarez-Gonzalez, R., and Jacobson, M.K. (1987). Characterization of polymers of adenosine diphosphate ribose generated in vitro and in vivo. Biochemistry *26*, 3218–3224.

Bernardes de Jesus, B., and Blasco, M.A. (2013). Telomerase at the intersection of cancer and aging. Trends Genet. *29*, 513–520.

Bienz, M. (2014). Signalosome assembly by domains undergoing dynamic head-to-tail polymerization. Trends Biochem. Sci. *39*, 487–495.

Busch, A.M., Johnson, K.C., Stan, R.V., Sanglikar, A., Ahmed, Y., Dmitrovsky, E., and Freemantle, S.J. (2013). Evidence for tankyrases as antineoplastic targets in lung cancer. BMC Cancer *13*, 211.

Callow, M.G., Tran, H., Phu, L., Lau, T., Lee, J., Sandoval, W.N., Liu, P.S., Bheddah, S., Tao, J., Lill, J.R., et al. (2011). Ubiquitin ligase RNF146 regulates tankyrase and Axin to promote Wnt signaling. PLoS ONE *6*, e22595.

Canudas, S., Houghtaling, B.R., Kim, J.Y., Dynek, J.N., Chang, W.G., and Smith, S. (2007). Protein requirements for sister telomere association in human cells. EMBO J. *26*, 4867–4878.

Chiang, Y.J., Hsiao, S.J., Yver, D., Cushman, S.W., Tessarollo, L., Smith, S., and Hodes, R.J. (2008). Tankyrase 1 and tankyrase 2 are essential but redundant for mouse embryonic development. PLoS ONE *3*, e2639.

Clevers, H., Loh, K.M., and Nusse, R. (2014). Stem cell signaling. An integral program for tissue renewal and regeneration: Wnt signaling and stem cell control. Science *346*, 1248012.

DaRosa, P.A., Wang, Z., Jiang, X., Pruneda, J.N., Cong, F., Klevit, R.E., and Xu, W. (2015). Allosteric activation of the RNF146 ubiquitin ligase by a poly(ADP-ribosyl)ation signal. Nature *517*, 223–226.

de la Roche, M., Ibrahim, A.E., Mieszczanek, J., and Bienz, M. (2014). LEF1 and B9L shield β-catenin from inactivation by Axin, desensitizing colorectal cancer cells to tankyrase inhibitors. Cancer Res. *74*, 1495–1505.

De Rycker, M., and Price, C.M. (2004). Tankyrase polymerization is controlled by its sterile alpha motif and poly(ADP-ribose) polymerase domains. Mol. Cell. Biol. *24*, 9802–9812.

De Rycker, M., Venkatesan, R.N., Wei, C., and Price, C.M. (2003). Vertebrate tankyrase domain structure and sterile alpha motif (SAM)-mediated multimerization. Biochem. J. *372*, 87–96.

Dynek, J.N., and Smith, S. (2004). Resolution of sister telomere association is required for progression through mitosis. Science *304*, 97–100.

Fiedler, M., Mendoza-Topaz, C., Rutherford, T.J., Mieszczanek, J., and Bienz, M. (2011). Dishevelled interacts with the DIX domain polymerization interface of Axin to interfere with its function in down-regulating β-catenin. Proc. Natl. Acad. Sci. USA *108*, 1937–1942.

Gao, J., Zhang, J., Long, Y., Tian, Y., and Lu, X. (2011). Expression of tankyrase 1 in gastric cancer and its correlation with telomerase activity. Pathol. Oncol. Res. *17*, 685–690.

Gelmini, S., Poggesi, M., Distante, V., Bianchi, S., Simi, L., Luconi, M., Raggi, C.C., Cataliotti, L., Pazzagli, M., and Orlando, C. (2004). Tankyrase, a positive regulator of telomere elongation, is over expressed in human breast cancer. Cancer Lett. *216*, 81–87.

Gelmini, S., Poggesi, M., Pinzani, P., Mannurita, S.C., Cianchi, F., Valanzano, R., and Orlando, C. (2006). Distribution of Tankyrase-1 mRNA expression in colon cancer and its prospective correlation with progression stage. Oncol. Rep. *16*, 1261–1266.

Gelmini, S., Quattrone, S., Malentacchi, F., Villari, D., Travaglini, F., Giannarini, G., Della Melina, A., Pazzagli, M., Nicita, G., Selli, C., and Orlando, C. (2007). Tankyrase-1 mRNA expression in bladder cancer and paired urine sediment: preliminary experience. Clin. Chem. Lab. Med. *45*, 862–866.

Guettler, S., LaRose, J., Petsalaki, E., Gish, G., Scotter, A., Pawson, T., Rottapel, R., and Sicheri, F. (2011). Structural basis and sequence rules for substrate recognition by Tankyrase explain the basis for cherubism disease. Cell *147*, 1340–1354.

Haikarainen, T., Krauss, S., and Lehtio, L. (2014). Tankyrases: structure, function and therapeutic implications in cancer. Curr. Pharm. Des. *20*, 6472–6488.

Harada, B.T., Knight, M.J., Imai, S., Qiao, F., Ramachander, R., Sawaya, M.R., Gingery, M., Sakane, F., and Bowie, J.U. (2008). Regulation of enzyme localization by polymerization: polymer formation by the SAM domain of diacylglycerol kinase delta1. Structure *16*, 380–387.

Hatsugai, K., Ohishi, T., Sugimoto, Y., and Seimiya, H. (2010). Tankyrase-1 assembly to large protein complexes blocks its telomeric function. FEBS Lett. *584*, 3885–3890.

Hein, M.Y., Hubner, N.C., Poser, I., Cox, J., Nagaraj, N., Toyoda, Y., Gak, I.A., Weisswange, I., Mansfeld, J., Buchholz, F., et al. (2015). A human interactome in three quantitative dimensions organized by stoichiometries and abundances. Cell *163*, 712–723.

Hottiger, M.O., Hassa, P.O., Lüscher, B., Schüler, H., and Koch-Nolte, F. (2010). Toward a unified nomenclature for mammalian ADP-ribosyltransferases. Trends Biochem. Sci. *35*, 208–219.

Hsiao, S.J., and Smith, S. (2008). Tankyrase function at telomeres, spindle poles, and beyond. Biochimie *90*, 83–92.

Huang, S.-M.A., Mishina, Y.M., Liu, S., Cheung, A., Stegmeier, F., Michaud, G.A., Charlat, O., Wiellette, E., Zhang, Y., Wiessner, S., et al. (2009). Tankyrase inhibition stabilizes axin and antagonizes Wnt signalling. Nature *461*, 614–620.

Isono, K., Endo, T.A., Ku, M., Yamada, D., Suzuki, R., Sharif, J., Ishikura, T., Toyoda, T., Bernstein, B.E., and Koseki, H. (2013). SAM domain polymerization links subnuclear clustering of PRC1 to gene silencing. Dev. Cell *26*, 565–577.

Kagan, J.C., Magupalli, V.G., and Wu, H. (2014). SMOCs: supramolecular organizing centres that control innate immunity. Nat. Rev. Immunol. *14*, 821–826.

Karplus, P.A., and Diederichs, K. (2012). Linking crystallographic model and data quality. Science *336*, 1030–1033.

Kim, C.A., Phillips, M.L., Kim, W., Gingery, M., Tran, H.H., Robinson, M.A., Faham, S., and Bowie, J.U. (2001). Polymerization of the SAM domain of TEL in leukemogenesis and transcriptional repression. EMBO J. *20*, 4173–4182.

Kim, C.A., Gingery, M., Pilpa, R.M., and Bowie, J.U. (2002). The SAM domain of polyhomeotic forms a helical polymer. Nat. Struct. Biol. *9*, 453–457.

Knight, M.J., Leettola, C., Gingery, M., Li, H., and Bowie, J.U. (2011). A human sterile alpha motif domain polymerizome. Protein Sci. *20*, 1697–1706.

Krissinel, E., and Henrick, K. (2007). Inference of macromolecular assemblies from crystalline state. J. Mol. Biol. *372*, 774–797.

Lee, E., Salic, A., Krüger, R., Heinrich, R., and Kirschner, M.W. (2003). The roles of APC and Axin derived from experimental and theoretical analysis of the Wnt pathway. PLoS Biol. *1*, E10.

Leettola, C.N., Knight, M.J., Cascio, D., Hoffman, S., and Bowie, J.U. (2014). Characterization of the SAM domain of the PKD-related protein ANKS6 and its interaction with ANKS3. BMC Struct. Biol. *14*, 17.

Lehtiö, L., Chi, N.-W., and Krauss, S. (2013). Tankyrases as drug targets. FEBS J. *280*, 3576–3593.

Levaot, N., Voytyuk, O., Dimitriou, I., Sircoulomb, F., Chandrakumar, A., Deckert, M., Krzyzanowski, P.M., Scotter, A., Gu, S., Janmohamed, S., et al. (2011). Loss of Tankyrase-mediated destruction of 3BP2 is the underlying pathogenic mechanism of cherubism. Cell *147*, 1324–1339.

Li, V.S.W., Ng, S.S., Boersema, P.J., Low, T.Y., Karthaus, W.R., Gerlach, J.P., Mohammed, S., Heck, A.J.R., Maurice, M.M., Mahmoudi, T., and Clevers, H. (2012). Wnt signaling through inhibition of β-catenin degradation in an intact Axin1 complex. Cell *149*, 1245–1256.

Martino-Echarri, E., Brocardo, M.G., Mills, K.M., and Henderson, B.R. (2016). Tankyrase Inhibitors Stimulate the Ability of Tankyrases to Bind Axin and Drive Assembly of β-Catenin Degradation-Competent Axin Puncta. PLoS ONE *11*, e0150484.

Matsutani, N., Yokozaki, H., Tahara, E., Tahara, H., Kuniyasu, H., Haruma, K., Chayama, K., Yasui, W., and Tahara, E. (2001). Expression of telomeric repeat binding factor 1 and 2 and TRF1-interacting nuclear protein 2 in human gastric carcinomas. Int. J. Oncol. *19*, 507–512.

McCabe, N., Cerone, M.A., Ohishi, T., Seimiya, H., Lord, C.J., and Ashworth, A. (2009). Targeting Tankyrase 1 as a therapeutic strategy for BRCA-associated cancer. Oncogene *28*, 1465–1470.

Morrone, S., Cheng, Z., Moon, R.T., Cong, F., and Xu, W. (2012). Crystal structure of a Tankyrase-Axin complex and its implications for Axin turnover and Tankyrase substrate recruitment. Proc. Natl. Acad. Sci. USA *109*, 1500–1505.

Nagy, Z., Kalousi, A., Furst, A., Koch, M., Fischer, B., and Soutoglou, E. (2016). Tankyrases Promote Homologous Recombination and Check Point Activation in Response to DSBs. PLoS Genet. *12*, e1005791.

Nanyes, D.R., Junco, S.E., Taylor, A.B., Robinson, A.K., Patterson, N.L., Shivarajpur, A., Halloran, J., Hale, S.M., Kaur, Y., Hart, P.J., and Kim, C.A. (2014). Multiple polymer architectures of human polyhomeotic homolog 3 sterile alpha motif. Proteins *82*, 2823–2830.

Panzeter, P.L., and Althaus, F.R. (1990). High resolution size analysis of ADP-ribose polymers using modified DNA sequencing gels. Nucleic Acids Res. *18*, 2194.

Pettersen, E.F., Goddard, T.D., Huang, C.C., Couch, G.S., Greenblatt, D.M., Meng, E.C., and Ferrin, T.E. (2004). UCSF Chimera–a visualization system for exploratory research and analysis. J. Comput. Chem. *25*, 1605–1612.

Polakis, P. (2012). Wnt signaling in cancer. Cold Spring Harb. Perspect. Biol. *4*, a008052.

Qiao, F., and Bowie, J.U. (2005). The many faces of SAM. Sci. STKE *2005*, re7.

Riffell, J.L., Lord, C.J., and Ashworth, A. (2012). Tankyrase-targeted therapeutics: expanding opportunities in the PARP family. Nat. Rev. Drug Discov. *11*, 923–936.

Rippmann, J.F., Damm, K., and Schnapp, A. (2002). Functional characterization of the poly(ADP-ribose) polymerase activity of tankyrase 1, a potential regulator of telomere length. J. Mol. Biol. *323*, 217–224.

Seimiya, H., Muramatsu, Y., Smith, S., and Tsuruo, T. (2004). Functional subdomain in the ankyrin domain of tankyrase 1 required for poly(ADP-ribosyl)ation of TRF1 and telomere elongation. Mol. Cell. Biol. *24*, 1944–1955.

Shebzukhov, Y.V., Lavrik, I.N., Karbach, J., Khlgatian, S.V., Koroleva, E.P., Belousov, P.V., Kashkin, K.N., Knuth, A., Jager, E., Chi, N.-W., et al. (2008). Human tankyrases are aberrantly expressed in colon tumors and contain multiple epitopes that induce humoral and cellular immune responses in cancer patients. Cancer Immunol. Immunother. *57*, 871–881.

Sherman, E., Barr, V., and Samelson, L.E. (2013). Super-resolution characterization of TCR-dependent signaling clusters. Immunol. Rev. *251*, 21–35.

Shervington, A., Patel, R., Lu, C., Cruickshanks, N., Lea, R., Roberts, G., Dawson, T., and Shervington, L. (2007). Telomerase subunits expression variation between biopsy samples and cell lines derived from malignant glioma. Brain Res. *1134*, 45–52.

Smith, S., Giriat, I., Schmitt, A., and de Lange, T. (1998). Tankyrase, a poly(ADP-ribose) polymerase at human telomeres. Science *282*, 1484–1487.

Stafford, R.L., Hinde, E., Knight, M.J., Pennella, M.A., Ear, J., Digman, M.A., Gratton, E., and Bowie, J.U. (2011). Tandem SAM domain structure of human Caskin1: a presynaptic, self-assembling scaffold for CASK. Structure *19*, 1826–1836.

Stamos, J.L., and Weis, W.I. (2013). The β-catenin destruction complex. Cold Spring Harb. Perspect. Biol. *5*, a007898.

Tang, B., Wang, J., Fang, J., Jiang, B., Zhang, M., Wang, Y., and Yang, Z. (2012). Expression of TNKS1 is correlated with pathologic grade and Wnt/β-catenin pathway in human astrocytomas. J. Clin. Neurosci. *19*, 139–143.

Thorvaldsen, T.E., Pedersen, N.M., Wenzel, E.M., Schultz, S.W., Brech, A., Liestøl, K., Waaler, J., Krauss, S., and Stenmark, H. (2015). Structure, dynamics and functionality of tankyrase inhibitor-induced degradasomes. Mol. Cancer Res. *13*, 1487–1501.

Veeman, M.T., Slusarski, D.C., Kaykas, A., Louie, S.H., and Moon, R.T. (2003). Zebrafish prickle, a modulator of noncanonical Wnt/Fz signaling, regulates gastrulation movements. Curr. Biol. *13*, 680–685.

Wang, Z., Tacchelly-Benites, O., Yang, E., Thorne, C.A., Nojima, H., Lee, E., and Ahmed, Y. (2016). Wnt/Wingless Pathway Activation Is Promoted by a Critical Threshold of Axin Maintained by the Tumor Suppressor APC and the ADP-Ribose Polymerase Tankyrase. Genetics *203*, 269–281.

Winn, M.D., Ballard, C.C., Cowtan, K.D., Dodson, E.J., Emsley, P., Evans, P.R., Keegan, R.M., Krissinel, E.B., Leslie, A.G.W., McCoy, A., et al. (2011). Overview of the CCP4 suite and current developments. Acta Crystallogr. D Biol. Crystallogr. *67*, 235–242.

Wu, H. (2013). Higher-order assemblies in a new paradigm of signal transduction. Cell *153*, 287–292.

Yang, E., Tacchelly-Benites, O., Wang, Z., Randall, M.P., Tian, A., Benchabane, H., Freemantle, S., Pikielny, C., Tolwinski, N.S., Lee, E., and Ahmed, Y. (2016). Wnt pathway activation by ADP-ribosylation. Nat. Commun. *7*, 11430.

Yu, M., Schreek, S., Cerni, C., Schamberger, C., Lesniewicz, K., Poreba, E., Vervoorts, J., Walsemann, G., Grötzinger, J., Kremmer, E., et al. (2005). PARP-10, a novel Myc-interacting protein with poly(ADP-ribose) polymerase activity, inhibits transformation. Oncogene *24*, 1982–1993.

Zhang, Y., Liu, S., Mickanin, C., Feng, Y., Charlat, O., Michaud, G.A., Schirle, M., Shi, X., Hild, M., Bauer, A., et al. (2011). RNF146 is a poly(ADP-ribose)-directed

E3 ligase that regulates axin degradation and Wnt signalling. Nat. Cell Biol. *13*, 623–629.

Zhao, F., Vermeer, B., Lehmann, U., Kreipe, H., Manns, M.P., Korangy, F., and Greten, T.F. (2009). Identification of a novel murine pancreatic tumour antigen, which elicits antibody responses in patients with pancreatic carcinoma. Immunology *128*, 134–140.

Note Added in Proof

While this study was in press, DaRosa et al. reported a model for the TNKS SAM domain polymer, supported by biophysical evidence. The findings are in agreement with this study.

DaRosa, P.A., Ovchinnikov, S., Xu, W., and Klevit, R.E. (2016). Structural insights into SAM domain-mediated tankyrase oligomerization. Protein Sci. Published online June 21, 2016. http://dx.doi.org/10.1002/pro.2968.

Molecular Cell, Volume *63*

Supplemental Information

Tankyrase Requires SAM Domain-Dependent Polymerization to Support Wnt-β-Catenin Signaling

Laura Mariotti, Catherine M. Templeton, Michael Ranes, Patricia Paracuellos, Nora Cronin, Fabienne Beuron, Edward Morris, and Sebastian Guettler

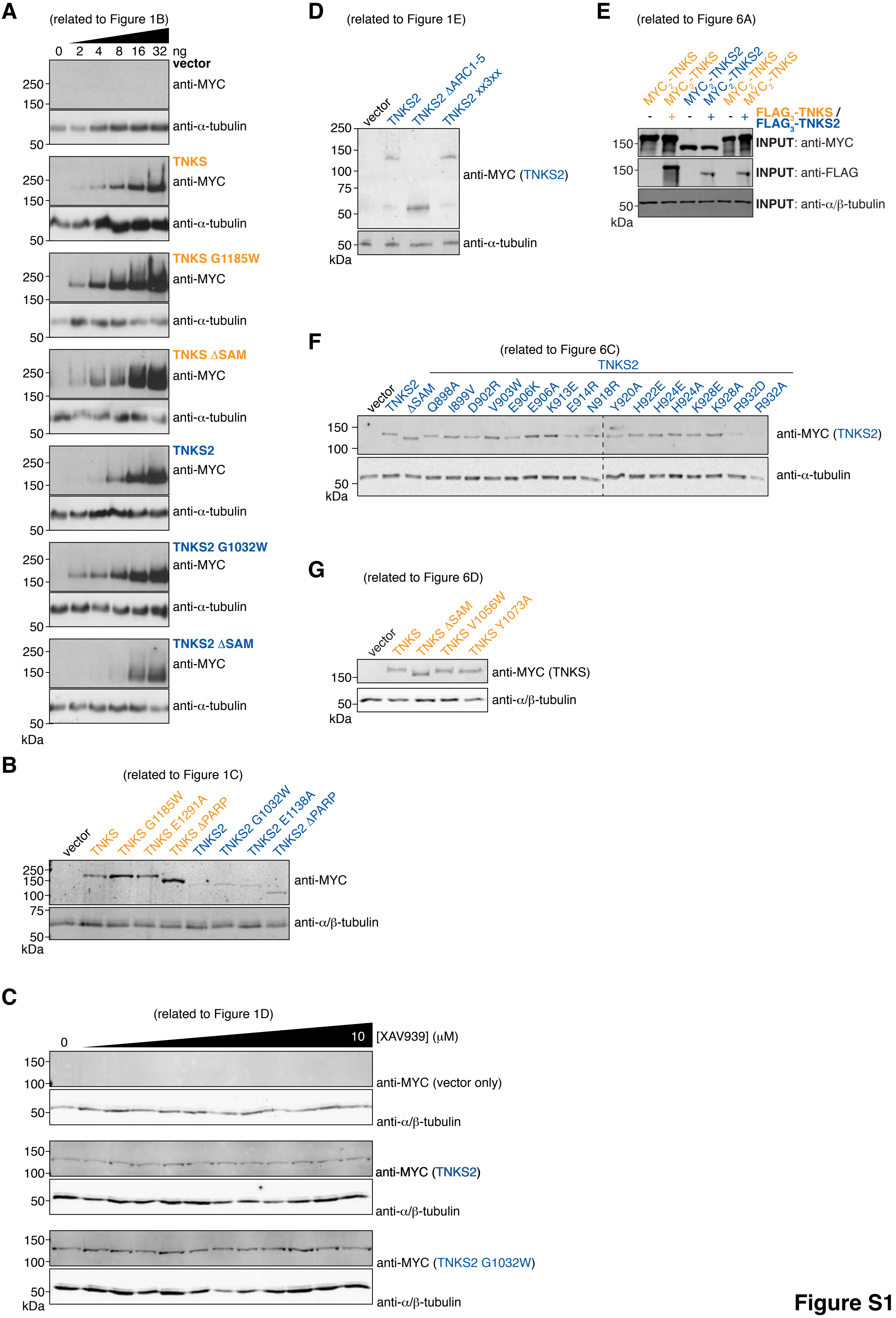

A
(related to Figure 1B)
0 2 4 8 16 32 ng
vector
TNKS
TNKS G1185W
TNKS ΔSAM
TNKS2
TNKS2 G1032W
TNKS2 ΔSAM
anti-MYC
anti-α-tubulin
250
150
50
kDa
B
(related to Figure 1C)
vector
TNKS
TNKS G1185W
TNKS E1291A
TNKS ΔPARP
TNKS2
TNKS2 G1032W
TNKS2 E1138A
TNKS2 ΔPARP
anti-MYC
anti-α/β-tubulin
250
150
100
75
50
kDa
C
(related to Figure 1D)
0
10
[XAV939] (μM)
anti-MYC (vector only)
anti-α/β-tubulin
anti-MYC (TNKS2)
anti-MYC (TNKS2 G1032W)
150
100
50
kDa
D
(related to Figure 1E)
vector
TNKS2
TNKS2 ΔARC1-5
TNKS2 xx3xx
anti-MYC (TNKS2)
anti-α-tubulin
250
150
100
75
50
kDa
E
(related to Figure 6A)
MYC2-TNKS
MYC2-TNKS2
MYC2-TNKS2
MYC2-TNKS2
MYC2-TNKS
MYC2-TNKS
- + - + - +
FLAG3-TNKS /
FLAG3-TNKS2
INPUT: anti-MYC
INPUT: anti-FLAG
INPUT: anti-α/β-tubulin
150
50
kDa
F
(related to Figure 6C)
TNKS2
vector
TNKS2
ΔSAM
Q898A
I899V
D902R
V903W
E906K
E906A
K913E
E914R
N918R
Y920A
H922E
H924E
H924A
K928E
K928A
R932D
R932A
anti-MYC (TNKS2)
anti-α-tubulin
150
100
50
kDa
G
(related to Figure 6D)
vector
TNKS
TNKS ΔSAM
TNKS V1056W
TNKS Y1073A
anti-MYC (TNKS)
anti-α/β-tubulin
150
50
kDa

Figure S1

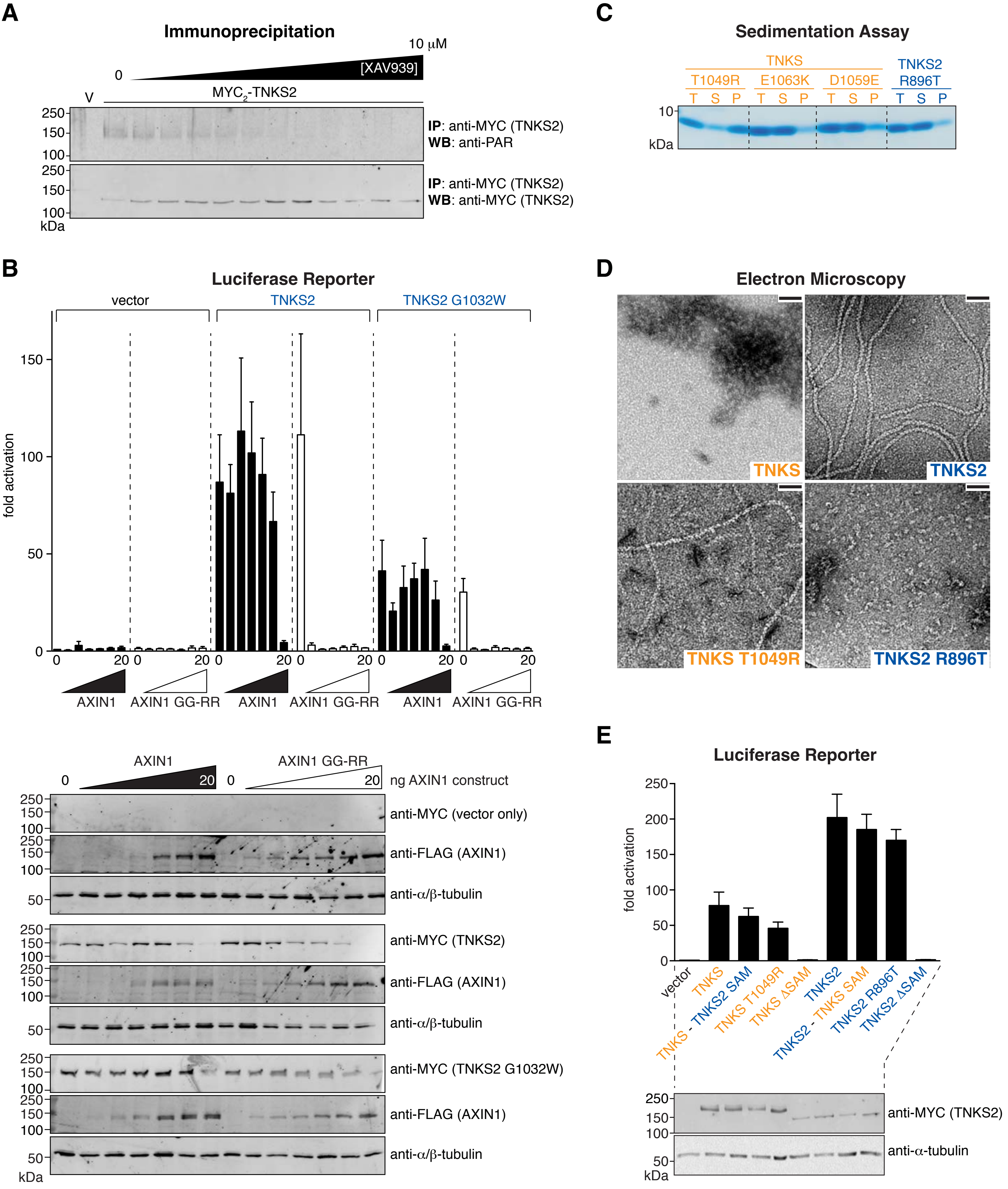
A
Immunoprecipitation
10 μM
0
[XAV939]
V
MYC2-TNKS2
250
150
100
IP: anti-MYC (TNKS2)
WB: anti-PAR
250
150
100
kDa
IP: anti-MYC (TNKS2)
WB: anti-MYC (TNKS2)
B
Luciferase Reporter
vector
TNKS2
TNKS2 G1032W
fold activation
150
100
50
0
0
20
AXIN1
AXIN1 GG-RR
AXIN1
0
20
ng AXIN1 construct
anti-MYC (vector only)
anti-FLAG (AXIN1)
anti-α/β-tubulin
anti-MYC (TNKS2)
anti-MYC (TNKS2 G1032W)
kDa
C
Sedimentation Assay
TNKS
T1049R
E1063K
D1059E
TNKS2
R896T
T S P
10
kDa
D
Electron Microscopy
TNKS
TNKS2
TNKS T1049R
TNKS2 R896T
E
Luciferase Reporter
250
200
150
100
50
0
fold activation
vector
TNKS
TNKS - TNKS2 SAM
TNKS T1049R
TNKS ΔSAM
TNKS2
TNKS2 - TNKS SAM
TNKS2 R896T
TNKS2 ΔSAM
anti-MYC (TNKS2)
anti-α-tubulin

Figure S2

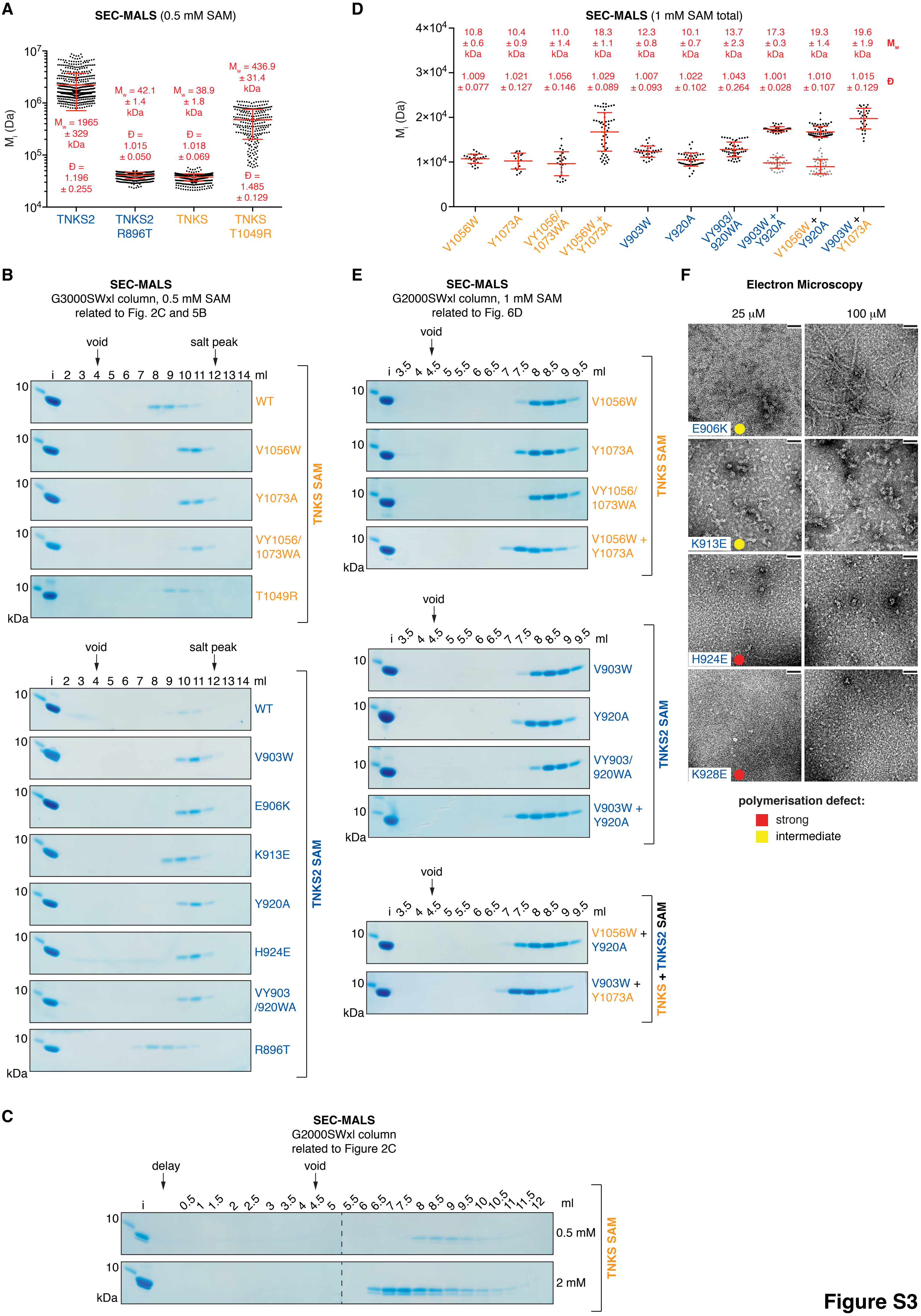
A
SEC-MALS (0.5 mM SAM)
M_i (Da)
M_w = 1965 ± 329 kDa
Đ = 1.196 ± 0.255
M_w = 42.1 ± 1.4 kDa
Đ = 1.015 ± 0.050
M_w = 38.9 ± 1.8 kDa
Đ = 1.018 ± 0.069
M_w = 436.9 ± 31.4 kDa
Đ = 1.485 ± 0.129
TNKS2
TNKS2 R896T
TNKS
TNKS T1049R
D
SEC-MALS (1 mM SAM total)
M_i (Da)
M_w
Đ
10.8 ± 0.6 kDa
10.4 ± 0.9 kDa
11.0 ± 1.4 kDa
18.3 ± 1.1 kDa
12.3 ± 0.8 kDa
10.1 ± 0.7 kDa
13.7 ± 2.3 kDa
17.3 ± 0.3 kDa
19.3 ± 1.4 kDa
19.6 ± 1.9 kDa
1.009 ± 0.077
1.021 ± 0.127
1.056 ± 0.146
1.029 ± 0.089
1.007 ± 0.093
1.022 ± 0.102
1.043 ± 0.264
1.001 ± 0.028
1.010 ± 0.107
1.015 ± 0.129
V1056W
Y1073A
VY1056/1073WA
V1056W + Y1073A
V903W
Y920A
VY903/920WA
V903W + Y920A
V1056W + Y920A
V903W + Y1073A
B
SEC-MALS
G3000SWxl column, 0.5 mM SAM
related to Fig. 2C and 5B
void
salt peak
i 2 3 4 5 6 7 8 9 10 11 12 13 14 ml
kDa
WT
V1056W
Y1073A
VY1056/1073WA
T1049R
TNKS SAM
WT
V903W
E906K
K913E
Y920A
H924E
VY903/920WA
R896T
TNKS2 SAM
E
SEC-MALS
G2000SWxl column, 1 mM SAM
related to Fig. 6D
void
i 3.5 4 4.5 5 5.5 6 6.5 7 7.5 8 8.5 9 9.5 ml
V1056W
Y1073A
VY1056/1073WA
V1056W + Y1073A
TNKS SAM
V903W
Y920A
VY903/920WA
V903W + Y920A
TNKS2 SAM
V1056W + Y920A
V903W + Y1073A
TNKS + TNKS2 SAM
F
Electron Microscopy
25 µM
100 µM
E906K
K913E
H924E
K928E
polymerisation defect:
strong
intermediate
C
SEC-MALS
G2000SWxl column
related to Figure 2C
delay
void
i 0.5 1 1.5 2 2.5 3 3.5 4 4.5 5 5.5 6 6.5 7 7.5 8 8.5 9 9.5 10 10.5 11 11.5 12 ml
0.5 mM
2 mM
TNKS SAM

Figure S3

A

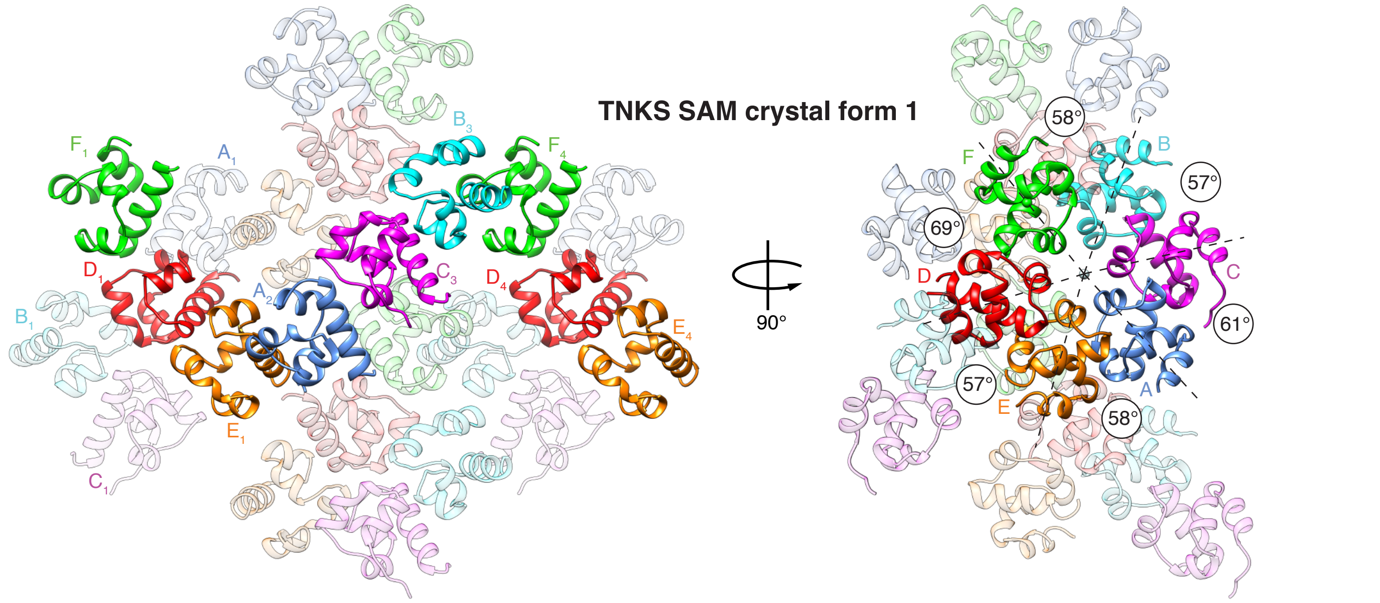

B

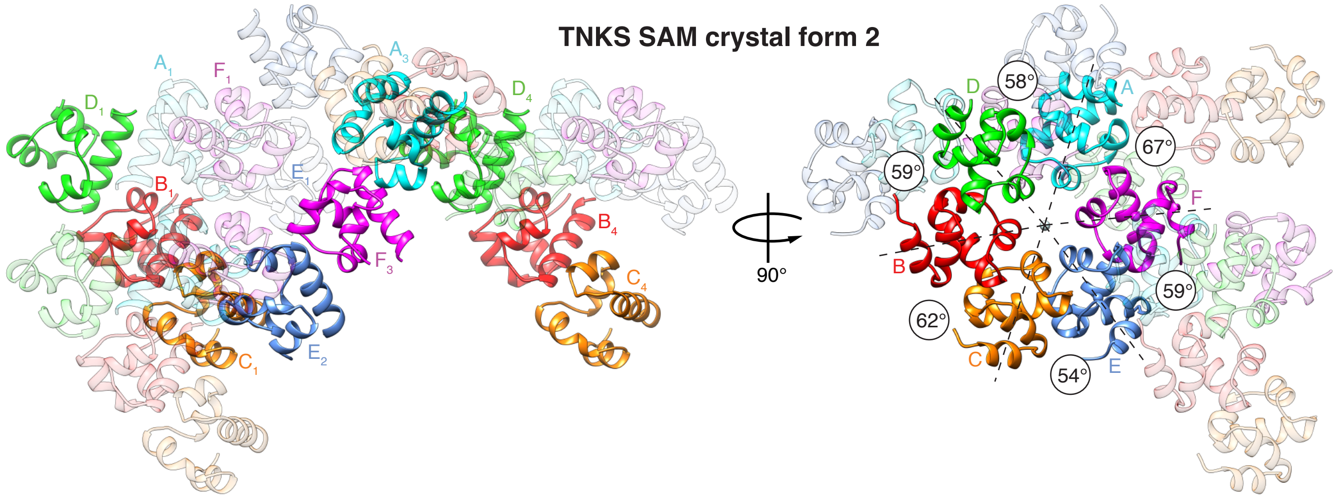

C

Contact Analysis

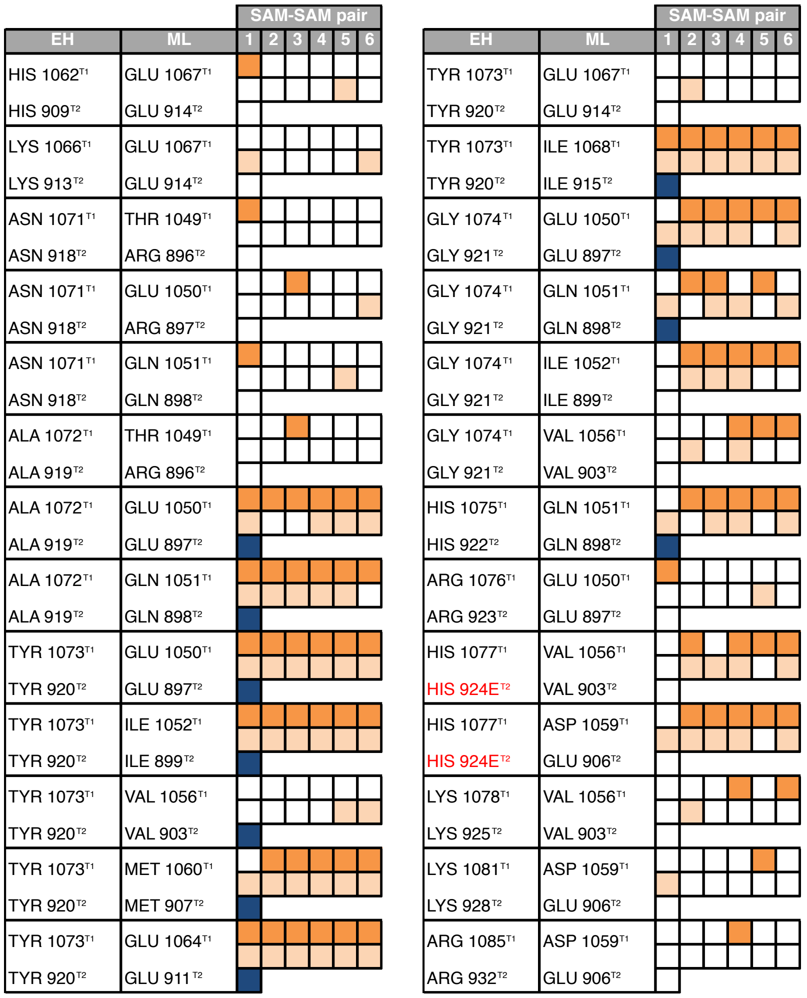

SAM-SAM pair	TNKS SAM crystal form 1	TNKS SAM crystal form 2	TNKS2 SAM
1	F_1-D_1	D_1-B_1	A-B
2	D_1-E_1	B_1-C_1	
3	E_1-A_2	C_1-E_2	
4	A_2-C_3	E_2-F_3	
5	C_3-B_3	F_3-A_3	
6	B_3-F_4	A_3-D_4	

Figure S4

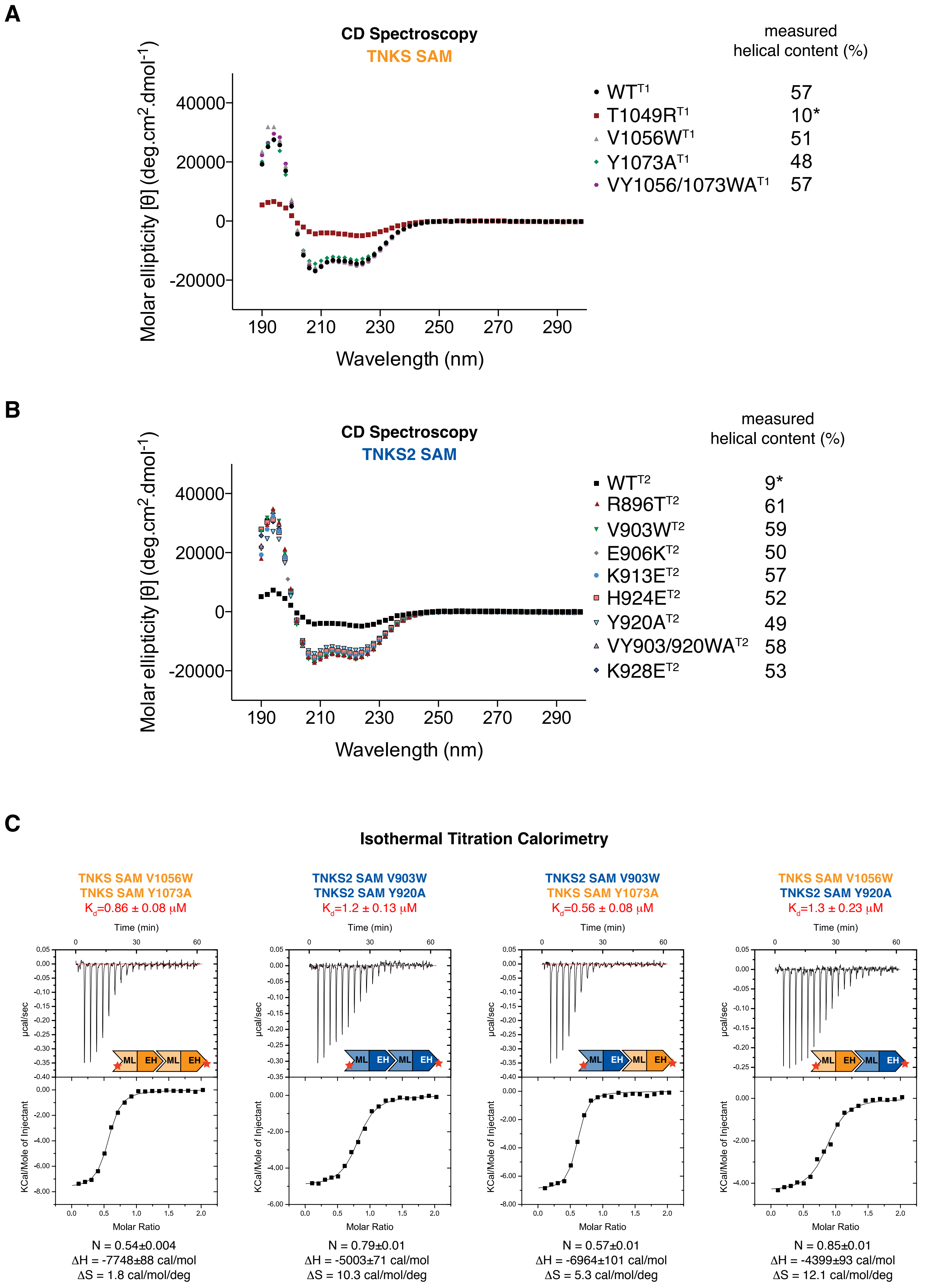
A
CD Spectroscopy
TNKS SAM
measured helical content (%)
WT T1 57
T1049R T1 10*
V1056W T1 51
Y1073A T1 48
VY1056/1073WA T1 57
Molar ellipticity [θ] (deg.cm2.dmol-1)
Wavelength (nm)
B
CD Spectroscopy
TNKS2 SAM
measured helical content (%)
WT T2 9*
R896T T2 61
V903W T2 59
E906K T2 50
K913E T2 57
H924E T2 52
Y920A T2 49
VY903/920WA T2 58
K928E T2 53
Molar ellipticity [θ] (deg.cm2.dmol-1)
Wavelength (nm)
C
Isothermal Titration Calorimetry
TNKS SAM V1056W
TNKS SAM Y1073A
Kd=0.86 ± 0.08 µM
TNKS2 SAM V903W
TNKS2 SAM Y920A
Kd=1.2 ± 0.13 µM
TNKS2 SAM V903W
TNKS SAM Y1073A
Kd=0.56 ± 0.08 µM
TNKS SAM V1056W
TNKS2 SAM Y920A
Kd=1.3 ± 0.23 µM
Time (min)
µcal/sec
KCal/Mole of Injectant
Molar Ratio
ML
EH
N = 0.54±0.004
ΔH = -7748±88 cal/mol
ΔS = 1.8 cal/mol/deg
N = 0.79±0.01
ΔH = -5003±71 cal/mol
ΔS = 10.3 cal/mol/deg
N = 0.57±0.01
ΔH = -6964±101 cal/mol
ΔS = 5.3 cal/mol/deg
N = 0.85±0.01
ΔH = -4399±93 cal/mol
ΔS = 12.1 cal/mol/deg

Figure S5

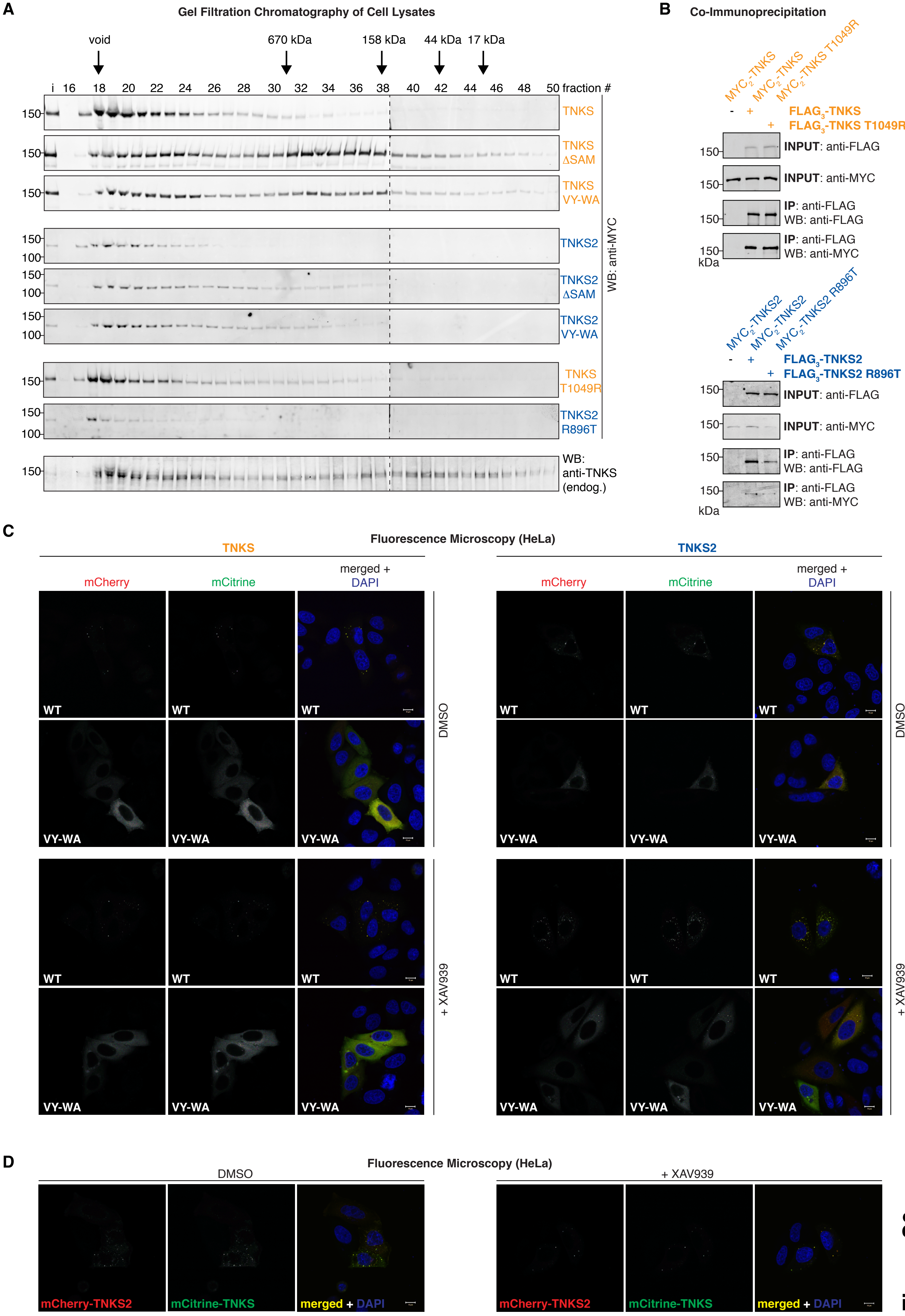

A
Gel Filtration Chromatography of Cell Lysates
void
670 kDa
158 kDa
44 kDa
17 kDa
i 16 18 20 22 24 26 28 30 32 34 36 38 40 42 44 46 48 50 fraction #
150
TNKS
TNKS ΔSAM
TNKS VY-WA
100
TNKS2
TNKS2 ΔSAM
TNKS2 VY-WA
WB: anti-MYC
TNKS T1049R
TNKS2 R896T
WB: anti-TNKS (endog.)
B
Co-Immunoprecipitation
MYC2-TNKS
FLAG3-TNKS
FLAG3-TNKS T1049R
INPUT: anti-FLAG
INPUT: anti-MYC
IP: anti-FLAG WB: anti-FLAG
IP: anti-FLAG WB: anti-MYC
kDa
MYC2-TNKS2
FLAG3-TNKS2
FLAG3-TNKS2 R896T
C
Fluorescence Microscopy (HeLa)
TNKS
TNKS2
mCherry
mCitrine
merged + DAPI
WT
VY-WA
DMSO
+ XAV939
D
Fluorescence Microscopy (HeLa)
DMSO
+ XAV939
mCherry-TNKS2
mCitrine-TNKS
merged + DAPI

Figure S6

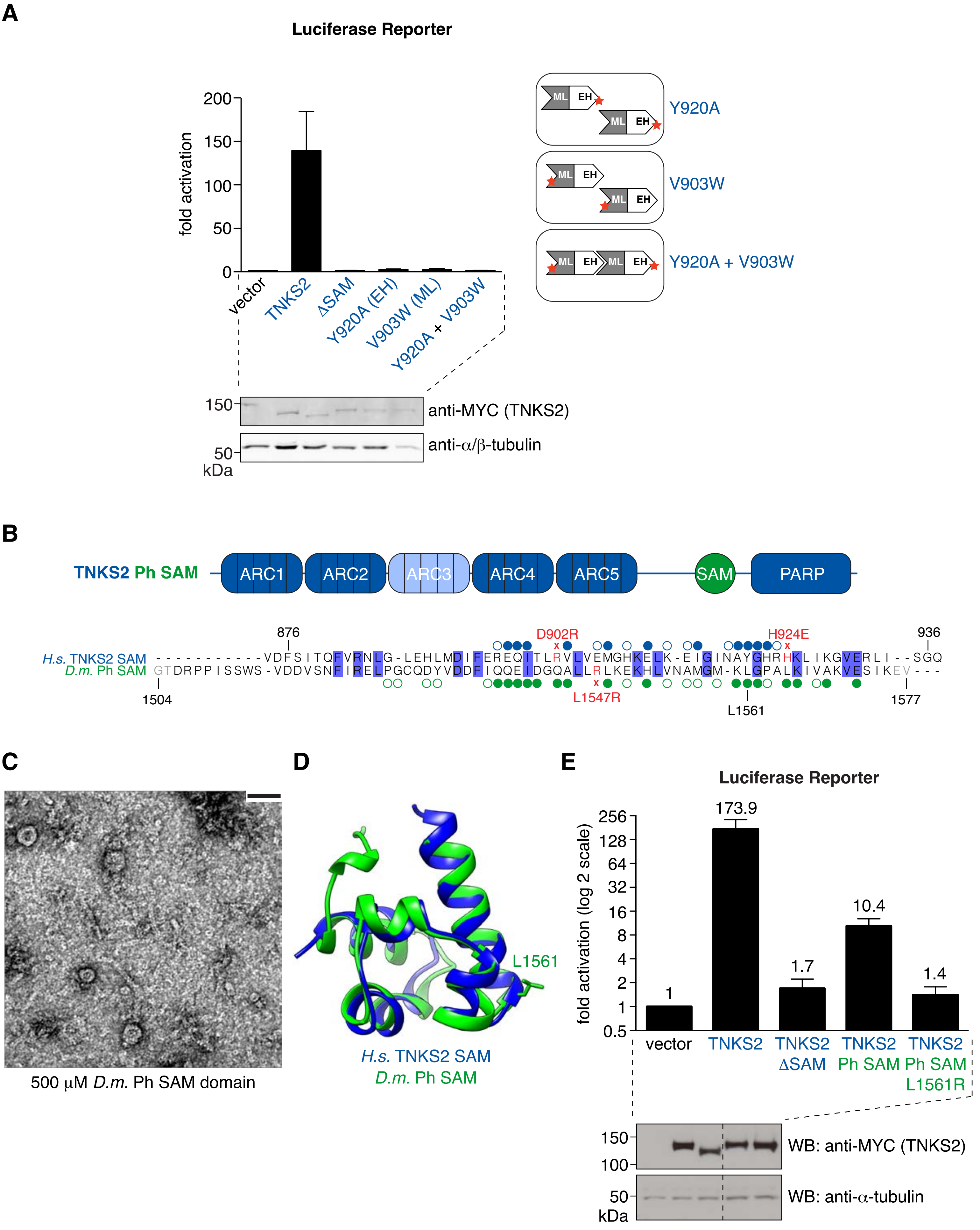
A
Luciferase Reporter
fold activation
200
150
100
50
0
vector
TNKS2
ΔSAM
Y920A (EH)
V903W (ML)
Y920A + V903W
ML
EH
Y920A
V903W
Y920A + V903W
150
50
kDa
anti-MYC (TNKS2)
anti-α/β-tubulin
B
TNKS2 Ph SAM
ARC1
ARC2
ARC3
ARC4
ARC5
SAM
PARP
876
936
D902R
H924E
H.s. TNKS2 SAM
D.m. Ph SAM
1504
L1547R
L1561
1577
C
500 μM D.m. Ph SAM domain
D
L1561
H.s. TNKS2 SAM
D.m. Ph SAM
E
Luciferase Reporter
fold activation (log 2 scale)
256
128
64
32
16
8
4
2
1
0.5
173.9
10.4
1.7
1.4
1
vector
TNKS2
TNKS2 ΔSAM
TNKS2 Ph SAM
TNKS2 Ph SAM L1561R
150
100
50
kDa
WB: anti-MYC (TNKS2)
WB: anti-α-tubulin

Figure S7

Supplemental Figure Legends

Figure S1: Protein expression levels in luciferase reporter assays and immunoprecipitation, related to Figures 1 and 6

(A) to **(D)**, **(F)**, **(G)** HEK293T cells were transiently transfected in technical triplicate for luciferase reporter assays. Two replicates were assessed for luciferase activity (see main figures). A third replicate was analysed by SDS-PAGE and Western blotting as indicated to assess protein expression levels. The dashed line in (F) separates lanes from two different gels/membranes. **(E)** The same INPUT samples shown in the first two lanes of each of the three panels of Figure 6A were analysed by SDS-PAGE and Western blotting on the same gel and membrane for direct comparison, as indicated. MYC_2-TNKS is consistently more highly expressed than MYC_2-TNKS2.

Figure S2: Tankyrase modulation by enzymatic inhibition, AXIN1 and the SAM domain, related to Figures 1 and 2

(A) HEK293T cells were transfected with MYC_2-TNKS2 and treated with the same concentrations of XAV939 as for Figure 1D for 20 h. MYC_2-TNKS2 was immunoprecipitated, and immunoprecipitates were analysed for PAR by Western blotting as indicated. **(B)** TOPFlash transcription reporter assay. MYC_2-TNKS2, TNKS2 G1032W^{T2} or empty vector (16 ng) were co-expressed with increasing amounts of the indicated $FLAG_3$-AXIN1 constructs (0.6 to 20 ng in a two-fold dilution series), either in wild-type form or as Tankyrase-binding deficient mutant (GG27/74RR, GG-RR). Data are expressed relative to the mean reporter activity in the "vector" control without AXIN1. n=3 independent experiments done in technical duplicate; error bars, SEM. Western blots to assess protein expression levels in the assay are shown below. Note that at the highest dose of AXIN1 construct, TNKS2 expression is reduced, accounting for the drop in reporter activation at this dose. **(C)** Ultracentrifugation sedimentation assay as in Figure 2A. Data are from the same experiment as those shown in Figure 2A. **(D)** Electron microscopy analysis of negatively

stained Tankyrase SAM domains at 0.025 mM. See Figure S3A for SEC-MALS analysis. **(E)** TOPFlash transcription reporter assay as in Figure 1C, using 16 ng of MYC_2-TNKS2 constructs. n=3 to 6 independent experiments done in technical duplicate; error bars, SEM. Tankyrase expression levels in the assay are shown below.

Figure S3: SEC-MALS and EM of Tankyrase SAM domains, related to Figures 2 and 5

(A) and **(D)** Molecular weight scatter plots from two separate SEC-MALS analyses for the indicated SAM domains at 0.5 mM (A) and 1 mM in total (0.5 + 0.5 mM for paired mutant domains) (D). Weight-average molecular weights (M_w) and dispersity (Đ) ±SD are indicated. Plotted data points with mean and SD refer to single molecular weight data points (M_i) corresponding to measurement slices. SEC-MALS for V903W^{T2} + Y920A^{T2} and V1056W^{T1} + Y920A^{T2} TNKS2, shown in (D), also revealed monomeric sub-populations (grey data points). M_w and Đ shown for these samples refer to the dimeric sub-population only. TNKS2 WT reference data are the same as shown in Figures 2C and 5B (acquired as part of the same sample set). Likewise, TNKS WT reference data are the same as shown in Figure 5B; TNKS SAM T1049R^{T1} data were acquired as part of the same sample set. See below ((B) and (E)) for an analysis of the corresponding elution fractions. See Figures S5A and B for quality control of purified mutant proteins by CD spectroscopy. **(B)**, **(C)**, **(E)** Samples from SEC-MALS elution fractions (10 μl) were analysed by SDS-PAGE and Coomassie Brilliant Blue staining. Void volume (V_0) and salt peak volume are indicated where corresponding fractions were analysed. i, input (1 μl). Input protein concentration and column are indicated. The dashed line in (C) separates lanes from two different gels. **(F)** Electron microscopy analysis of the indicated TNKS2 SAM domains at 25 μM and 100 μM. Scale bars, 50 nm. Colour coding indicates the degree of abrogated sedimentation, as in Figure 5.

Figure S4: TNKS SAM domain packing and comparative analysis of SAM-SAM contacts in the TNKS and TNKS2 SAM domain crystal structures, related to Figure 4

Non-crystallographic and crystallographic symmetry in two TNKS D1055R^{T1} SAM domain crystal forms gives rise to filaments in the crystals. **(A)** Crystal form 1. **(B)** Crystal form 2. In both cases, the asymmetric unit consists of six SAM domains, but the two asymmetric units are distinct from each other. Left, four adjacent asymmetric units are shown in ribbon representation with one filament in side view highlighted by higher opacity. The six chains within the left-most asymmetric unit are named A_1 to F_1. Subscript numbers of chain identifiers denote the corresponding asymmetric units (1-4). A repeating unit in the filament consists of six protomers. Right, the same assembly rotated by 90° along the y axis reveals the approximate six-fold symmetry. Centroids for each SAM domain in the repeating unit, depicted as spheres, were calculated for TNKS SAM residues 1030-1087. Approximate angles between the centroids of each SAM domain in the axial projection were measured, using the overall centroid of all six centroids as vertex. **(C)** Contact analysis for all SAM-SAM domain pairs observed in all three crystal structures, analysed in UCSF Chimera. SAM-SAM domain pairs are numbered as indicated in the table on the right. A coloured field (TNKS crystal form 1, orange; TNKS crystal form 2, pale orange; TNKS2, blue) represents the occurrence of the respective contact.

Figure S5: CD spectroscopy analysis of purified TNKS and TNKS2 SAM domains and a second ITC experiment, related to Figure 5

(A) and **(B)** Purified proteins at 0.2 mg/ml were analysed by CD spectroscopy. The calculated helical contents of the TNKS and TNKS2 SAM domains from the crystal structures is 61%. Measured helical contents are comparable, with the exception of strongly polymerising SAM domains (TNKS2 SAM and TNKS SAM T1049R^{T1}, denoted by asterisks), where helical contents appear underestimated. Reduced molar ellipticities were previously observed for a polymerising C-terminal truncation of serum amyloid A (SSA), and absorption flattening due to a changed protein

environment in the polymer was proposed to account for this effect (Patke et al., 2012). The observation that SAM domains with mutations in opposite interfaces are still able to form dimers (see Figures S3D and E) provides additional documentation for their correct folding. **(C)** Replicate isothermal titration calorimetry (ITC) experiment as in Figure 5D.

Figure S6: Assessment of TNKS and TNKS2 polymerisation by biochemical assays and microscopy, related to Figure 6

(A) Full-length TNKS and TNKS2 polymerise in a SAM-domain dependent manner. Lysates from cells expressing the indicated MYC_2-Tankyrase constructs were fractionated by size exclusion chromatography. Fractions were analysed by Western blotting as indicated. Dashed lines separate lanes from two different gels/membranes. **(B)** The $T1049R^{T1}$ and $R896^{T2}$ mutations do not appear to affect Tankyrase self-interaction in the context of the full-length protein, at least under the experimental conditions. The indicated $FLAG_3$- and MYC_2-tagged TNKS and TNKS2 constructs were co-expressed in HEK293T cells and $FLAG_3$-Tankyrases immunoprecipitated, as for Figure 6A. Lysates and immunoprecipitates were analysed by SDS-PAGE and Western blotting as indicated. The observations are consistent with the size exclusion experiment shown in (A). **(C)** Tankyrase polymerisation controls localisation. Serum-starved HeLa cells expressing the indicated mCherry- and mCitrine-tagged Tankyrase constructs were either vehicle- or XAV939-treated as indicated, fixed and imaged by fluorescence microscopy. Microscopy data were obtained together with those shown in Figure 6B. Scale bar, 10 μm. **(D)** TNKS and TNKS2 colocalise. Microscopy was performed as for (C). Scale bar, 10 μm.

Figure S7: Polymerisation requirement of TNKS2 to drive Wnt-β-catenin signalling, related to Figure 6

(A) Co-expression of TNKS2 $V903W^{T2}$ and $Y920A^{T2}$, which would be able to form dimers (see Figures S3D and E), does not rescue the lost ability of the individual mutants to induce Wnt

signalling. TOPFlash transcription reporter assays for the indicated pairs of TNKS2 SAM domain mutant derivatives, performed as for Figure 1C. The total amount of MYC_2-TNKS2 construct per transfection was 16 ng (8 ng + 8 ng for paired TNKS2 mutants). n=3 independent experiments done in technical duplicate; error bars, SEM. Western blots to assess TNKS2 expression levels in the assay are shown below. The schematic on the right illustrates the formation of heterodimers between ML and EH mutant derivatives, which are monomeric on their own, as shown by SEC-MALS (see Figure S3D and E). Although unlikely, we cannot rule out that SAM-SAM interactions within the dimer are weakened by the distal mutations. **(B)-(E)** Polymerisation of an orthologous SAM domain partially compensates for loss of the SAM domain in TNKS2. (**B)** Top, schematic representation of a chimeric TNKS2 construct with the SAM domain of *D. melanogaster* Polyhomeotic (Ph). Bottom, structure-based alignment of the SAM domains of *H. sapiens* TNKS2 (DH902/924RE^{T2}) and *D. melanogaster* Ph (L1547R). Amino acids shown in grey are not resolved in the crystal structures. Mutations introduced for crystallization are indicated in red. Interface residues, as identified by PISA and defined through limited solvent accessibility, and explicit contact residues, as analysed in UCSF Chimera, are indicated by open and closed circles, respectively, as in Figure 4D. For the TNKS2 Ph SAM chimera, TNKS2 residues 876-936 were replaced by Ph residues 1504-1577, as indicated. **(C)** Electron micrograph of negatively stained *D. melanogaster* Ph SAM domain polymers. Scale bar, 50 nm. **(D)** Structural representations of the superimposed TNKS2 and *D. melanogaster* Ph SAM domains (PDB accession code: 1KW4). A L1561R mutation results in loss of Ph SAM domain polymerisation (Kim et al., 2002). **(E)** TOPFlash transcription reporter assay as in Figure 1C. n=6 independent experiments done in technical duplicate; error bars, SEM. Western blots to assess Tankyrase expression levels in the assay are shown below. The dashed line in the Western blot indicates the position where an irrelevant lane has been spliced out. The Ph SAM domain conferred a weak activation of the transcription reporter by TNKS2, illustrating incomplete rescue, but this was abolished by a validated, structure-based polymer-breaking mutation in the heterologous domain (Kim et al.,

2002). Note the log 2 scale.

Supplemental Tables

Table S1. Plasmids used in this study, related to Experimental Procedures
Point mutations and deletions were generated from these plasmids by site-directed mutagenesis (see section "Plasmids" in Extended Experimental Procedures).

plasmid name	species	accession no.	sites	references/information
**bacterial expression constructs**				
pET-His_6-MBP-Asn_{10}-TEV (1C)-TNKS(1018-1093)	*H. sapiens*	NM_003747.2	LIC v1	1
pET-His_6-MBP-Asn_{10}-TEV (1C)-TNKS2(867-940)	*H. sapiens*	NM_025235.3	LIC v1	1
pET-His_6-MBP-Asn_{10}-TEV (1C)-Ph(1502-1587)	*D. melanogaster*	NM_057523.5	LIC v1	1, 2
**mammalian expression constructs / reporter plasmids / vectors**				
pLP-dMYC SD-TNKS	*H. sapiens*	NM_003747.2	*AscI-PacI & loxP*	3, 4
pLP-dMYC SD-TNKS2	*H. sapiens*	NM_025235.3	*AscI-PacI & loxP*	4, 5
pLP-tripleFLAG SD-TNKS	*H. sapiens*	NM_003747.2	*AscI-PacI & loxP*	4
pLP-tripleFLAG SD-TNKS2	*H. sapiens*	NM_025235.3	*AscI-PacI & loxP*	4
pLP-mCitrine C1 SD-TNKS	*H. sapiens*	NM_003747.2	*AscI-PacI & loxP*	4, 6
pLP-mCitrine C1 SD-TNKS2	*H. sapiens*	NM_025235.3	*AscI-PacI & loxP*	4, 6
pLP-mCherry C1 SD-TNKS	*H. sapiens*	NM_003747.2	*AscI-PacI & loxP*	4, 6
pLP-mCherry C1 SD-TNKS2	*H. sapiens*	NM_025235.3	*AscI-PacI & loxP*	4, 6
pLP-tripleFLAG SD-AXIN1	*H. sapiens*	NM_003502.3	*AscI-PacI & loxP*	4, 7
M50 Super 8x TOPFlash	-	-	-	8
M51 Super 8x FOPFlash (TOPFlash mutant)	-	-	-	8
ptkRL	-	-	-	9
pDNR-MCS SA	-	-	-	4

[1] The empty vector was a gift from Dr. Scott Gradia (UC Berkeley) via Addgene (Addgene plasmid # 29654)
[2] The Ph cDNA was a gift from Dr. Robert Kingston (Harvard Medical School) via Addgene (Addgene plasmid # 1925) (Francis et al., 2001)
[3] The pLP-dMyc SD-TNKS plasmid was a kind gift from Dr. Robert Rottapel (OCI, Toronto).
[4] (Colwill et al., 2006)
[5] (Guettler et al., 2011)
[6] The pLP-Citrine C1 SD and pLP-mCherry C1 SD plasmids were a kind gift from Dr. Oliver Rocks (MDC, Berlin).
[7] The AXIN1 cDNA (OriGene) was a kind gift from Dr. Alan Ashworth and Dr. Chris Lord (ICR, London).
[8] M50 Super 8x TOPFlash and M51 Super 8x FOPFlash (TOPFlash mutant) were a gift from Randall Moon (Addgene plasmid # 12456) (Veeman et al., 2003)
[9] ptkRL, originally from Promega, was a kind gift from Dr. Richard Treisman (Francis Crick Institute, London).

Extended Experimental Procedures

Plasmids

Plasmids (see Table S1) were generated from human Tankyrase (TNKS, NM_003747.2), Tankyrase 2 (TNKS2, NM_025235.3), AXIN1 (NM_003502.3) and *D. melanogaster* Ph (NM_057523.5) cDNAs by standard recombinant DNA techniques involving PCR, restriction endonucleases and ligation-independent cloning (Li and Elledge, 2007). For full-length mammalian expression constructs, the initiator methionine codon was omitted. PCRs, including those for site-directed mutagenesis, were performed using KAPA HiFi HotStart DNA polymerase (KAPA Biosystems). Site-directed point and deletion mutant derivatives and chimeric constructs were obtained by using either a modified QuikChange protocol (Agilent Technologies), a two-step megaprimer method or overlap extension. SAM domain deletions in TNKS and TNKS2 encompassed the equivalent regions in both proteins: TNKSΔSAM (Δ1026-1091), TNKS2 ΔSAM (Δ873-938). TNKS2 ΔARC1-5 lacks amino acids 23-794. The TNKS2 xx3xx (L92W, L245W, L560W, L713W) construct was reported previously (Guettler et al., 2011). In the TNKS/TNKS2 SAM domain chimeras, residues 1025-1093^{T1} and 873-940^{T2}, which span the variable range within the SAM domains of TNKS and TNKS2, were mutually exchanged. The TNKS2 Ph SAM domain chimera was generated by replacing TNKS2 residues 876-936 by Ph residues 1504-1577 (numbering for *D. melanogaster* ph-p, transcript variant A, NM_057523.5). Two Tankyrase-binding motifs were mutated in AXIN1 GG-RR (GG27/74RR). All other mutant derivatives are named by their respective mutations. All constructs were sequence-verified.

Antibodies and compounds

Antibodies were anti-MYC 9E10 (MA1-81358, Thermo Fisher Scientific), anti-FLAG FG4R (MA1-91878, Thermo Fisher Scientific), anti-FLAG M2 (F3165, Sigma), anti-AXIN1 (C76H11, 2087S, Cell Signaling Technology), anti-AXIN2 (76G6, 2151S, Cell Signaling Technology), anti-PAR (rabbit polyclonal, 4336-BPC-100, Trevigen), anti-TNKS1/2 (H-350, sc-

8337, Santa Cruz Biotechnology), anti-α-tubulin (TU-01, MA1-19162, Thermo Fisher Scientific), anti-α/β-tubulin (2148S, Cell Signaling Technology), and control IgG (sc-2027, Santa Cruz Biotechnology). Secondary antibodies for Western blotting with detection using an Odyssey infrared imaging system (LI-COR) were goat-anti-mouse-DyLight680 (35518, Thermo Fisher Scientific), goat-anti-rabbit-DyLight800 (35571, Thermo Fisher Scientific), IRDye 800CW donkey anti-mouse (926-32212, LI-COR) and IRDye 800CW donkey anti-rabbit (926-32213, LI-COR). Secondary antibodies for Western blotting with ECL detection were goat-anti-mouse-HRP (32430, Thermo Fisher Scientific) and goat-anti-rabbit-HRP (32460, Thermo Fisher Scientific). Secondary antibodies for immunofluorescence microscopy were goat-anti-mouse-DyLight488 (35502, Thermo Fisher Scientific) and goat-anti-rabbit-DyLight633 (35562, Thermo Fisher Scientific). XAV939 was obtained from Dr. Chris Lord (ICR, London).

Mammalian cell culture

HEK293T and SW480 cells, obtained from Dr. Chris Lord (ICR, London), and HeLa cells, obtained from Dr. Chris Bakal (ICR, London), were cultured in a humidified incubator at 37 °C with 5% CO_2 in Dulbecco's Modified Eagle's Medium (DMEM) supplemented with antibiotics (streptomycin sulfate, benzylpenicillin) and 10% FBS (F7524, Sigma). Cells were serum-starved (0.3% FBS) where indicated.

Luciferase reporter assays

The TOPFlash reporter construct contains six TCF/LEF transcription factor binding sites and responds to active β-catenin (Veeman et al., 2003). On day one, HEK293T cells were plated on white 96-well plates (30,000 cells/well). On day two, cells were transfected, in technical triplicate, with the indicated vector (pLP-dMYC SD, pLP-tripleFLAG SD), Tankyrase constructs in pLP-dMYC SD (16 ng/well or as indicated) or the specified amounts of AXIN1 constructs in pLP-tripleFLAG SD, 10 ng/well TOPFlash or FOPFlash, and 2 ng/well ptkRL. DNA was filled up to a

total amount of 50 ng/well using pDNR-MCS SA. Cell media were changed for 100 μl Opti-MEM II (Thermo Fisher Scientific / Gibco), and cells were transfected using Lipofectamine 2000 (Thermo Fisher Scientific / Invitrogen) in a DNA:transfectant ratio of 1:3 in Opti-MEM II. Four h after complex addition, media were changed for DMEM with 0.3% FBS. XAV939 was added in a two-fold dilution series from 9.8 nM to 10 μM at the media change step, maintaining a constant DMSO concentration of 0.2%. Twenty h after media change, cells from two technical replicates were lysed using Passive Lysis Buffer (Promega) and processed for luminometry using the Dual-Luciferase Reporter Assay system (Promega). Plates were read using a Perkin Elmer VICTOR X5 plate reader using an integration time of 5 s. Upon background subtraction, ratios of Firefly Luciferase to Renilla Luciferase signals were calculated for each of the two technical replicates. The means of the technical replicates were further analysed as indicated in the figure legends. Data shown are from at least three independent experiments performed in technical duplicate, as detailed in the figure legends. A third technical replicate was processed for analysis by SDS-PAGE and Western blotting to assess protein levels (see Figure S1). Immunodetection in Western blots was done using an Odyssey infrared imaging system (LI-COR) or ECL.

Expression and purification of TNKS and TNKS2 SAM domains for crystallisation, electron microscopy, multi-angle light scattering, circular dichroism spectroscopy and isothermal titration calorimetry

Human TNKS SAM (1018-1093) and TNKS2 SAM (867-940) domain constructs were expressed as His_6-MBP-Asn_{10} fusion proteins in *E. coli* BL21-CodonPlus(DE3)-RIL (Stratagene) grown in TB media. Expression was induced at an OD_{600} of 2.0 with 0.5 mM IPTG overnight at 18 °C. Cells were collected by centrifugation, resuspended in a buffer containing 50 mM Tris-HCl (pH 7.5), 1.5 M NaCl (high to limit SAM domain polymerisation), 5 mM imidazole (pH 7.5), 10 mM β-mercaptoethanol and protease inhibitors (1 mM PMSF, 1 μg/ml leupeptin, 1 μg/ml aprotinin, 1 μg/ml pepstatin A) (pellet from 1 l of culture resuspended in 50 ml of buffer), lysed by

homogenisation using an EmulsiFlex-C5 homogenizer (Avestin) or by sonication using a Vibra-Cell sonicator (Sonics & Materials), and centrifuged to remove insoluble material. Lysates were briefly sonicated to shear *E. coli* genomic DNA and filtered through a 0.45 μm filter. Filtered lysates were loaded onto 5 ml Ni HisTrap HP affinity columns (GE Healthcare). Columns were washed with at least 5 column volumes (CV) of wash buffer (identical to lysis buffer but lacking protease inhibitors). His_6-MBP fusion proteins were eluted with a linear imidazole gradient (5 to 250 mM imidazole, pH 7.5) in a buffer also containing 50 mM Tris-HCl (pH 7.5), 1.5 M NaCl and reducing agent. To remove the His_6-MBP tag, the fusion proteins were incubated with recombinant TEV protease overnight while dialysing against 50 mM Tris-HCl (pH 7.5), 200 mM NaCl (1.5 M NaCl for TNKS SAM T1049R and TNKS2 SAM), 10 mM β-mercaptoethanol. The His_6-MBP-Asn_{10} tag was removed by another Ni affinity chromatography step using a 5 ml Ni HisTrap HP affinity column (GE Healthcare), with the exception of TNKS SAM T1049R and TNKS2 SAM. The latter two were diluted 10-fold and incubated for ≈48 h with 25 ml HisPur Ni-NTA Superflow Agarose (Thermo Fisher Scientific) to efficiently remove the His_6-MBP tag entrapped by the polymerising proteins and further dialysed against 50 mM Tris-HCl (pH 7.5), 200 mM NaCl, 10 mM β-mercaptoethanol. All proteins were applied onto 5-ml HisTrap Q HP columns (GE Healthcare) for ion-exchange chromatography. The proteins were eluted in a linear NaCl gradient (0.2 to 1 M NaCl) in a buffer also containing 50 mM Tris-HCl (pH 7.5) and 10 mM β-mercaptoethanol. The resulting protein was dialysed against 25 mM HEPES-NaOH (pH 7.5), 1.5 M NaCl, 10 mM β-mercaptoethanol, concentrated and subjected to size-exclusion chromatography on a 120-ml HiLoad 16/60 Superdex 75 (prep grade, GE Healthcare) equilibrated in 25 mM HEPES-NaOH (pH 7.5), 1.5 M NaCl, 2 mM TCEP. Pure fractions were pooled, concentrated and flash-frozen in liquid nitrogen. *D. melanogaster* Ph SAM domain (1502-1587) was purified as TNKS/TNKS2 SAM, but instead of 1.5 M NaCl, only 500 mM NaCl were used. Before experiments, proteins were dialysed against 25 mM HEPES-NaOH (pH 7.5), 200 mM NaCl, 2 mM TCEP overnight to lower the NaCl concentration kept high to limit polymer formation during

purification. Proteins were quantified spectrophotometrically, using extinction coefficients calculated by ExPASy ProtParam (Gasteiger et al., 2005), and by amino acid analysis (Protein & Nucleic Acid Chemistry Facility, Department of Biochemistry, University of Cambridge, UK). Given that the Tankyrase SAM domains only contain a single aromatic residue enabling A_{280} measurements, spectrophotometrically measured SAM domain concentrations were corrected by a calibration factor based on amino acid analysis performed earlier.

Protein crystallisation

Initial crystal hits of TNKS2(867-940) DH902/924RE^{T2} obtained from the Index HT sparse-matrix screen (Hampton Research) were optimised by mixing 1 µl of a 13.5 mg/ml protein solution (in 25 mM HEPES-NaOH (pH 7.5), 100 or 200 mM NaCl, 2 mM TCEP) with 1 µl of a precipitant solution containing 0.1 M Tris-HCl (pH 8.5), 0.2 M ammonium acetate, 20% PEG 3350 in a hanging-drop vapour-diffusion setup at 12 °C, using a 24-well setup and 1 ml of precipitant solution in the wells. Streak seeding with horse tail hair (Nenê) was performed one day after setting up crystallisation trays with seeds from crystals obtained earlier. Crystals grew within 2 days upon seeding. Before flash-freezing in liquid nitrogen, crystals were cryo-protected in a stabilisation solution identical to the precipitant solution but also containing 30% PEG 400.

Crystals for TNKS SAM (1018-1093) D1055R^{T1} were grown by sitting-drop vapour diffusion at 12 °C using the Index HT (Hampton Research) sparse matrix screen by mixing 150 nl of protein solution at 10 mg/ml (in 25 mM HEPES-NaOH (pH 7.5), 200 mM NaCl, 2 mM TCEP) with 150 nl of a precipitant solution containing 0.2 M $MgCl_2$, 0.1 M Bis-Tris (pH 5.5), 25% PEG 3350. Crystals grew within 3 weeks. Before flash-freezing in liquid nitrogen, crystals were cryo-protected in a stabilisation solution identical to the precipitant solution but also containing 25% ethylene glycol.

Data collection, structure determination and structure analyses

Diffraction data for TNKS2 SAM DH902/924RE^{T2} were collected at the Diamond Light Source on beamline IO3. Data were processed and scaled using XDS (Kabsch, 2010) and merged using AIMLESS (Winn et al., 2011). The structure was solved by molecular replacement using Phaser (Adams et al., 2010; McCoy et al., 2007) with a homology model generated by Modeller/HHPred (Šali et al., 1995) based on 5 templates (PDB codes: 1v85_A, 3bs5_B, 3bq7_A, 2e8o_A, 2gle_A) as a search model. The structure model was generated iteratively by manual model building in Coot (Emsley et al., 2010) and refinement using Phenix Refine (Adams et al., 2010) and BUSTER (Bricogne et al., 2016).

Diffraction data for two crystal forms obtained for TNKS SAM D1055R^{T1} were collected at the Diamond light source on beamline IO3, processed using XDS (Kabsch, 2010) and scaled and merged using AIMLESS (Collaborative Computational Project, 1994). For TNKS SAM crystal form 1, five datasets from a total of three crystals were analysed using BLEND (Foadi et al., 2013) giving a Linear Cell Variation of 0.7 Å. BLEND was subsequently run in synthesis mode on all datasets and merging statistics indicated a high data resolution cutoff of 2.5 Å in order to achieve a half-dataset correlation coefficient CC(1/2) of 0.3 (Karplus and Diederichs, 2012). A second TNKS SAM crystal form was processed using merged data from two crystals as above. The structure was solved by molecular replacement in Phaser (Adams et al., 2010; McCoy et al., 2007) using a homology model for TNKS SAM D1055R^{T1}, generated by SWISS-MODEL (Arnold et al., 2006), based on the crystal structure of TNKS2 SAM DH902/924RE^{T2}. The structure model was generated iteratively by manual model building in Coot (Emsley et al., 2010) and refinement using Phenix Refine (Adams et al., 2010) and BUSTER (Bricogne et al., 2016). High-resolution cut-offs were defined as described by Karplus and Diederichs (Karplus and Diederichs, 2012). During refinement, side chain atoms not accounted for by density due to residue mobility were removed. Structure coordinates and experimental structure factors have been deposited in the Protein Data Bank (PDB IDs 5JRT, 5JU5 and 5JTI).

The structural representations were generated and structural analyses performed using UCSF Chimera (a product of the Resource for Biocomputing, Visualization, and Informatics at the University of California, San Francisco, supported by NIGMS P41-GM103311) (Pettersen et al., 2004). For the electrostatics analysis shown in Figure 3B, the D902R^{T2} and H924E^{T2} mutations were reverted to wild-type; incompletely resolved side-chains were added in full, and the structure was energy-minimised using UCSF Chimera. Interface residues in SAM-SAM domain pairs for Figures 4D, 4E and S7B were identified using the PISA web server (Krissinel and Henrick, 2007). Head-to-tail SAM-SAM domain contacts were analysed using the 'Find Clashes/Contacts' function in UCSF Chimera (allowed overlap: -0.4 Å; H-bond overlap reduction: 0) (Pettersen et al., 2004). Note that PISA defines interface residues on the basis of (at least partial) solvent inaccessibility. Thus, not every interface residue will be involved in an explicit contact.

Bioinformatics analyses

Sequences of Tankyrase orthologues used for the multiple sequence alignment in Figure 4D have the following NCBI accession numbers (in the same order as within the figure): NP_003738.2 (Homo sapiens), NP_780300.2 (*Mus musculus*), NP_989671.1 (*Gallus gallus*), XP_012428885.1 (*Taeniopygia guttata*), XP_004911090.1 (*Xenopus tropicalis*), XP_005451454.1 (*Oreochromis niloticus*), XP_003445711.1 (*Oreochromis niloticus*), NP_079511.1 (*Homo sapiens*), NP_001157107.1 (*Mus musculus*), NP_989672.1 (*Gallus gallus*), XP_012429997.1 (*Taeniopygia guttata*), NP_001017008.2 (*Xenopus tropicalis*), XP_005471626.1 (*Oreochromis niloticus*), XP_687410.4 (*Danio rerio*), NP_001082884.1 (*Danio rerio*), NP_651410.1 (*Drosophila melanogaster*), XP_002121662.3 (*Ciona intestinalis*), XP_001897965.1 (*Brugia malayi*), XP_789260.3 (*Strongylocentrotus purpuratus*), XP_005099438.1 (*Aplysia californica*), CDS23197.1 (*Echinococcus granulosus*), XP_006825651.1 (*Saccoglossus kowalevskii*), XP_012563232.1 (*Hydra vulgaris*), XP_011410275.1 (*Amphimedon queenslandica*). Clear TNKS and TNKS2 orthologues seem to first appear in the fish *Oreochromis niloticus*, which has two

TNKS-like Tankyrases and one TNKS2-like Tankyrase. Another fish species, *Danio rerio*, only appears to have two TNKS-like Tankyrases. Conservation-based and structure-based sequence alignments were generated using ClustalX (Larkin et al., 2007) and UCSF Chimera (Pettersen et al., 2004), respectively. Alignments, coloured by % identity, were visualised using Jalview (Waterhouse et al., 2009). For Figure S7B, the SAM domain sequence of *Drosophila melanogaster* Polyhomeotic (NM_057523.5) was obtained from the SAM domain crystal structure (PDB accession code 1KW4) (Kim et al., 2002).

Ultracentrifugation sedimentation assays

SAM domains were purified as described above (see "Expression and Purification of TNKS and TNKS2 SAM Domains …", for the experiment shown in Figures 2A and S2C) or affinity-purified on a small scale using amylose resin (NEB) (for experiments shown in Figures 3C and 5A). 50 μl of a 25 μM solution (approximately 25 μM for experiments shown in Figures 3C and 5A) of SAM domains (in 25 mM HEPES-NaOH (pH 7.5), 200 mM NaCl, 2 mM TCEP for Figure 2 and 50 mM Tris-HCl (pH 7.5), 200 mM NaCl, 10 mM β-mercaptoethanol for Figures 3 and 5) were subjected to centrifugation at 200,000 x g (average speed) at 20 °C for 1 h in a TLA100 rotor (Beckman Optima TLX centrifuge). Supernatants were removed and pellets resuspended in 50 μl of SDS sample buffer. Equivalent amounts of total, supernatant and pellet samples were analysed by SDS-PAGE on Tris-Tricine gels and Coomassie Brilliant Blue staining.

Circular dichroism spectroscopy

Proteins at 0.2 mg/ml were dialysed into 10 mM Tris-HCl (pH 7.5), 200 mM NaF. CD spectra were collected on a Jasco J-720 spectrometer using a 0.1 cm pathlength cell at the ISMB Biophysics Centre (London). Spectra were averaged over 5 scans and corrected for buffer baseline using CDtool (Lees et al., 2004). The analysis of the spectra was performed in DICHROWEB using the CDSSTR algorithm with SP175 as the reference set (Whitmore and Wallace, 2004; 2008).

Electron microscopy

3 µl of purified TNKS2 SAM domains at 25 µM or 100 µM and TNKS SAM domains at 25 µM, 0.25 mM, 0.5 mM, 1 mM or 2 mM (in 25 mM HEPES-NaOH (pH 7.5), 200 mM NaCl, 2 mM TCEP) were applied to glow-discharged carbon-coated grids and negatively stained with 2% (w/v) uranyl acetate. Electron micrographs were recorded at magnifications of either 11,000x (Figures 2 and S2) or 42,000x (Figures 5, S3 and S7) on an FEI Tecnai 12 electron microscope operating at an accelerating voltage of 120 kV, equipped with an F114 1k x 1k CCD detector (TVIPS, Germany).

SEC-MALS

20 µl of 0.5 mM, 1 mM or 2 mM protein samples were resolved by size exclusion chromatography on an Agilent Prostar HPLC system with a TSKgel G2000SWxl or G3000SWxl column (Tosoh Biocience LLC). Separation was performed in 25 mM HEPES-NaOH (pH 7.5), 200 mM NaCl, 2 mM TCEP at a flow rate of 1 ml/min. In-line light scattering was measured using DAWN Heleos-II light scattering instrument (Wyatt) and differential refractive index using an Optilab rEX instrument (Wyatt). Data analysis, using the Zimm light scattering model and a dn/dc of 0.185 ml/g, was performed using Wyatt's ASTRA software. Weight-average molecular weights (M_w) and dispersities (Đ) with standard deviations were calculated for the elution peak areas in ASTRA. Two separate experiments were performed, and weighted averages of M_w and Đ and associated SD calculated. Weighting was performed by the number of data slices i. Plotted mean and error bars (SD) in scatter plots refer to molecular weights measured in individual slices (data points) M_i. Note that strongly polymerising SAM domains show a lower dRI signal, which is due to a more spread-out elution behaviour. The average SAM domain concentrations (from dRI measurements) in the selected peak areas for the wild-type SAM domain proteins analysed for Figure 2C were as follows: 0.5 mM TNKS SAM sample: 29 µM; 2 mM TNKS SAM sample: 108 µM; 0.5 mM TNKS2 SAM sample: 10 µM. The surprisingly late elution of TNKS2 SAM and TNKS SAM T1049R[T1] may indicate an interaction or entanglement of the long polymers with the

solid phase of the gel filtration column. An earlier elution peak close to the void volume did not contain any SAM domain (see Figure S3B).

Isothermal titration calorimetry

All proteins were dialysed in parallel into binding buffer containing 25 mM HEPES-NaOH (pH 7.5), 200 mM NaCl, 1 mM TCEP. Titrations were carried out at 25 °C on an ITC200 MicroCalorimeter (Microcal/GE Healthcare). TNKS2 SAM Y920A^{T2} or TNKS SAM Y1073A^{T1} at 500 µM were serially injected in 2-µl increments into TNKS2 SAM V903W^{T2} or TNKS SAM V1056W^{T1} at 50 µM or buffer only. ITC data were processed using the Origin7 software (MicroCal). For all calculations, the signals obtained upon titrating injectant into buffer were subtracted. Integrated data were fitted using a one-site binding model.

TNKS2 auto-PARylation assays and PAR analysis

HEK293T cells were seeded on 15-cm cell culture dishes at 9 x 10^6 cells per dish. On the next day, cells were transfected with 30 µg per dish of pLP-dMYC SD empty vector or the indicated TNKS2 constructs (2 dishes per construct) using calcium phosphate. 24 h post-transfection, cells were scraped in ice-cold PBS and cell pellets lysed in 1 ml high-salt RIPA buffer (50 mM HEPES-NaOH (pH 7.5), 750 mM NaCl, 1% Triton X-100, 0.5% sodium deoxycholate, 0.1% SDS, 1 mM DTT, 2 µM ADP-HPD PARG inhibitor (Merck) and protease inhibitors (Pierce protease inhibitor tablets, EDTA-free, Thermo Fisher Scientific). Cell lysates were briefly sonicated on ice to shear DNA and cleared by centrifugation (20,817 xg, 15 min) at 4 °C. The cleared cell lysates were incubated with 75 µl (packed volume) of pre-equilibrated anti-c-Myc-agarose resin (9E10; Thermo Fisher Scientific or Takara Bio) for 3 h, rotating at 4 °C. Resin samples were washed 9 times with 4 ml lysis buffer and 3 times with 1 ml PARP assay buffer (50 mM HEPES–NaOH (pH 7.5), 150 mM NaCl, 0.01% Triton X-100, 10% glycerol, 1 mM DTT). After the final wash step, 75 µl of PAR assay buffer were added to the resin and 40 µl of the suspension removed

for analysis by SDS-PAGE and Commassie Brilliant Blue staining to estimate protein levels. The remaining samples were centrifuged and the buffer removed. Sample volumes were adjusted to 54 μl by addition of PARP assay buffer, estimating that the resin after buffer removal entraps approximately half its volume of buffer. 6 μl of 10x NAD^+ stock (10 mM NAD^+ and 5 μCi/6 μl ^{32}P-NAD^+ (Perkin Elmer)) were added to each sample and PARP reactions performed for 30 min at 30 °C on a horizontal shaker at 800 rpm. 30 μl of suspension were taken from each sample, boiled with 2x SDS sample buffer and comparable amounts of MYC_2-Tankyrases, based on previous protein level estimates, analysed by SDS-PAGE and Coomassie Brilliant Blue staining. The gel was dried on a gel dryer and exposed to a phosphoimager plate (GE Healthcare) for 24 h. If MYC_2-Tankyrase levels were low, their levels in the PARylation reaction were analysed by Western blotting, using equivalent amounts of immunoprecipitate samples set aside prior to the *in-vitro* PARylation reaction. Endogenous PARylation (prior to the *in-vitro* PARylation reaction) was assessed by Western blotting using an anti-PAR antibody.

PAR chains were analysed essentially as described previously (Alvarez-Gonzalez and Jacobson, 1987; Panzeter and Althaus, 1990), as follows. The PARP assay samples remaining upon SDS-PAGE analysis were precipitated by addition of an equal volume of ice-cold 40% (w/v) trichloroacetic acid (TCA) and incubated on ice for 15 min. The resins were settled by slow centrifugation (1,000 xg, 10 s) and the supernatants recovered. Precipitates were collected by centrifugation (16,300 xg, 10 min) and the pellets washed 3 times with 100 μl 5% TCA followed by 2 wash steps with 100 μl ice-cold diethyl ether (Sigma-Aldrich). The pellets were dried using a Micro-Cenvac NB-503CIR vacuum concentrator at 50 °C for 10 min at 2,500 rpm. The PAR chains were detached from the precipitated material by resuspending the dried pellets in 100 μl of 10 mM Tris (base), 1 mM EDTA, pH 12 and incubated for 3 h at 60 °C. PAR was extracted with 100 μl of phenol/chloroform/isoamyl alcohol (25:24:1) and the aqueous phase recovered followed by two rounds of phenol back-extraction with 100 μl chloroform/isoamyl alcohol (24:1). After the final phenol back-extraction, the samples were dried in the vacuum concentrator at 50 °C for 60 min at

2,500 rpm. PAR was dissolved in 10 μl of PAR sample buffer (50% urea, 25 mM NaCl, 4 mM EDTA (pH 8.0), 0.02% xylene cyanol, 0.02% bromophenol blue). The amounts of ^{32}P-labelled PAR chains were quantified by Cerenkov counting and equal counts per minute (cpm) of samples were loaded onto a 40% acrylamide/bisacrylamide (19:1) sequencing gel (in a kuroGEL Verti 1824 apparatus), except for the negative control samples (vector only and TNKS2 G1032W^{T2}), for which the whole sample was loaded. The sequencing gel electrophoresis was performed in 1x TBE running buffer using a constant current of 25 mA. The electrophoresis was stopped when the bromophenol blue band had migrated 11 cm from the bottom of the wells. The gel was fixed for 1 h with 40% methanol, 10% acetic acid, 3% glycerol (to protect it from cracking during drying) and dried using a temperature gradient cycle to 60 °C for 2-3 h. The gel was exposed for 24 h to a phosphoimager plate (GE Healthcare). Phosphoimager plates were read using a Typhoon FL9500 biomolecular imager (GE Healthcare) and analysed using ImageQuant TL. Xylene cyanol and bromophenol blue were used to determine the PAR chain length (Alvarez-Gonzalez and Jacobson, 2011).

Tankyrase-Tankyrase co-immunoprecipitation

HEK293T cells were seeded on 10-cm cell culture dishes at 6 x 10^6 cells per dish. On the next day, cells were transfected with expression plasmids for pLP-tripleFLAG SD-TNKS (wild-type or T1049R^{T1}) or pLP-tripleFLAG SD-TNKS2 (wild-type or R896T^{T2}) as bait or empty vector as control (10 μg) and co-transfected with the indicated pLP-dMYC SD-TNKS or pLP-dMYC SD-TNKS2 constructs (10 μg) using calcium phosphate. After 24 h, cells were scraped in ice-cold PBS and collected by centrifugation. Cells were lysed in 900 μl of lysis buffer containing 50 mM HEPES-NaOH (pH 7.5), 200 mM NaCl, 0.2% Triton X-100, 10% glycerol, 5 mM DTT and protease inhibitors (Pierce protease inhibitor tablets, EDTA-free, Thermo Fisher Scientific). Cell lysates were briefly sonicated on ice to shear genomic DNA and clarified by centrifugation at 20,817 xg for 15 min at 4 °C. Immunoprecipitation was performed with 30 μl of packed,

equilibrated anti-FLAG M2 agarose (Sigma), rotating for 3 h at 4 °C. The immunoprecipitates were washed 5 times with 1 ml lysis buffer (without protease inhibitors and with only 1 mM DTT). Immunoprecipitated proteins were recovered by boiling the resin in 50 μl of 2x SDS-PAGE sample buffer. Lysate samples (input) and immunoprecipitates were analysed by SDS-PAGE (10%) and Western blotting. Western blots were imaged using an Odyssey infrared imaging system (LI-COR).

Endogenous AXIN1 immunoprecipitation

HEK293T cells were seeded on 10-cm cell culture dishes at 6 x 10^6 cells per dish. On the next day, the media were replaced with DMEM containing 0.3% FBS. (Serum starvation was performed to match conditions used in the luciferase reporter assays.) Each dish was transfected with 10 μg of pLP-dMYC SD empty vector or the indicated TNKS2 constructs using calcium phosphate. In order to maintain its limiting cellular concentrations, we did not overexpress AXIN bait protein but instead relied on endogenous AXIN1. 24 h post-transfection, cells were scraped in ice-cold PBS and cell pellets lysed in 0.75 ml of the stringent RIPA buffer (50 mM HEPES-NaOH (pH 7.5), 150 mM NaCl, 1% Triton X-100, 0.5% sodium deoxycholate, 0.1% SDS, 1 mM DTT, 2 μM ADP-HPD PARG inhibitor (Merck) and protease inhibitors (Pierce protease inhibitor tablets, EDTA-free, Thermo Fisher Scientific). Cell lysates were briefly sonicated on ice to shear DNA and cleared by centrifugation (20,817 xg, 15 min) at 4 °C. Cleared cell lysates were incubated rotating at 4 °C overnight with rabbit anti-AXIN1 (C76H11 clone, Cell Signaling Technologies) at a 1:50 dilution, as recommended by supplier. Rabbit IgG antibody (sc-2027, Santa Cruz Biotechnology), at comparable concentration, was used as a negative control. 25 μl of pre-equilibrated Pierce Protein A/G magnetic resin (Thermo Fisher Scientific) were incubated with the samples for 2 h rotating at 4 °C. The resin samples were washed extensively: 6 times with 1 ml RIPA buffer (without ADP-HPD and protease inhibitors) each. Immunoprecipitated proteins were recovered by boiling the resin samples in 60 μl of 2x SDS-PAGE sample buffer. Lysate samples (input) and

immunoprecipitates were analysed by SDS-PAGE and Western blotting. Western blots were imaged using an Odyssey infrared imaging system (LI-COR).

Assessment of Tankyrase inhibition by XAV939

HEK293T cells were seeded on six-well cell culture dishes at 3.5 x 10^5 cells per well. On the next day, cells were transfected in Opti-MEM II (Thermo Fisher Scientific / Gibco) with expression plasmids for pLP-dMYC SD-TNKS2 or empty vector as control (1 μg) using Lipofectamine 2000 in a DNA:transfectant ratio of 1:3. Four h after transfection complex addition, media were changed for DMEM with 0.3% FBS, to match conditions of the luciferase reporter assay. XAV939 was included in a two-fold dilution series from 9.8 nM to 10 μM at the media change step, maintaining a constant DMSO concentration of 0.2%. Twenty h after XAV939 treatment, cells were lysed in 50 mM HEPES-NaOH (pH 7.5), 150 mM NaCl, 0.1% SDS, 0.5% sodium deoxycholate, 1% Triton X-100, 5 mM DTT, 2 μM ADP-HPD PARG inhibitor (Merck) and protease inhibitors (Pierce protease inhibitor tablets, EDTA-free, Thermo Fisher Scientific). Cell lysates were cleared by centrifugation (20,817 xg, 15 min) at 4 °C. MYC_2-TNKS2 was immunoprecipitated using 20 μl of packed, equilibrated anti-MYC 9E10 agarose (Takara) per sample on a rotating wheel for 3 h at 4 °C. Immunoprecipitates were washed 3 times with 1 ml wash buffer (as lysis buffer, but without PARG inhibitor, protease inhibitors and containing only 1 mM DTT). Resin samples were taken up in 20 μl 4x SDS-PAGE sample buffer, boiled and processed for SDS-PAGE and Western blotting.

Cell lysate fractionations by gel filtration chromatography

HEK293T cells were seeded on 10-cm cell culture dishes at 6 x 10^6 cells per dish. On the next day, cells were transfected with the indicated pLP-dMYC SD-TNKS or pLP-dMYC SD-TNKS2 constructs or empty vector (20 μg) using calcium phosphate. Cells were maintained in DMEM with 10% FBS. After 24 h, cells were washed in ice-cold PBS, scraped in ice-cold PBS, collected by centrifugation and flash-frozen in liquid nitrogen. Cells were lysed in 600 μl of

lysis/fractionation buffer containing 50 mM HEPES-NaOH (pH 7.5), 200 mM NaCl, 0.2% Triton X-100, 2 mM TCEP and protease inhibitors (Pierce protease inhibitor tablets, EDTA-free, Thermo Fisher Scientific). Lysates were briefly sonicated (3 s at 20% output on a Vibra-Cell sonicator (Sonics & Materials) equipped with a micro-tip). After 10 min extraction time on ice, cell lysates were cleared by centrifugation (20,817 xg, 15 min) at 4 °C. 500 μl of cleared lysates were subjected to size exclusion chromatography on a Superose 6 10/300 GL column (GE Healthcare) equilibrated in lysis/fractionation buffer (without protease inhibitors, which were only included in the sample) with a flow rate of 400 μl/min and collection of 400-μl fractions. 200 μl of each fraction were subjected to acetone precipitation with 800 μl acetone at -20 °C. Precipitates were collected by centrifugation (20,817 xg, 10 min) at 4 °C, air-dried and taken up in 10 μl of 2x SDS-PAGE sample buffer. 5 μl of lysate (input) and fraction samples (total 10 μl corresponding to 200 μl of fractionated sample) were analysed by SDS-PAGE and Western blotting. The column was calibrated using a gel filtration standard protein mix (BIO-RAD, 151-1901) under identical fractionation conditions. Note that apparent molecular weights of eluting proteins are not only sensitive to polymeric status but also to interactions in the cell lysates and protein/protein complex shape.

Fluorescence microscopy

HeLa or SW480 cells were plated in 6-well dishes with glass coverslips (200,000 cells/well). On the following day, the media were changed for Opti-MEM II (Thermo Fisher Scientific / Gibco), and the cells were transiently transfected with 1 μg of the indicated MYC_2-, mCitrine- or mCherry-tagged TNKS2 constructs each, using Lipofectamine 2000 (Thermo Fisher Scientific / Invitrogen) in a DNA:transfectant ratio of 1:3. 4 h after complex addition, transfection mix was replaced by DMEM containing 0.3% FBS supplemented with either DMSO (0.04%) or 2 μM XAV939. 20 h after media change, cells were fixed by addition of 4% formaldehyde (AMRESCO) and incubation at 37 °C for 10 min. For immunofluorescence microscopy, cells were

washed once with PBS, permeabilised with 0.2% Triton X-100 in PBS for 10 min. Permeabilisation buffer was replaced by PBS. Non-specific epitopes were blocked with blocking solution (PBS, 5% (w/v) dry milk powder, 10% FBS, 0.05% Tween-20) for 1 h. Cells were immunostained with primary (anti-MYC 9E10 (1:1000, MA1-81358, Thermo Fisher Scientific), anti-AXIN2 (1:100, 76G6, Cell Signaling Technology) and secondary antibodies in blocking solution for 1 h, respectively, and DAPI-stained to visualise DNA. DAPI staining without permeabilisation was performed for HeLa cells expressing mCitrine and mCherry fusion proteins. Coverlips were mounted on glass slides using fluorescent mounting media (DAKO). Cells were imaged on an LSM710 confocal laser scanning microscope (Zeiss). Micrographs were acquired in the Zen software (Zeiss) and channels separated using Adobe Photoshop. A uniform exposure adjustment across all panels was performed for Figures 6B and S7C and D to enhance visibility of localisation features in the figures.

Supplemental References

Adams, P.D., Afonine, P.V., Bunkóczi, G., Chen, V.B., Davis, I.W., Echols, N., Headd, J.J., Hung, L.-W., Kapral, G.J., Grosse-Kunstleve, R.W., et al. (2010). PHENIX: a comprehensive Python-based system for macromolecular structure solution. Acta Crystallogr. D Biol. Crystallogr. *66*, 213–221.

Alvarez-Gonzalez, R., and Jacobson, M.K. (1987). Characterization of polymers of adenosine diphosphate ribose generated in vitro and in vivo. Biochemistry *26*, 3218–3224.

Alvarez-Gonzalez, R., and Jacobson, M.K. (2011). Quantification of poly(ADP-ribose) in vitro: determination of the ADP-ribose chain length and branching pattern. Methods Mol. Biol. *780*, 35–46.

Arnold, K., Bordoli, L., Kopp, J., and Schwede, T. (2006). The SWISS-MODEL workspace: a web-based environment for protein structure homology modelling. Bioinformatics *22*, 195–201.

Collaborative Computational Project, N.4. (1994). The CCP4 suite: programs for protein crystallography. Acta Crystallogr. D Biol. Crystallogr. *50*, 760–763.

Colwill, K., Wells, C., Elder, K., Goudreault, M., Hersi, K., Kulkarni, S., Hardy, W.R., Pawson, T., and Morin, G. (2006). Modification of the Creator recombination system for proteomics applications – improved expression by addition of splice sites. BMC Biotechnol *6*, 13.

Emsley, P., Lohkamp, B., Scott, W.G., and Cowtan, K. (2010). Features and development of Coot. Acta Cryst (2010). D66, 486-501 [Doi:10.1107/S0907444910007493] 1–16.

Foadi, J., Aller, P., Alguel, Y., Cameron, A., Axford, D., Owen, R.L., Armour, W., Waterman, D.G., Iwata, S., and Evans, G. (2013). Clustering procedures for the optimal selection of data sets from multiple crystals in macromolecular crystallography. Acta Crystallogr. D Biol. Crystallogr. *69*, 1617–1632.

Francis, N.J., Saurin, A.J., Shao, Z., and Kingston, R.E. (2001). Reconstitution of a functional core polycomb repressive complex. Molecular Cell *8*, 545–556.

Gasteiger, E., Hoogland, C., Gattiker, A., and Wilkins, M.R. (2005). Protein identification and analysis tools on the ExPASy server (Totowa, NJ: Humana Press).

Guettler, S., LaRose, J., Petsalaki, E., Gish, G., Scotter, A., Pawson, T., Rottapel, R., and Sicheri, F. (2011). Structural basis and sequence rules for substrate recognition by Tankyrase explain the basis for cherubism disease. Cell *147*, 1340–1354.

Kabsch, W. (2010). XDS. Acta Crystallogr. D Biol. Crystallogr. *66*, 125–132.

Karplus, P.A., and Diederichs, K. (2012). Linking crystallographic model and data quality. Science *336*, 1030–1033.

Kim, C.A., Gingery, M., Pilpa, R.M., and Bowie, J.U. (2002). The SAM domain of polyhomeotic forms a helical polymer. Nature Structural & Molecular Biology *9*, 453–457.

Krissinel, E., and Henrick, K. (2007). Inference of macromolecular assemblies from crystalline state.

Larkin, M.A., Blackshields, G., Brown, N.P., and Chenna, R. (2007). Clustal W and Clustal X

version 2.0.

Lees, J.G., Smith, B.R., Wien, F., Miles, A.J., and Wallace, B.A. (2004). CDtool-an integrated software package for circular dichroism spectroscopic data processing, analysis, and archiving. Analytical Biochemistry *332*, 285–289.

Li, M.Z., and Elledge, S.J. (2007). Harnessing homologous recombination in vitro to generate recombinant DNA via SLIC. Nat Meth *4*, 251–256.

McCoy, A.J., Grosse-Kunstleve, R.W., Adams, P.D., Winn, M.D., Storoni, L.C., and Read, R.J. (2007). Phaser crystallographic software. J Appl Crystallogr *40*, 658–674.

Panzeter, P.L., and Althaus, F.R. (1990). High resolution size analysis of ADP-ribose polymers using modified DNA sequencing gels. Nucleic Acids Research *18*, 2194.

Patke, S., Maheshwari, R., Litt, J., Srinivasan, S., Aguilera, J.J., Colón, W., and Kane, R.S. (2012). Influence of the carboxy terminus of serum amyloid A on protein oligomerization, misfolding, and fibril formation. Biochemistry *51*, 3092–3099.

Pettersen, E.F., Goddard, T.D., Huang, C.C., Couch, G.S., Greenblatt, D.M., Meng, E.C., and Ferrin, T.E. (2004). UCSF Chimera--a visualization system for exploratory research and analysis. J Comput Chem *25*, 1605–1612.

Šali, A., Potterton, L., and Yuan, F. (1995). Evaluation of comparative protein modeling by MODELLER. Proteins: Structure.

Veeman, M.T., Slusarski, D.C., Kaykas, A., Louie, S.H., and Moon, R.T. (2003). Zebrafish prickle, a modulator of noncanonical Wnt/Fz signaling, regulates gastrulation movements. Curr. Biol. *13*, 680–685.

Waterhouse, A.M., Procter, J.B., Martin, D.M.A., Clamp, M., and Barton, G.J. (2009). Jalview Version 2--a multiple sequence alignment editor and analysis workbench. Bioinformatics *25*, 1189–1191.

Whitmore, L., and Wallace, B.A. (2004). DICHROWEB, an online server for protein secondary structure analyses from circular dichroism spectroscopic data. Nucleic Acids Research *32*, W668–W673.

Whitmore, L., and Wallace, B.A. (2008). Protein secondary structure analyses from circular dichroism spectroscopy: Methods and reference databases. Biopolymers *89*, 392–400.

Winn, M.D., Ballard, C.C., Cowtan, K.D., Dodson, E.J., Emsley, P., Evans, P.R., Keegan, R.M., Krissinel, E.B., Leslie, A.G.W., McCoy, A., et al. (2011). Overview of the CCP4 suite and current developments. Acta Crystallogr. D Biol. Crystallogr. *67*, 235–242.
